# Leveraging Multi‐Isotope Systems to Investigate Conflict, Food Insecurity, and Elevated Nitrogen Isotope Values in the Nazca Highlands of Peru During the Late Intermediate Period (1000–1450 CE)

**DOI:** 10.1002/ajpa.70373

**Published:** 2026-10-30

**Authors:** Sarah J. Noe, Bridgette Degnan, Emily C. Kracht, Weston C. McCool

**Affiliations:** ^1^ South Carolina Institute for Archaeology and Anthropology University of South Carolina Columbia South Carolina USA; ^2^ Department of Anthropology University of California, Santa Barbara Santa Barbara California USA; ^3^ Social Sciences Department California Polytechnic State University San Luis Obispo California USA

**Keywords:** Central Andes, food insecurity, isotope analysis, nitrogen isotopes, population health

## Abstract

**Objectives:**

The physical effects of interpersonal violence are well documented in archaeology, but its broader impacts on population health, mobility, and resource access remain less understood. This article assesses how chronic warfare restricted movement and access to critical resources, altering dietary practices and contributing to persistent food insecurity.

**Materials and Methods:**

We develop a replicable framework for using coupled biological and isotopic data to evaluate elevated δ^15^N values in archaeological populations. The framework is applied to the Nazca highlands of Peru during the Late Intermediate Period (LIP; 1000–1450 ce), a context marked by persistent violence and regional instability. We analyze δ^15^N, δ^13^C, and δ^34^S data from 149 radiocarbon‐dated individuals alongside osteological evidence, paleoclimatic proxies, and dietary baselines.

**Results:**

Nazca highland LIP individuals exhibit elevated δ^15^N values (mean = 13.36‰). Carbon isotopes indicate reliance on C4 resources (mean δ^13^Ccollagen = −10.38‰; mean δ^13^Ccarbonate = −6.98‰), while δ^34^S values are low (mean≈4.1‰). Elevated δ^15^N did not vary by age or sex, while LIP individuals exhibited greater skeletal stress and extensive interpersonal violence.

**Discussion:**

Persistent conflict and restricted mobility altered Nazca highland food production and access. Dependence on land near fortified settlements may have encouraged intensive cultivation and camelid dung fertilization, raising dietary δ^15^N. Reduced mobility also constrained strategies for buffering seasonal shortfalls, increasing recurrent food insecurity and catabolism that may have further enriched human δ^15^N. Consistently elevated nitrogen values therefore reflect subsistence changes associated with sustained warfare and constrained access to land and resources.

## 1 Introduction

The Late Intermediate Period (LIP, ca. cal 1000–1450 ce) marked a major reorganization of political and social life across the central Andes following the decline of Tiwanaku and Wari at the end of the Middle Horizon (MH, ca. cal 600–1000 ce). In the centuries before Inca expansion, communities increasingly operated within localized political landscapes characterized by substantial regional variation in material traditions, political organization, settlement, and land use. In many areas, these changes were accompanied by heightened interpersonal and intergroup violence, reflected in defensive settlement locations, fortified communities, and skeletal evidence of trauma (Arkush and Tung 2013; Covey 2008; Parsons and Hastings 1988). These conditions affected more than political organization alone. Recurrent conflict had the potential to reshape where people lived, how safely they could move through the landscape, and how they accessed the fields, pastures, and other resources on which daily subsistence depended (McCool et al. 2022).

Although the direct consequences of interpersonal violence are well documented archaeologically through trauma and mortality, its broader effects on population health are more difficult to identify (Gat 2009; Glowacki et al. 2020; Glowacki and Wrangham 2013; VanDerwarker and Wilson 2016). Warfare research suggests that the risks associated with interpersonal violence can make travel dangerous, forcing individuals to balance conflict avoidance with access to essential resources (Allen et al. 2016; Glowacki 2024; Macfarlan et al. 2014; McCool et al. 2022). These constraints may be especially consequential for agropastoral populations whose subsistence systems depend on regular movement among agricultural fields, herding zones, and dispersed resource patches. When access to these areas becomes restricted, communities may lose opportunities to maintain diversified food‐production strategies and become more dependent on a narrower or less reliable set of resources (VanDerwarker and Wilson 2016). Violence may therefore influence population health not only through direct injury, but also indirectly by altering local mobility, resource access, and the organization of food production.

Understanding whether these broader conditions affected subsistence and population health requires evidence that can capture changes in diet, resource use, and physiological condition beyond the direct skeletal consequences of violence. Stable isotope analysis provides one such line of evidence because isotope values preserved in human tissues reflect multiple dimensions of an individual's dietary and environmental history (Ambrose 1991; DeNiro 1985; Schoeninger and DeNiro 1984). Carbon isotope values can provide information about major dietary pathways, while sulfur isotopes can help distinguish among resources derived from different environmental settings, including marine contexts. Nitrogen isotope values are likewise sensitive to dietary variation, but their interpretation is particularly complex. Differences in δ^15^N may reflect trophic position, agricultural practices, local environmental baselines, or physiological changes in nitrogen metabolism (Ambrose 1991; DeNiro 1985; Szpak 2014). As a result, elevated δ^15^N values are not diagnostic of a single dietary or physiological process and must instead be evaluated within the broader archaeological, environmental, and biological context in which they occur.

We apply this framework to LIP populations from the Nazca highlands of southern Peru. We combine population‐level osteological evidence for interpersonal violence and physiological stress from 267 individuals with stable isotope data from 149 directly AMS radiocarbon‐dated individuals. The isotopic dataset includes bone collagen δ^13^C and δ^15^N values and bone carbonate δ^13^C values for all 149 individuals, together with δ^34^S values from a subset of 30 individuals. Human bone collagen δ^15^N values are consistently elevated, averaging 13.36‰ (SD = 0.94) through the LIP, creating an unusual pattern that requires further investigation. We therefore evaluate a range of dietary, environmental, demographic, and physiological processes that could contribute to this observed enrichment.

We argue that chronic warfare altered subsistence practices in the Nazca highlands through two related processes that contributed to elevated human δ^15^N values. First, restricted access to dispersed agricultural fields, pastures, and exchange networks likely increased reliance on land surrounding fortified settlements. Concentrating production within a smaller cultivation area may have encouraged more intensive land use, including greater application of camelid dung fertilizer (Eerkens et al. 2024; Kuzminsky et al. 2024; McCool et al. 2024; Szpak et al. 2012; Szpak and Chiou 2020; VanDerwarker and Wilson 2016). Manuring increases plant δ^15^N values and has been identified as an important source of isotopic enrichment in Andean agricultural systems. Second, reduced mobility and exchange would have limited communities' capacity to compensate for seasonal food shortages. Recurrent nutritional stress and associated tissue catabolism may have further increased human δ^15^N values, with this signal incorporated into bone collagen during remodeling (Beaumont and Montgomery 2016; Robertson et al. 2017, 2014; Walter et al. 2020). Increased fertilizer use and reduced food availability have similarly been proposed to explain elevated post‐Wari δ^15^N values in the nearby Ayacucho Basin (Kuzminsky et al. 2024). We therefore evaluate whether the observed δ^15^N enrichment reflects the combined effects of consuming crops grown with camelid dung fertilizer and recurrent nutritional stress, both associated with conflict‐driven restrictions on mobility and resource access.

### 1.1 Regional Background

The LIP was a transformative era for Andean mid‐altitude and highland populations as post‐Wari political fragmentation unfolded alongside climatic instability, resource stress, and escalating internecine conflict (Arkush and Tung 2013; McCool, Tung, et al. 2021). Following the collapse of the Wari State around 1000 ce and preceding the Inca conquest in the 15th CEntury CE, communities living in post‐Wari highlands experienced increased rates of interpersonal violence and many populations aggregated into fortified hilltop settlements known as “pukara” (hillforts) (Arkush 2010; Bauer and Kellett 2010; Ibarra Asencios 2021; Kellett 2010; Washburn et al. 2022).

The Nazca highlands reflect these broader patterns of conflict and settlement reorganization. Situated around 2500 m above the modern city of Nazca, Peru, this landscape is defined by steep, narrow river valleys and limited arable land (Figure 1). During the LIP, pukaras were strategically positioned on inaccessible terrain within the valleys to optimize defense, often clustering just a few kilometers apart (Figure 1) (Arkush and Stanish 2005; McCool 2017; Washburn et al. 2022). People living in this ecologically circumscribed region—situated between the coast and the sierra—relied on the land immediately surrounding their settlements for agriculture, utilizing terraces across steep river valleys and extensive irrigation canals. The nearby Puna grasslands, however, were unsuitable for cultivation but supported herding (Carneiro 1970; McCool 2017). This settlement arrangement maintained proximity to arable land while also creating a buffer zone that isolated these communities from surrounding populations (McCool 2017).

**FIGURE 1:**
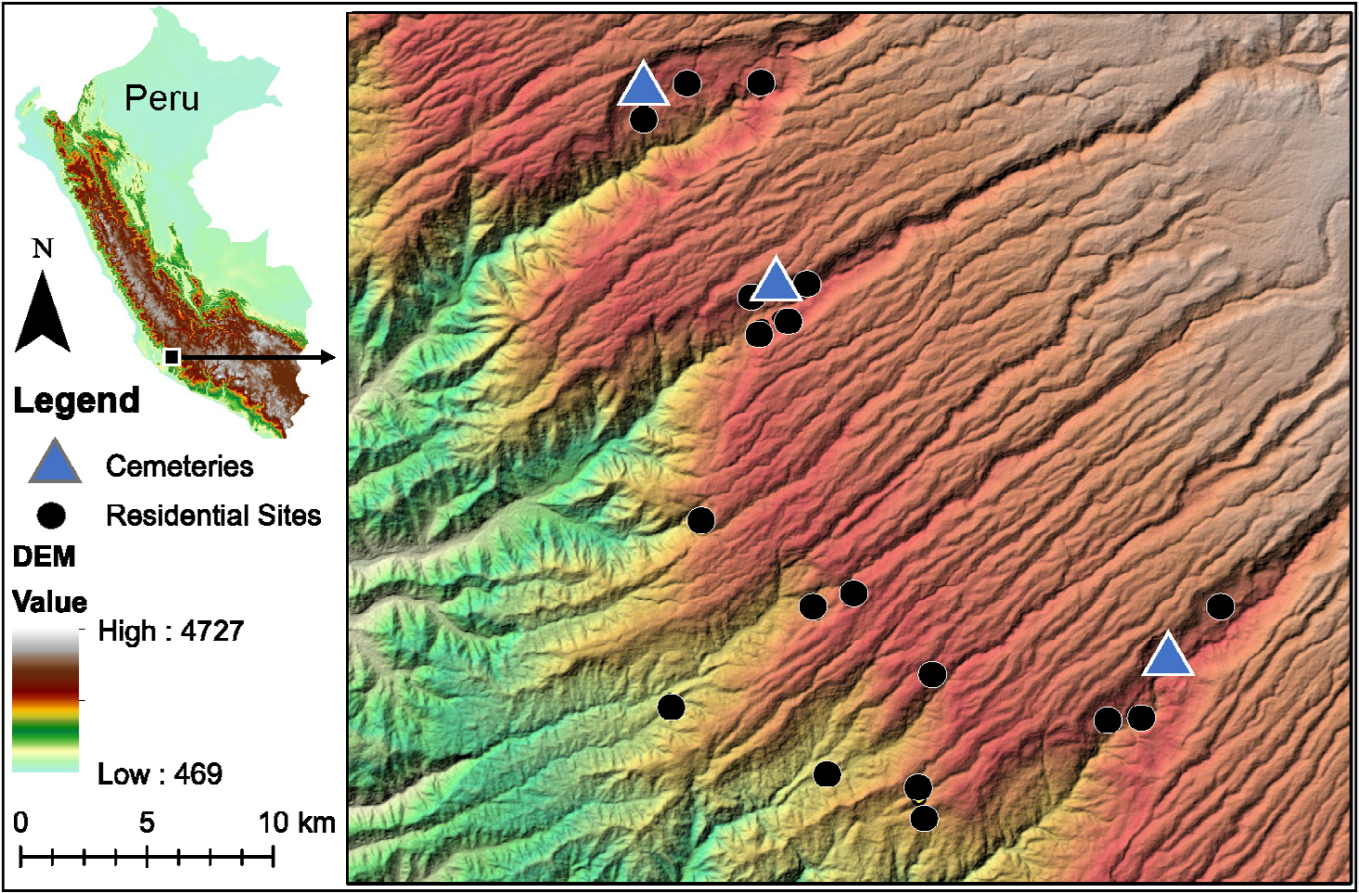
The Nasca highland region with relevant sites and cemeteries.

W.C.M. documented 19 LIP settlements in the Nazca highlands, all permanently occupied (as opposed to transhumant sites such as herding stations or pastoral camps or fortified refuges) as evidenced by dense housing clusters and middens across the pukaras in all five river valleys (McCool, Anderson, and Kennett 2021). Cemeteries adjacent to the hillforts contained several dozen to several hundred artificially constructed tombs known as “chullpas.” Each tomb contained the disarticulated and comingled remains of multiple individuals that were formally wrapped in cotton shrouds and arranged in tight clusters on the ground surface (McCool, Anderson, and Kennett 2021). Despite evidence of organized settlement and collective burial practices, there are no clear indications of substantial sociopolitical hierarchy in either domestic or mortuary architecture. Both settlement and burial patterns exhibit minimal architectural variation, with uniform housing construction and only modest differences in tomb quality, suggesting an absence of social stratification. Excavations conducted by W.C.M. in 2018 and 2019, followed by subsequent osteological and isotope analysis, generated the dataset used in this paper (McCool 2020). Excavations were conducted at three cemetery sites, listed from south‐to‐north: (1) the Cerro Chillca and Cerro Tribolpata cemetery in the Las Tancas Valley, (2) the Cerro Ayapata cemetery in the Tierras Blancas Valley, and (3) the Ayorcco cemetery in the Aja Valley.

### 1.2 Cranial Trauma as Evidence of LIP Internecine Warfare

As discussed above, the LIP across much of the Central Andean highlands was characterized by intense internecine conflict, fortified settlement, and widespread interpersonal violence (Arkush and Tung 2013; Washburn et al. 2022). In the Nazca highlands, prior analysis of human crania from the LIP (*n* = 267) and preceding MH (*n* = 97) revealed that the average number of traumatic injuries per individual rose sharply from 0.10 to 0.80 (Data S2; McCool, Anderson, and Kennett 2021; McCool et al. 2022). Notably, 38% of the LIP population exhibited cranial fractures, with 31% showing signs of ante‐mortem trauma and 13% displaying peri‐mortem trauma, the latter often the result of extreme violence as evidenced by the infliction of excessive and disproportionate force on a victim (Data S2; S1: Section 6; McCool, Anderson, and Kennett 2021). Females, children, and older adults were disproportionately affected by blows to the back of the head, suggesting they were ambushed or attacked while fleeing. In contrast, adolescents and young adults—likely participants in direct conflict—experienced higher rates of fatal injuries. There is no evidence that violence was systematically based on identity or sex; instead, it reflects a broader pattern of social substitutability in which any individual from an opposing group could be indiscriminately targeted (McCool, Anderson, and Kennett 2021).

The LIP saw widespread political fragmentation and escalating conflict across the Peruvian highlands exemplified by cranial trauma rates surpassing 30% in studied skeletal populations (Arkush and Tung 2013; McCool et al. 2022). At Monqachayoq, in the Ayacucho region of south‐central Peru, 71% of adults exhibited antemortem cranial trauma and 42% suffered perimortem trauma (Tung et al. 2016), while at Pukara de Khonkho in the Titicaca Basin, 25% of the population displayed traumatic injuries alongside increased defensive constructions (Zovar 2012). The widespread violence during the LIP, spanning the Nazca and broader Andean highlands, affected multiple populations across the region (Data S1: Section 6).

### 1.3 Skeletal Indicators of Stress During the LIP

Substantial climatic variability during the LIP significantly impacted the Andean highlands, with the Nazca highlands enduring a prolonged drought that severely reduced local moisture (Kennett and Marwan 2015; Schittek et al. 2015). Starting around 1000 ce, the region received less than 50% of its usual precipitation (Schittek et al. 2015). The geographic isolation of the Nazca highlands exacerbated resource strain, as populations relied heavily on river‐fed irrigation for food production (Carneiro 1970; McCool 2017). While prolonged drought conditions persisted throughout the first half of the LIP (~900 to ~1200 ce), the subsequent shift to wetter conditions stimulated rapid population growth and resultant population pressure in the region's circumscribed environment, contributing to increased violence and biological stress (McCool et al. 2024). These environmental pressures likely played a role in widespread physiological stress, as evidenced by high rates of porotic hyperostosis (PH) and cribra orbitalia (CO) (Data S1: Section 6; McCool et al. 2024). CO and PH are nonspecific cranial skeletal l esions that generally develop during childhood and indicate physiological stress. They may reflect a range of underlying processes, including nutritional deficiencies, infectious diseases, parasite load, reduced access to fresh water, or genetic conditions (Anderson et al. 2025; Rivera and Mirazón Lahr 2017; Turner and Armelagos 2012; Walker et al. 2009).

In the Nazca highland population, the prevalence of both CO and PH increased significantly from the MH to the LIP (McCool, Anderson, and Kennett 2021; McCool et al. 2024). During the MH, CO affected 8.2% of individuals, while PH was observed in 5.2%. By the LIP, there was a statistically significant rise in both types of lesions, with CO impacting 17.8% of individuals and PH affecting 26.7% across the ~500‐year span (McCool, Tung, et al. 2021; McCool et al. 2024). During the early phase of the LIP (950–1200 ce), CO was present in 10% of individuals, briefly dropping to 3.8% during the middle phase (1200–1300 ce), before surging to 22% in the final phase (1300–1450 ce). PH showed a steady upward trend, starting at 22% in the early phase, increasing to 25% in the middle phase, and peaking at 30% by the end of the LIP (McCool, Tung, et al. 2021). The absence of a significant relationship between these lesions and sex or age suggests that physiological stress was broadly distributed across the population, although lesion frequency does decrease with age (Data S1: Section 6).

## 2 Materials and Methods

In 2018 and 2019, W.C.M. excavated an MNI of 325 individuals from eleven chullpa tombs in the Nazca highlands (see demographic breakdown in SI and S2). Excavations and analyses were conducted under research and export permits issued by the Peruvian Ministry of Culture (No. 501–2018/DGPA/VMPCIC/MC; No. 036–2019‐VMPCIC‐MC). Following excavation and analysis, the human remains were curated at a secure Ministry of Culture facility in Peru.

Because the assemblage was disarticulated and commingled, crania were selected for dating, osteological analysis, and stable isotope chemistry. Of the 325 individuals, 267 crania were sufficiently complete for ante‐ and peri‐mortem trauma analysis, and 149 were directly dated (details and data summary in SI Appendix). Age‐at‐death was estimated using standard protocols for cranial suture closure, dental formation/eruption, and dental surface wear (Bass 1995; Buikstra and Ubelaker 1994). Individuals were assigned to six non‐overlapping age cohorts: infant (0–4 years), child (5–12), adolescent (13–20), young adult (21–35), middle adult (36–50), and old adult (51+) (White et al. 2011). Antemortem trauma was recorded through cranial surface examination for depressions and evidence of healing (Lovell 1997; Sauer 1998; Tung 2007). Perimortem fractures were identified by radiating fracture lines, color homogeneity between the cranial surface and fracture edges, and bone hinging at fracture margins (Ortner 2008; Sauer 1998; Tung 2007). Postmortem damage was distinguished by sharp fracture edges, color differences between the cranial surface and damaged area, and the absence of bone hinging or radiating fractures (Berryman and Haun 1996; Murphy et al. 2010; Ortner 2008; Sauer 1998; White et al. 2011). Because antemortem and perimortem trauma positively co‐vary, the present analysis focuses on peri‐mortem trauma (McCool, Tung, et al. 2021).

Porous lesions in the orbits (CO) and cranial vault (PH) were identified macroscopically and under low magnification (×10) following Standards (Buikstra and Ubelaker 1994). Lesions were recorded as present even when unilateral, with side noted in unilateral cases. Lesion expression was classified following Stuart‐Macadam (1985, 1991), and lesion activity at death was assessed (Mensforth et al. 1978). Vault lesions were further differentiated into true porosity and ectocranial pitting (Mann and Hunt 2013). Each cranium was photo‐documented to support independent validation of pathological findings. Prior research indicates that these lesion frequencies co‐vary with frailty and do not demonstrate problematic characteristics of the osteological paradox (McCool, Tung, et al. 2021). To minimize sampling bias, tombs with viable skeletal assemblages were randomly selected from different spatial contexts across the study region and excavated to sterile levels. These tombs were used over multiple centuries and contained a broad demographic range of age and sex profiles.

### 2.1 AMS Dating

To evaluate diachronic trends in violence, AMS radiocarbon dates were obtained for 149 crania excavated from tombs throughout the study region. Bone collagen for δ^14^C measurement was extracted and purified using the modified Longin method with ultrafiltration at Penn State University (PSU), the University of California Santa Barbara (UCSB), and the University of California Irvine, where AMS δ^14^C measurements were conducted (Kennett et al. 2017). Following Ambrose, only dates with C:N atomic ratios of 2.9–3.6 were included in the analysis (Ambrose 1990). Results were calibrated using the rcarbon package (Bevan and Crema 2020) in the R programming environment with the SHCal20 calibration curve (Hogg et al. 2020).

### 2.2 Stable Isotope Analyses

Samples were taken from the vomer, nasal concha, or nasal bones. Bone collagen for δ^13^C, δ^15^N, and δ^34^S analysis was extracted from 149 individuals using a modified Longin method (Kennett et al. 2017) following PSU Carbon Cycle AMS Facility protocols. Samples (400–700 mg) from the Nazca highlands and adjacent lowlands were cleaned, demineralized in 0.5 N HCl at 5°C for 24–36 h, and gelatinized in 0.01 N HCl at 70°C for 12 h. The gelatin solution was frozen, lyophilized, and ultrafiltered using precleaned Amicon Centriprep 30 ultrafilters. Filtered collagen was desalted, lyophilized, and weighed to determine percent yield. Ultrafilters were precleaned by sonication in 0.01 N HCl at ~60°C, followed by multiple rinses with ultrapure Millipore water. Bone standards were processed alongside unknown samples, and no modern or ancient carbon contamination was detected.

For bone carbonate analysis (Koch et al. 1997), samples were powdered with an agate mortar and pestle and treated with 2% bleach (NaOCl), using 0.04 mL NaOCl per milligram of bone powder. Samples were sonicated for 10 min, vortexed for 60 s, and left at room temperature for 24 h. Treated samples were rinsed with ultrapure Millipore water at PSU or double‐distilled deionized water at UCSB. Acetic acid pretreatment followed, with 0.04 mL of 0.1 M CH₃COOH added per milligram of bone powder. Samples were sonicated for 15 min, vortexed for 60 s, left at room temperature for 24 h, rinsed again, dried at 50.8°C for 24 h, and further dried overnight at 60°C. Although mineralogical analyses were not undertaken, the δ^13^C_apatite results are interpreted alongside the collagen‐isotope and archaeological evidence. Final isotopic analysis was conducted at the Center for Stable Isotopes, University of New Mexico, using continuous‐flow isotope ratio mass spectrometry with a GasBench device coupled to a Thermo Fisher Scientific Delta V Plus isotope ratio mass spectrometer. Results are reported in delta notation relative to VPDB. Reproducibility was better than 0.1‰ for δ^13^C based on repeated measurements of laboratory standard 123 Carrara Marble, calibrated against NBS‐19 with a δ^13^C value of 1.95‰. Isotope measurements and collagen preservation metrics (%C, %N, and atomic C:N ratios) are reported in Table S1.

### 2.3 Climate

The Quelccaya Ice Cap, located in the Cordillera Vilcanota in the high Andean Sierra, provides a high‐resolution paleoclimate record sensitive to the position of the Intertropical Convergence Zone and precipitation from the Amazon Basin (Thompson et al. 1985). Annual ice layers, extending back 1800 years, offer a detailed climate record for the Sierra. Proxies from ice‐accumulation rates and δ^18^O ratios indicate average precipitation, temperature, and rainfall variability. By using multiproxy records from lithic ratios in terrestrial sediment cores and pollen records from the Cerro Llamoca peatland in the Palpa highlands (approximately 50 km from the Nazca highlands), we reconstructed the local Nazca highland climate (Schittek et al. 2015). Lithic data rely on manganese (Mn) and iron (Fe) content, normalized to titanium (Ti), which is immobile in peat. Mn/Fe ratios serve as a proxy for moisture, with increasing Fe/Mn ratios indicating greater moisture. Poaceae pollen percentages also serve as a proxy for moisture, with higher percentages reflecting wetter conditions.

### 2.4 Quantitative Methods

We assessed regional, dietary, climatic, and demographic drivers of elevated δ^15^N values in the Nazca highlands LIP sample. Nazca individuals were compared with south‐central Peruvian populations from the Early Intermediate Period (EIP 200 bce–600 ce), MH, and LIP using linear models, Cohen's d, *t*‐tests, and Kruskal‐Wallis tests. Additional linear models evaluated relationships between δ^15^N, δ^34^S, δ^13^C_collagen_, and δ^13^C_apatite_ to assess whether dietary resources could explain nitrogen enrichment. LIP Andean δ^15^N values were also compared with paleoclimate proxies from the Quelccaya ice cap and Cerro Llamoca peatland core using time‐lagged analyses, Monte Carlo simulations, *t*‐tests, and GAMs. Within the Nazca highlands, δ^15^N variation was evaluated across three temporal phases within the LIP (1000–1200, 1200–1300, and 1300–1450 ce), and demographic models tested the effects of age and sex. All analyses were conducted in R, with additional details provided in the SI Appendix.

## 3 Results

To situate the Nazca highlands LIP δ^15^N dataset within the broader isotopic record of south‐central Peru, we compared it to a compiled dataset of human δ^15^N values from the Ica Valley, Nazca Valley, and Nazca Basin spanning the Early Intermediate Period (EIP), Middle Horizon (MH), and LIP (*n* = 373; Table 1; Data S1: Section 1 and S2). All comparative values and source references are provided in Data S1 and S2: Section 1. Differences among groups were evaluated using a linear model (δ^15^N~Phase), and estimated marginal means were compared using Tukey‐adjusted pairwise tests. The Nazca LIP sample has an estimated marginal δ^15^N mean of 13.36‰ (SE = 0.15), substantially higher than the comparative EIP (δ^15^N 9.19‰, SE = 0.11), MH (δ^15^N 9.00‰, SE = 0.12), and regional LIP (δ^15^N 9.87‰, SE = 0.23) groups. Pairwise comparisons indicate that the Nazca LIP differs significantly from all comparative phases (*p* < 0.0001), with offsets of approximately 3.5‰–4.4‰. Very large effect sizes further support this separation (Cohen's d = −3.41 for EIP, −4.38 for MH, and −3.83 for LIP). This distinction is particularly notable because the Nazca LIP values are elevated relative both to the preceding MH population from the Nazca basin and to other contemporaneous LIP populations from lower‐elevation Ica and Nazca contexts. Rather than falling at the upper end of a shared regional distribution, the Nazca LIP dataset is isotopically distinct from other south‐central Peruvian populations (Figure 2). The comparison does not identify the cause of that enrichment, but it establishes that the Nazca LIP values cannot be explained simply as continuity with the preceding local MH population or as a broader regional LIP pattern.

**TABLE 1:** Summary of statistical models for δ^15^N across analytical frameworks.

Model	Dataset/phase	Response	Predictor	Estimate/statistic	SE	t/F/MC	*p*	emmean
Regional analysis								
LM	EIP	δ^15^N	(Intercept)	9.1907	0.1112	82.623	< 0.001	9.19
LM	MH	δ^15^N	PhaseMH	−0.1897	0.1619	−1.172	0.242	9.00
LM	LIP	δ^15^N	PhaseLIP	0.6747	0.2572	2.623	0.009	9.87
LM	Nazca LIP	δ^15^N	PhaseN.LIP	4.1709	0.1513	27.571	< 0.001	13.36
Food resource								
GLM	Nazca LIP	δ^15^N	δ^13^Ccollagen	0.0152	0.0353	0.431	0.667	—
GLM	Nazca LIP	δ^15^N	δ^13^Capatite	0.0527	0.0382	1.379	0.170	—
GLM	Nazca LIP	δ^15^N	δ^34^S	−0.1105	0.1216	−0.909	0.371	—
Climate analysis								
Monte Carlo	Full Andes LIP	δ^15^N	Lagged δ^18^O	Mean *r* = 0.055	—	165.38	< 0.001	—
Monte Carlo	S. Highlands LIP	δ^15^N	Lagged δ^18^O	Mean *r* = 0.081	—	117.96	< 0.001	—
Pearson	Nazca LIP	δ^15^N	Lagged δ^18^O	*r* = −0.020	—	−0.241	0.810	—
Monte Carlo	Full Andes LIP	δ^15^N	Mn/Fe ratio	Mean *r* = 0.067	—	206.08	< 0.001	—
Monte Carlo	S. Highlands LIP	δ^15^N	Mn/Fe ratio	Mean *r* = 0.124	—	183.42	< 0.001	—
Pearson	Nazca LIP	δ^15^N	Mn/Fe ratio	*r* = −0.212	—	−1.042	0.308	—

*Note:* Estimate/Statistic: For LM and GLM models, values are β coefficients. For Monte Carlo correlations, values are mean *r* across permutations. For Pearson correlations, values are *r*. t/F/MC: For LM models, the test statistic is *t*. For GLM models, the test statistic is *t*. For Monte Carlo and Pearson correlations, the reported statistic is the Monte Carlo test statistic or *t*‐value respectively. emmean: Estimated marginal means at grand mean of covariates. Dashes (—) indicate models where emmeans were not computed. SE: Standard error of the estimate. Dashes indicate correlation analyses where SE is not applicable.

Abbreviations: EIP = early intermediate period; GLM = generalized linear model; LIP = late intermediate period; LM = linear model; MH = middle horizon.

**FIGURE 2:**
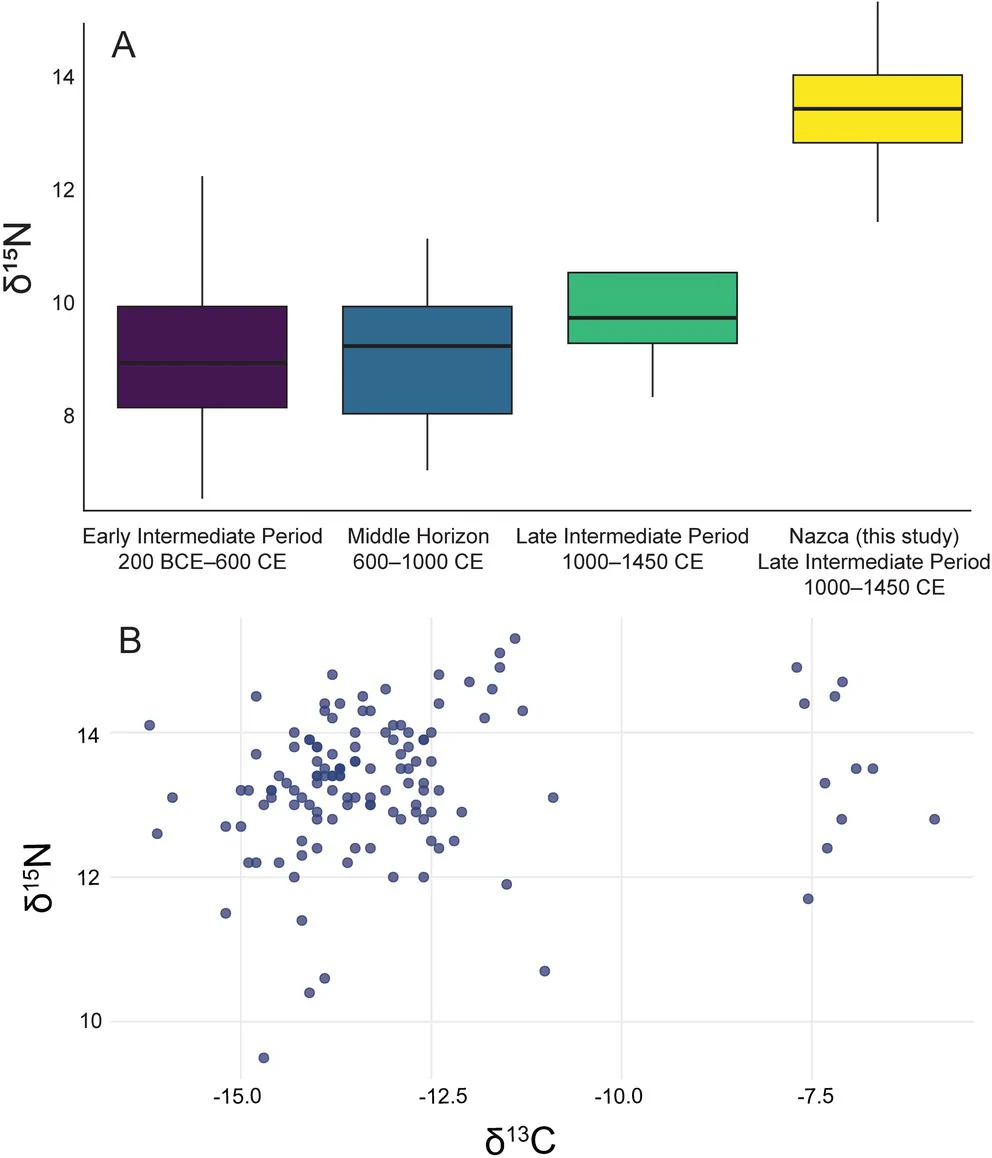
Comparison of human stable‐isotope values among the study populations. (A) Bone‐collagen δ15N values from Early Intermediate Period (200 bce–600 ce), Middle Horizon (600–1000 ce), regional Late Intermediate Period (1000–1450 ce), and Nazca Late Intermediate Period (1000–1450 ce) samples. Nazca LIP individuals exhibit substantially higher δ15N values than the comparative regional groups. (B) Relationship between bone‐apatite δ13C and bone‐collagen δ15N values among Nazca LIP individuals.

To further contextualize the elevated values, we compared LIP human δ^15^N against modern camelid and *cuy* (Guinea Pig) references collected directly from study sites in the Nazca region. While the sample size of this baseline is limited, and a more robust dietary comparison is developed in the following sections, LIP Nazca humans have significantly higher δ^15^N values relative to both camelid (*p* = 0.006, Cohen's d = −1.92) and cuy (*p* = 0.002, Cohen's d = −5.20) baselines, with differences approaching the expected enrichment for a full trophic step (Kruskal‐Wallis *χ* ^2^ = 20.70, df = 2, *p* < 0.0001). These modern faunal references do not fully characterize the archaeological Nazca food web, particularly in the absence of archaeological plant, faunal, and soil isotope baselines. We consider this limitation and the potential contribution of dietary and baseline effects in greater detail below.

### 3.1 Driver 1: Climate Change

To determine whether climate change was the primary driver of elevated δ^15^N values in the Nazca highlands, we analyzed δ^15^N values from two comparative datasets: one comprising all LIP individuals across the Andes (*n* = 841) and another comprising LIP individuals from the South‐Central Peruvian highlands spanning the Ica and Ayacucho regions (*n* = 159). We compared these values with two climate proxies: a 10‐year lagged supraregional δ^18^O precipitation proxy from the Quelccaya ice cap and a 10‐year lagged local pollen precipitation proxy from the Cerro Llamoca peatland core (Figure 3; Table 1; Data S1: Section 4) (Schittek et al. 2015; Thompson et al. 1985). Previous research has identified severe droughts during the LIP, when reduced precipitation and increased evaporation may have concentrated nitrogen in soils and water, elevating δ^15^N values in plants and throughout the food web (Craine et al. 2009; Murphy and Bowman 2009). If climate change was the primary driver of elevated δ^15^N values, we expected δ^15^N to correlate positively with proxies indicating drought.

**FIGURE 3:**
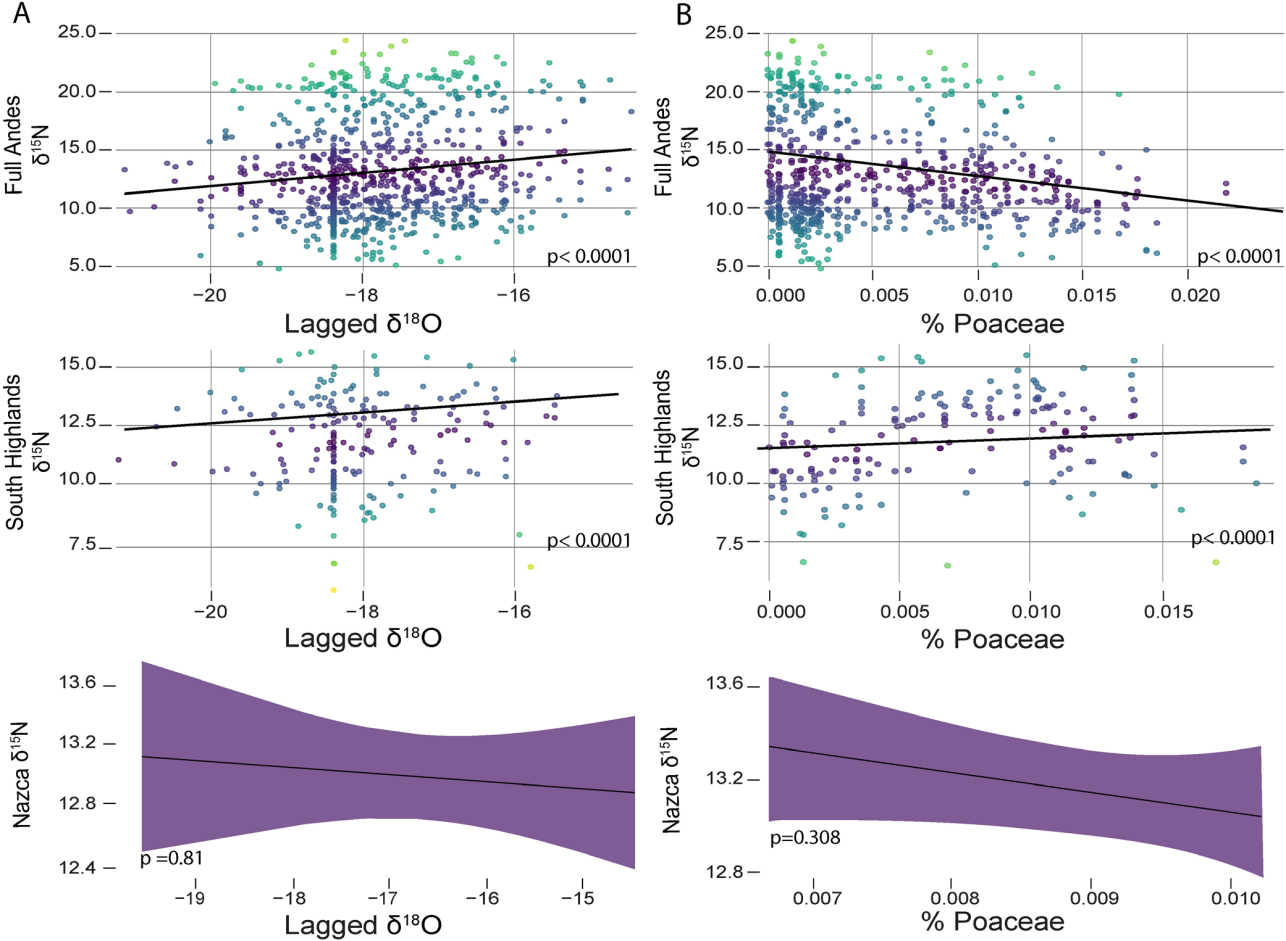
(A) Human δ^15^N plotted against lagged δ^18^O and (B) percentage of Poaceae. From top to bottom, plots show the full Andean comparative dataset, the southern highland subset, and the Nazca study population. Points represent individuals, black lines show fitted trends, and purple shading indicates 95% confidence intervals. Environmental proxies were significantly associated with δ^15^N in the comparative datasets (*p* < 0.0001), but not within the Nazca population (lagged δ^18^O: *p* = 0.81; Poaceae: *p* = 0.308).

A Monte Carlo simulation of the full LIP Andean dataset showed that 94.94% of correlations between δ^15^N values and the δ^18^O precipitation proxy were positive, with a *t*‐value of 165.38 (*p* < 0.0001), indicating a significant positive relationship. In the LIP South Highlands subset, 88.83% of correlations were positive, with a t‐value of 117.96 (*p* < 0.0001), also indicating a significant positive relationship (Data S1: Sections 4.1–4.3). However, within the Nazca highlands, the Pearson correlation test found no significant relationship between δ^15^N values and the δ^18^O proxy (coefficient = −0.0201, *p* = 0.8099), and the Generalized Additive Model (GAM) identified no nonlinear relationship (*p* = 0.81).

We also assessed the relationship between δ^15^N and the 10‐year lagged local pollen precipitation proxy from the Cerro Llamoca core. This lag accounts for the time required for changes in precipitation to influence soil nutrients, plant isotope values, and the diets of humans and animals (Figure 3; Table 1; Data S1: Sections 4.4–4.6). A Monte Carlo simulation of the full LIP Andean dataset showed that 97.82% of correlations were positive, with a *t*‐value of 206.08 (*p* < 0.0001). In the LIP South Highlands subset, 96.44% of correlations were positive, and the *t*‐value of 183.42 indicated a significant positive relationship (*p* < 0.0001). In contrast, the Nazca highlands dataset showed no significant relationship in either the Pearson correlation test (coefficient = −0.2124, *p* = 0.3081) or the GAM analysis (*p* = 0.308).

These regional‐scale comparisons demonstrate that human δ^15^N values broadly covaried with climate across both the full LIP Andean dataset and the South Highlands subset. However, neither climate proxy was significantly associated with δ^15^N within the Nazca highlands. Thus, although climate contributed to broader regional variation in human δ^15^N, it does not explain the consistently elevated values observed in the Nazca population.

### 3.2 Driver 2: Temporal and Demographic Impacts

An analysis of potential temporal and demographic drivers behind the observed enrichment in δ^15^N values was conducted. We assessed whether elevated δ^15^N values were concentrated in specific temporal or demographic subsets of our dataset (Data S1: Section 5).

To assess the temporal consistency of δ^15^N isotope values, the radiocarbon‐dated Nazca highland crania sample (*n* = 149) was divided into three phases: Phase I (1000–1200) marked the transition into the LIP, Phase II (1200–1300 ce) represented the middle LIP, and Phase III (1300–1450 ce) marked the final phase (McCool, Anderson, and Kennett 2021). Our linear model revealed no significant differences in δ^15^N values between the phases, indicating consistently elevated values throughout the LIP. A Kruskal‐Wallis test supported this, showing no significant difference across phases (*χ* ^2^ = 2.1277, df = 2, *p* = 0.3451). The differences in δ^15^N values between phases were less than a trophic level and not statistically significant (Table 1). These results suggest the elevated δ^15^N values were sustained over time and reflected a persistent characteristic of the Nazca population during the LIP.

Demographic analyses confirmed no relationship between δ^15^N values and age or sex. The linear model showed no significant difference between adults (mean 13.4‰) and children (mean 13.3‰), or between males (mean δ^15^N of 13.6‰) and females (mean δ^15^N of 13.2‰), with all variations within the expected range for a standardized diet (Table 1). This stability across all LIP phases reinforced the conclusion that elevated nitrogen isotope levels were consistent across the population throughout the LIP.

### 3.3 Driver 3: Dietary Inputs and Local Baseline Effects

We evaluated whether dietary inputs could account for the elevated δ^15^N values observed in the Nazca Highlands population (Data S1: Section 2). Agricultural fertilization represents one possible source of nitrogen enrichment, particularly if crops were fertilized with camelid dung or seabird guano. Carbon isotope values can be used to evaluate whether elevated δ^15^N values are associated with increased consumption of maize or other C₄ foods. Carbon isotope results do not indicate that elevated δ^15^N values were associated with systematic differences in C₃ versus C₄ dietary inputs. δ^13^Capatite, which reflects whole‐diet carbon inputs, was not significantly associated with δ^15^N (*p* = 0.170). δ^13^Ccollagen, which more strongly reflects dietary protein, was also not significantly associated with δ^15^N (*p* = 0.667) (Table 1; Figure 4C; Data S1: Section 2). We found no significant association between higher δ^15^N values and a systematic shift in C₃ versus C₄ dietary inputs. However, there is the possibility that fertilization could elevate crop δ^15^N independently of δ^13^C, and therefore fertilization cannot be excluded on the basis of carbon isotope values alone.

Marine resource consumption was also evaluated as a possible dietary source of elevated δ^15^N. Nazca highland human δ^15^N values overlap with marine fish values in the compiled food baseline dataset, indicating that marine foods are an isotopic match based on nitrogen alone (Data S1: Section 2; Figure 4A). However, δ^15^N overlap is not diagnostic because high nitrogen values can also result from fertilized crops, terrestrial animal protein, freshwater resources, local environmental baselines, or physiological stress. Sulfur isotope values for Nazca humans do not support substantial marine fish consumption as the primary explanation for the elevated δ^15^N values. If marine fish were the main driver, individuals with higher δ^15^N values would be expected to have more marine‐like δ^34^S values. Instead, δ^15^N was not significantly associated with δ^34^S (*p* = 0.371; Table 1; Figure 4B–C; Data S1: Section 2). While sulfur values can also reflect geology, soils, hydrology, and local baseline variation, the available δ^34^S data do not support marine resources as the primary source of nitrogen enrichment.

Bayesian mixing models further indicate that dietary estimates are highly sensitive to isotope selection and baseline construction (Figure 4D). Models using only δ^13^C and δ^15^N estimated high marine fish contributions, whereas models that incorporated δ^34^S for the subset of Nazca individuals resulted in sharply reduced estimated marine input and shifted the elevated nitrogen signal toward terrestrial high‐δ^15^N sources. Given these conflicting outputs, these model differences should not be interpreted as literal dietary reconstructions. Instead, they indicate that δ^13^C–δ^15^N models may overestimate marine consumption when enriched δ^15^N values are present.

**FIGURE 4:**
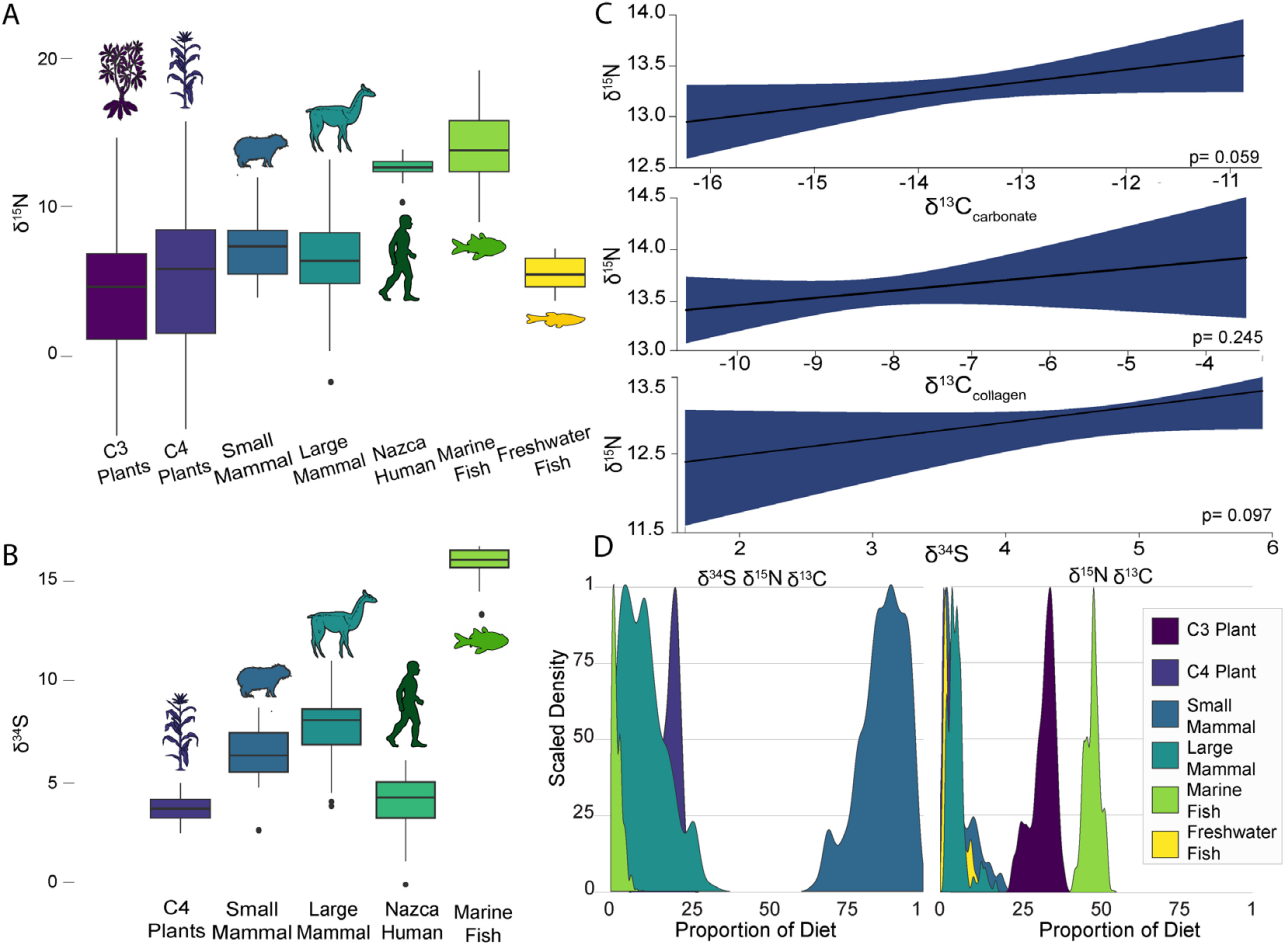
(A) Boxplot comparing δ^15^N values across dietary baselines, including C3 and C4 plants, small and large terrestrial mammals, marine and freshwater fish, and Nazca highland human samples (this study). (B) Boxplot comparing δ^34^S values for small and large terrestrial mammals, marine fish, and Nazca humans. (C) Linear models assessing the relationship between δ^15^N and δ^13^C (collagen and apatite) and δ^34^S. (D) Bayesian mixing models indicating dietary contributions for δ^13^C, δ^15^N, and δ^34^S.

## 4 Discussion

Persistent conflict during the Late Intermediate Period (LIP: 1000–1450 ce) fundamentally altered subsistence conditions in the Nazca highlands, restricting mobility and access to resources while placing increasing pressure on food production and security (Kuzminsky et al. 2024). Carbon and sulfur isotope values interpreted here indicate a strongly C4‐oriented terrestrial diet, with little evidence that substantial marine protein consumption accounts for the consistently elevated δ^15^N values. Instead, the elevated δ^15^N values stand in marked contrast to both the preceding Middle Horizon population from the same region and contemporaneous populations across the southern highlands, where values are lower and evidence for sustained warfare is less pronounced (Kellner and Schoeninger 2008) mean N = ~8‰; (Webb et al. 2013 mean N = ~9‰). Although individual values equal to or higher than those observed here have been reported elsewhere in the Andes (See Data S2), the Nazca highland sample is distinctive because elevated values occur consistently across the population throughout the LIP. Here we argue that the elevated δ^15^N values could reasonably result from increased fertilization of more intensively cultivated land but may also reflect periods of chronic undernutrition. Under conditions of environmental instability and heightened conflict, communities faced greater pressure to maximize production from the resources that remained accessible to them and greater difficulty in buffering periods of reduced food availability. To fully contextualize these values within the Nazca highland population, it is necessary to discuss the specific environmental and social stressors of this period.

Agricultural production within the Andean highlands, ranging from approximately 2100–4300 m in elevation, posed major challenges because unpredictable agroecological conditions produced highly variable food yields (Browman 1987; Goland 1993; Kuznar 2001; Winterhalder and Thomas 1978). Slope, soil type, moisture, and solar radiation shaped the reliability of food production, requiring highland communities to reduce subsistence risk through economic diversification, mobility, and long‐distance travel (Browman 1987; Kuznar 2001; Winterhalder and Thomas 1978). Human habitation was often concentrated in limited arable patches, while camelid herding depended on movement to high‐altitude pastures (Murra 1972; Nielsen 2009). These diversified agropastoral systems supported the expansive Wari and Tiwanaku highland settlements during the MH and remained the foundation of highland subsistence into the LIP, but their effectiveness depended on continued access to agricultural land, pasture, and exchange networks.

The collapse of Wari and Tiwanaku marked the beginning of a LIP characterized in many regions by disrupted food production and redistribution, increased violence, and social instability (Arkush 2010; Bauer and Kellett 2010; Ibarra Asencios 2021; Kellett 2010; Washburn et al. 2022). Highland populations had previously relied on extensive networks for redistributing agricultural products, but state collapse fragmented these systems, isolating communities and restricting access to diverse resources (Edwards 2021; Smith et al. 2021). In the Nazca highlands, this instability produced major shifts in settlement patterns with LIP residential sites positioned on difficult‐to‐access hilltops and ridgelines and fortified against raids (McCool, Anderson, and Kennett 2021; McCool et al. 2022, 2024). These settlement patterns reflect and reinforce restricted movement across the landscape and increased vulnerability to disruptions in food production and access. Despite these measures, many individuals experienced violent deaths (McCool et al. 2024, 2022; McCool, Anderson, and Kennett 2021).

The restrictions on mobility would have altered how Nazca highland communities managed the inherent variability of highland food production. During the Middle Horizon, access to dispersed agricultural land, pasture, and exchange networks allowed communities to buffer shortfalls by drawing on resources from different parts of the landscape (Browman 1987; Conlee 2021). For populations concentrated in fortified LIP settlements, these established strategies would have become increasingly difficult to maintain, increasing dependence on the agricultural land and resources in their immediate vicinity (Mantha 2009; Washburn et al. 2022). Communities may therefore have cultivated nearby fields more intensively and applied camelid dung to maintain yields from a smaller and more heavily used agricultural area. Repeated manuring would have increased crop δ^15^N values, which would subsequently have been transmitted to human consumers. Experimental studies demonstrate this effect, and dung fertilization has been proposed to explain elevated human δ^15^N elsewhere in the Andes (Finucane et al. 2006; Szpak 2014; Szpak et al. 2012; Szpak and Chiou 2020). The distinctive feature of the Nazca highland sample is not the magnitude of its highest δ^15^N values, but the population‐wide consistency of the enrichment. This pattern contrasts with the substantially lower δ^15^N values documented among nearby populations with less evidence of warfare, supporting an association between elevated δ^15^N and the changes in mobility and food production accompanying conflict and settlement fortification. Additional archaeological plant, faunal, and soil isotope data from the Nazca highlands are needed to determine how much dung fertilization contributed to this pattern.

The same restrictions that encouraged agricultural intensification would also have increased the risk of food insecurity by limiting the resources available when local production declined. Poor harvests or seasonal shortfalls that might previously have been offset through access to dispersed fields, pastures, or exchange partners were therefore more likely to result in periods of insufficient food availability. The high prevalence of CO and PH provides independent evidence of a substantial burden of physiological stress among the Nazca highland population (McCool, Tung, et al. 2021; McCool et al. 2024). Neither lesion varied significantly by age or sex, providing no evidence that their distribution was concentrated within a particular demographic group. Although CO and PH are nonspecific and cannot identify the nutritional or pathological conditions responsible for their formation, their substantially higher prevalence relative to the preceding Middle Horizon is consistent with an increased burden of physiological stress during the LIP. These lesions cannot establish that food insecurity was their cause, but their population‐wide distribution accords with other evidence for worsening conditions amid escalating violence, population pressure, and constraints on mobility and resource access.

If nutritional stress contributed to the elevated δ^15^N values, the slow remodeling of bone collagen indicates that it more likely reflected recurrent seasonal or prolonged food insecurity than a single episode of acute starvation. Periods of insufficient energy intake can induce catabolism, during which the body mobilizes endogenous proteins and becomes enriched in ^15^N (Haubert et al. 2005; Hobson et al. 1993; Robertson et al. 2014). Recurrent or prolonged undernutrition may influence bone‐collagen δ^15^N, although tissue turnover and temporal averaging limit how directly this signal can be linked to specific episodes of nutritional stress. In contrast, acute starvation ends before sufficient remodeling has occurred to produce detectable enrichment in bone, although short‐term nutritional changes have been identified through serial analysis of rapidly forming tissues such as hair (Mekota et al. 2006; Neuberger et al. 2013). Previous nutritional studies have documented δ^15^N enrichment during fasting or severe undernutrition (Beaumont and Montgomery 2016; Mekota et al. 2006). Therefore, recurrent nutritional stress may have augmented a dietary signal already elevated through the consumption of dung‐fertilized crops. The population‐wide enrichment may thus reflect the combined effects of changes in food production and recurrent seasonal food insecurity rather than either process alone. A similar combination of fertilizer use and reduced food availability has been proposed for the nearby Ayacucho Basin, where Kuzminsky et al. (2024) documented elevated post‐Wari δ^15^N values during a period of drought and social disruption.

These findings suggest that both crop fertilization and undernutrition‐associated catabolism are plausible, potentially interacting explanations for the elevated δ^15^N values. However, as with any isotopic study, additional data may refine this interpretation. The absence of excavated local baselines for fauna, flora, and soils in the Nazca highlands limits how precisely environmental, dietary, and physiological factors can be separated. Some uncertainty in collagen turnover rates for the sampled bones also limits the temporal resolution of these interpretations. Future research that develops these baselines and applies incremental tissue analyses, such as serial dentin or hair sampling, would allow nutritional stress to be evaluated in greater detail through time. The elevated δ^15^N values in the Nazca highland population raise questions about how agricultural practices and nutritional stress shaped human diets and health during a period of prolonged conflict and environmental change.

## 5 Conclusion

The LIP Nazca highland population consumed a predominantly terrestrial, strongly C4‐oriented diet within a ^15^N‐enriched subsistence system. Low δ^34^S values provide little support for substantial marine protein consumption, leaving the elevated δ^15^N values to be understood primarily within changes to terrestrial food production and physiology. Greater use of camelid dung as fertilizer could have increased the δ^15^N of cultivated foods and contributed substantially to the approximately 3.5‰–4.4‰ difference between the Nazca highland population and the comparative populations. However, the available local baseline data do not allow the contribution of fertilization to be quantified precisely.

The broader archaeological and osteological context provides an explanation for why these changes occurred. Persistent warfare restricted mobility and access to the dispersed agricultural land, pasture, and exchange networks that had long allowed highland communities to buffer variability in food production. Greater dependence on accessible agricultural land would have encouraged intensification, including increased fertilizer use, while simultaneously increasing vulnerability to recurrent seasonal or periodic food shortages. Such shortages could have produced repeated episodes of catabolism. The similarly high post‐Wari δ^15^N values reported from the nearby Ayacucho Basin, where increased fertilization and reduced food availability have also been proposed, further support this interpretation.

The consistently elevated δ^15^N values in the Nazca highlands cannot be attributed to a single process. Instead, they reflect the broader reorganization of subsistence that accompanied persistent conflict during the LIP. Taken together, the isotopic, archaeological, and osteological evidence documents a population adapting to increasingly restricted access to land and resources while facing greater difficulty maintaining food security.

## Author Contributions

**Sarah J. Noe:** conceptualization, writing – original draft, methodology, visualization, writing – review and editing, formal analysis, data curation. **Bridgette Degnan:** conceptualization, writing – original draft, writing – review and editing, formal analysis. **Emily C. Kracht:** conceptualization, writing – original draft, writing – review and editing, formal analysis. **Weston C. McCool:** conceptualization, investigation, funding acquisition, writing – original draft, methodology, writing – review and editing, formal analysis, project administration, data curation, supervision, resources.

## Funding

This research was supported by the National Science Foundation Archaeology Doctoral Dissertation Research Improvement Grant No. 1934521.

## Ethics Statement

Permission to conduct the research and export the material was granted by the Peruvian Ministry of Culture under Permit No. 501–2018/DGPA/VMPCIC/MC and No. 036–2019‐VMPCIC‐MC. No institutional ethics board approval was required because this research did not involve living human subjects.

## Conflicts of Interest

The authors declare no conflicts of interest.

## Supporting information


**Data S1:** Skeletal lesions by period and sex (McCool et al. 2021:820).

**Data S2:** ajpa70373‐sup‐0002‐Supinfo.pdf.

**Data S3:** ajpa70373‐sup‐0003‐Supinfo.pdf.

## Data Availability

All data supporting this study are included in the article, the Supporting Information, and the accompanying Dryad repository: https://doi.org/10.5061/dryad.h44j0zq30. The repository contains the osteological, stable‐isotope, comparative, and environmental datasets used in the analyses. All study data are included in the article and/or Supporting Information and Data S2 Appendix. Additional details on the osteological dataset are derived from published sources and are also available from the corresponding author upon request.
