# Supplementary material for: Leveraging Multi‐Isotope Systems to Investigate Conflict, Food Insecurity, and Elevated Nitrogen Isotope Values in the Nazca Highlands of Peru During the Late Intermediate Period (1000–1450 CE)
Source: Am J Biol Anthropol. 2026 Oct 30;191(3):e70373. doi: 10.1002/ajpa.70373 (PMC13722818; doi:10.1002/ajpa.70373)
Supplement: Supplementary file 1 [file AJPA-191-e70373-s001.pdf]

Bayesian Mixing Model

```
#### Carbon and Nitrogen ####
library(ggplot2)
library(MixSIAR)
library(R2jags) # Used to extract mixing model posterior density data for custom plotting

# Import human consumer data.
mix.filename <- "PeruLIP.csv"
read.csv(mix.filename)
mix <- load_mix_data(filename = mix.filename,
                     iso_names = c("d13CC", "d15N"),
                     factors = c("Nazca"),
                     fac_random = c(FALSE),
                     fac_nested = c(FALSE),
                     cont_effects = NULL)

source.raw <- read.csv("Feeding.csv")
head(source.raw)
ss.m <- aggregate(cbind(source.raw$d13CC, source.raw$d15N) ~ source.raw$type, FUN = mean)
ss.v <- aggregate(cbind(source.raw$d13CC, source.raw$d15N) ~ source.raw$type, FUN = sd)
ss.c <- aggregate(source.raw$type, by = list(source.raw$type), FUN = length)

source.summary <- data.frame(resource = ss.m[, 1], Meand13CC = ss.m[, 2], SDd13CC = ss.v[,
2], Meand15N = ss.m[, 3], SDd15N = ss.v[, 3], n = ss.c$x)
write.csv(source.summary, "sources.csv", row.names = F)

source.filename <- "sources.csv"
read.csv(source.filename)
source <- load_source_data(filename = source.filename, source_factors = NULL, conc_dep =
FALSE, data_type = "means", mix)

dn <- 3:6 # Trophic enrichment factors (discrimination) for nitrogen
dc <- seq(4.5, 6, by = .5) # Trophic enrichment factors (discrimination) for carbon
tef <- expand.grid(Meand13CC = dc, SDd13CC = 0.63, Meand15N = dn, SDd15N = 0.74) # SDs
based on literature

# Check whether the user's priors are non-informative or if they inadvertently introduce a
bias
plot_prior(alpha.prior = 1, source)

# Write the JAGS model file
model_filename <- "MixSIAR_model.txt" # Name of the JAGS model file
resid_err <- TRUE
process_err <- TRUE
write_JAGS_model(model_filename, resid_err, process_err, mix, source)

discr <- data.frame(
  resource = c("C3 plant", "C4 plant", "freshwater fish", "large terrestrial mammal",
"small terrestrial mammal", "marine fish"),
  Meand13CC = rep(tef[1, 1], 6),
  SDd13CC = rep(tef[1, 2], 6),
  Meand15N = rep(tef[1, 3], 6),
  SDd15N = rep(tef[1, 4], 6)
)

# Specify discrimination data for testing purposes
write.csv(discr, "discrimination.csv", row.names = F) # Export discrimination table to a
csv file
d <- load_discr_data("discrimination.csv", mix) # Import discrimination file

# Define model structure
model_filename <- "MixSIAR_model.txt"
resid_err <- TRUE
process_err <- TRUE
```

```
# Write the JAGS model file
write_JAGS_model(model_filename, resid_err, process_err, mix, source)

# Output options
output_options <- list(
  summary_save = TRUE,
  summary_name = "summary_statistics",
  sup_post = FALSE,
  plot_post_save_pdf = TRUE,
  plot_post_name = "posterior_density",
  sup_pairs = FALSE,
  plot_pairs_save_pdf = TRUE,
  plot_pairs_name = "pairs_plot",
  sup_xy = TRUE,
  plot_xy_save_pdf = FALSE,
  plot_xy_name = "xy_plot",
  gelman = TRUE,
  heidel = FALSE,
  geweke = TRUE,
  diag_save = TRUE,
  diag_name = "diagnostics",
  indiv_effect = FALSE,
  plot_post_save_png = TRUE,
  plot_pairs_save_png = FALSE,
  plot_xy_save_png = FALSE,
  diag_save_ggmcmc = FALSE,
  return_obj = TRUE
)

# Run the model and store results
results_list <- list()

for (i in 1:15) {
  cat("Running model iteration", i, "\n")
  jags.test <- run_model(run = "long", mix, source, d, model_filename)
  output <- output_JAGS(jags.test, mix, source, output_options)

  # Extract the dietary contributions
  dietary_contributions <- output$summary_stats[grep("p.resource",
rownames(output$summary_stats)), ]
  results_list[[i]] <- dietary_contributions
  print(dietary_contributions)
}
```

```
#### Carbon Nitrogen Sulfur ####

library(ggplot2)
library(MixSIAR)
library(R2jags) # Used to extract mixing model posterior density data for custom plotting

# Import human consumer data.
mix_data <- read.csv("Peru.csv")
filtered_mix_data <- subset(mix_data, !is.na(d34S))
write.csv(filtered_mix_data, "Filtered_Peru.csv", row.names = FALSE)
mix <- load_mix_data(filename = "Filtered_Peru.csv",
```

```
                     iso_names = c("d13CC", "d15N", "d34S"),
                     factors = c("Nazca"),
                     fac_random = FALSE,
                     fac_nested = FALSE,
                     cont_effects = NULL)

source.raw <- read.csv("Feeding.csv")
source.raw.34 <- subset(source.raw, !is.na(d34S))
head(source.raw)
ss.m <- aggregate(cbind(source.raw.34$d13CC, source.raw.34$d15N, source.raw.34$d34S) ~
source.raw.34$type, FUN = mean)
ss.v <- aggregate(cbind(source.raw.34$d13CC, source.raw.34$d15N, source.raw.34$d34S) ~
source.raw.34$type, FUN = sd)
ss.c <- aggregate(source.raw.34$type, by = list(source.raw.34$type), FUN = length)

source.summary <- data.frame(resource = ss.m[, 1], Meand13CC = ss.m[, 2], SDd13CC = ss.v[,
2], Meand15N = ss.m[, 3], SDd15N = ss.v[, 3], Meand34S = ss.m[, 4], SDd34S = ss.v[, 4], n
= ss.c$x)
write.csv(source.summary, "sources.csv", row.names = F)

source.filename <- "sources.csv"
read.csv(source.filename)
source <- load_source_data(filename = source.filename, source_factors = NULL, conc_dep =
FALSE, data_type = "means", mix)

dn <- 3:6 # Trophic enrichment factors (discrimination) for nitrogen
dc <- seq(4.5, 6, by = .5) # Trophic enrichment factors (discrimination) for carbon
ds <- seq(0, 3, by = 1) # Trophic enrichment factors (discrimination) for sulfur
tef <- expand.grid(Meand13CC = dc, SDd13CC = 0.63, Meand15N = dn, SDd15N = 0.74, Meand34S
= ds, SDd34S = 1.0 ) # SDs based on literature

# Plot prior (1 = uninformative)
plot_prior(alpha.prior = 1, source)

# Write the JAGS model file
model_filename <- "MixSIAR_model.txt" # Name of the JAGS model file
resid_err <- TRUE
process_err <- TRUE
write_JAGS_model(model_filename, resid_err, process_err, mix, source)

discr <- data.frame(
  resource = c("large terrestrial mammal", "small terrestrial mammal", "marine fish"),
  Meand13CC = rep(tef[1, 1], 3),
  SDd13CC = rep(tef[1, 2], 3),
  Meand15N = rep(tef[1, 3], 3),
  SDd15N = rep(tef[1, 4], 3),
  Meand34S = rep(tef[1, 5], 3),
  SDd34S = rep(tef[1, 6], 3)
) # Specify discrimination data for testing purpose
write.csv(discr, "discrimination.csv", row.names = F) # Unfortunately, MixSIAR requires
importation of a csv discrimination file. Therefore, export discrimination table to a csv
file to be imported next step.
d <- load_discr_data("discrimination.csv", mix) # Import discrimination file.

# Define model structure
model_filename <- "MixSIAR_model.txt"
resid_err <- TRUE
process_err <- TRUE

# Write the JAGS model file
write_JAGS_model(model_filename, resid_err, process_err, mix, source)

output_options <- list(
  summary_save = TRUE,
  summary_name = "summary_statistics",
```

```
  sup_post = FALSE,
  plot_post_save_pdf = TRUE,
  plot_post_name = "posterior_density",
  sup_pairs = FALSE,
  plot_pairs_save_pdf = TRUE,
  plot_pairs_name = "pairs_plot",
  sup_xy = TRUE,
  plot_xy_save_pdf = FALSE,
  plot_xy_name = "xy_plot",
  gelman = TRUE,
  heidel = FALSE,
  geweke = TRUE,
  diag_save = TRUE,
  diag_name = "diagnostics",
  indiv_effect = FALSE,
  plot_post_save_png = TRUE,
  plot_pairs_save_png = FALSE,
  plot_xy_save_png = FALSE,
  diag_save_ggmcmc = FALSE,
  return_obj = TRUE
)

# Run the model and print results
results_list <- list()

for (i in 1:15) {
  cat("Running model iteration", i, "\n")
  jags.test <- run_model(run = "test", mix, source, d, model_filename,
                         alpha.prior = 1, resid_err = resid_err,
                         process_err = process_err)
  output <- output_JAGS(jags.test, mix, source, output_options)

  # Extract the dietary contributions
  dietary_contributions <- output$summary_stats[grep("p.Nazca.",
rownames(output$summary_stats)), ]
  results_list[[i]] <- dietary_contributions
  print(dietary_contributions)
}
```
