# Supplementary material for: Leveraging Multi‐Isotope Systems to Investigate Conflict, Food Insecurity, and Elevated Nitrogen Isotope Values in the Nazca Highlands of Peru During the Late Intermediate Period (1000–1450 CE)
Source: Am J Biol Anthropol. 2026 Oct 30;191(3):e70373. doi: 10.1002/ajpa.70373 (PMC13722818; doi:10.1002/ajpa.70373)
Supplement: Supplementary file 2 [file AJPA-191-e70373-s002.pdf]

AJBA S1

Contents

0.1 Results . . . 1
0.2 Library . . . 1
0.3 Section 1. Local Comparison: d15N Analysis in SouthHighlands . . . 3
0.4 Section 2.1: 15N and 13C . . . 7
0.5 Section 2.2: 15N v 34S . . . 10
0.6 Section 2.3: 15N v Food resources . . . 12
0.7 Section 4: Influence of climate . . . 20
0.8 Section 4.1: d15N-quelccaya_d18O Full Andes Analysis . . . 20
0.9 Section 4.2: d15N-quelccaya_d18O South Highlands Analysis . . . 24
0.10 Section 4.3: d15N-quelccaya_d18O Nazca Dataset . . . 27
0.11 Section 4.4: Cerro Llamoca core Full Andes Analysis . . . 30
0.12 Section 4.5: Cerro Llamoca coreSouth Highlands Subset Analysis . . . 34
0.13 Section 4.6: Cerro Llamoca core Nazca Analysis . . . 38
0.14 Section 5: Change in dN15 through time . . . 41
0.15 Section 6: Violence and Physical Stress through time . . . 47
0.16 Section 7: Starvation Overview . . . 49

This is Supplemental 1, supplemental analysis code for the manuscript "Identifying a Starvation Signature in the Nazca Highlands of Peru During the Late Intermediate Period (1000 - 1450 CE)" submitted to American Journal of Biological Anthropology (AJBA) and written by Noe, Sarah J., Kracht, Emily C., Degnan, Bridgette, and McCool, Weston C. For questions about the code, please contact Sarah J. Noe at snoe@ucsb.edu

0.1 Results

0.1.1 Working Directory

0.2 Library

```r
setwd("/Users/72sno/Documents/02_Sarah/Nazca_Human_Isotope/Code")


library(emmeans)
```

```
## Welcome to emmeans.
## Caution: You lose important information if you filter this package's results.
## See '? untidy'
```

```r
library(stringr)
library(zoo)
```

```
##
## Attaching package: 'zoo'

## The following objects are masked from 'package:base':
##
##     as.Date, as.Date.numeric
```

```r
library(ggplot2)
```

```
## Warning: package 'ggplot2' was built under R version 4.4.3
```

```r
library(visreg)
```

```
## Warning: package 'visreg' was built under R version 4.4.3
```

```r
library(mgcv)
```

```
## Loading required package: nlme

## This is mgcv 1.9-1. For overview type 'help("mgcv-package")'.
```

```r
library(viridis)
```

```
## Loading required package: viridisLite
```

```r
library(dplyr)
```

```
## Warning: package 'dplyr' was built under R version 4.4.3

##
## Attaching package: 'dplyr'

## The following object is masked from 'package:nlme':
##
##     collapse

## The following objects are masked from 'package:stats':
##
##     filter, lag

## The following objects are masked from 'package:base':
##
##     intersect, setdiff, setequal, union
```

```
library(tidyr)
library(effsize)

#Nazca study Dataset
Nazca.data <- read.csv("Nazca.dataset.csv")
Nazca.LIP <- subset(Nazca.data, Phase2 == "LIP")

#South Highlands Reference Dataset
SouthHighlands <- read.csv("SouthHighlands.csv")
Feeding.Baseline <- read.csv("Food.csv")
Feeding.Baseline.local <- subset(Feeding.Baseline, local == "Local")
#LIP Isotope Dataset with Year ranges for Monte Carlo Simulation
Human.LIP <- read.csv("LIP.Isotope.Refernce.Climate.csv")
#Ice Core Dataset (supraregional)
climate.d18O <- read.csv("Climate.quelccaya.d18O.csv")
# Pollen Dataset (Regional)
climate.pollen <- read.csv("Climate.Pollen.Cerro.Llamoca.peatland.csv")
#Comparison of d15N values from Starvation case studies
Compiledstarvation <- read.csv("Starvation.Comparison.csv")
```

0.3 Section 1. Local Comparison: d15N Analysis in SouthHighlands

We investigated 15N values across different archaeological phases in the Nazca Highlands, spanning the Early Intermediate Period (EIP), Middle Horizon (MH), and Late Intermediate Period (LIP).

```
SouthHighlands$Phase <- factor(
  SouthHighlands$Phase,
  levels = c(
    "Early Intermediate Period",
    "Middle Horizon",
    "Late Intermediate Period",
    "Nazca Late Intermediate Period"
  )
)
```

A linear model is fitted to compare the nitrogen isotope values (15N) across the different phases. This model examines whether there are statistically significant differences in 15N values between the phases.

```
lm_model_SH <- lm(d15N ~ Phase, data = SouthHighlands)
summary(lm_model_SH)
```

```
##
## Call:
## lm(formula = d15N ~ Phase, data = SouthHighlands)
##
## Residuals:
##     Min      1Q  Median      3Q     Max
## -2.6907 -0.8707 -0.0617  0.7346  5.4093
##
## Coefficients:
##                                       Estimate Std. Error t value Pr(>|t|)
## (Intercept)                             9.1907     0.1112  82.623  < 2e-16 ***
```

```
## PhaseMiddle Horizon                   -0.1897     0.1619  -1.172  0.24208
## PhaseLate Intermediate Period          0.6747     0.2572   2.623  0.00907 **
## PhaseNazca Late Intermediate Period   4.1709     0.1513  27.571  < 2e-16 ***
## ---
## Signif. codes:  0 '***' 0.001 '**' 0.01 '*' 0.05 '.' 0.1 ' ' 1
##
## Residual standard error: 1.182 on 369 degrees of freedom
## Multiple R-squared:  0.7452, Adjusted R-squared:  0.7431
## F-statistic: 359.8 on 3 and 369 DF,  p-value: < 2.2e-16
```

We calculate the estimated marginal means for 15N for each phase using the emmeans function, followed by pairwise comparisons with a Tukey adjustment to assess which phases significantly differ

```
emm_SH <- emmeans(lm_model_SH, ~ Phase)
print(emm_SH)
```

```
##  Phase                          emmean    SE  df lower.CL upper.CL
##  Early Intermediate Period        9.19 0.111 369     8.97     9.41
##  Middle Horizon                   9.00 0.118 369     8.77     9.23
##  Late Intermediate Period         9.87 0.232 369     9.41    10.32
##  Nazca Late Intermediate Period  13.36 0.103 369    13.16    13.56
##
## Confidence level used: 0.95
```

```
pairwise_comparisons_SH <- contrast(
  emm_SH,
  method = "pairwise",
  adjust = "tukey"
)
summary(pairwise_comparisons_SH)
```

```
##  contrast                                                  estimate    SE  df
##  Early Intermediate Period - Middle Horizon                   0.190 0.162 369
##  Early Intermediate Period - Late Intermediate Period        -0.675 0.257 369
##  Early Intermediate Period - Nazca Late Intermediate Period  -4.171 0.151 369
##  Middle Horizon - Late Intermediate Period                   -0.864 0.260 369
##  Middle Horizon - Nazca Late Intermediate Period             -4.361 0.156 369
##  Late Intermediate Period - Nazca Late Intermediate Period   -3.496 0.254 369
##  t.ratio p.value
##    1.172  0.6451
##   -2.623  0.0447
##  -27.571  <.0001
##   -3.324  0.0054
##  -27.941  <.0001
##  -13.789  <.0001
##
## P value adjustment: tukey method for comparing a family of 4 estimates
```

Cohen's d is used to measure the effect size for 15N differences between specific phases.

```r
data_EIP_NLIP <- SouthHighlands %>%
  filter(Phase %in% c("Early Intermediate Period",
                      "Nazca Late Intermediate Period")) %>%
  droplevels()
cohen_d_EIP_NLIP <- cohen.d(d15N ~ Phase, data = data_EIP_NLIP)
print(cohen_d_EIP_NLIP)
```

```
##
## Cohen's d
##
## d estimate: -3.405315 (large)
## 95 percent confidence interval:
##     lower     upper
## -3.798955 -3.011675
```

```r
# Late Intermediate Period vs. Nazca Late Intermediate Period
data_LIP_NLIP <- SouthHighlands %>%
  filter(Phase %in% c("Late Intermediate Period",
                      "Nazca Late Intermediate Period")) %>%
  droplevels()
cohen_d_LIP_NLIP <- cohen.d(d15N ~ Phase, data = data_LIP_NLIP)
print(cohen_d_LIP_NLIP)
```

```
##
## Cohen's d
##
## d estimate: -3.832903 (large)
## 95 percent confidence interval:
##     lower     upper
## -4.432590 -3.233216
```

```r
# Middle Horizon vs. Nazca Late Intermediate Period
data_MH_NLIP <- SouthHighlands %>%
  filter(Phase %in% c("Middle Horizon", "Nazca Late Intermediate Period")) %>%
  droplevels()
cohen_d_MH_NLIP <- cohen.d(d15N ~ Phase, data = data_MH_NLIP)
print(cohen_d_MH_NLIP)
```

```
##
## Cohen's d
##
## d estimate: -4.381065 (large)
## 95 percent confidence interval:
##     lower     upper
## -4.857326 -3.904804
```

```r
# Plot the data
ggplot(SouthHighlands, aes(x = Phase, y = d15N, fill = Phase)) +
  geom_boxplot(position = position_dodge(0.9)) +
  labs(x = "Phase", y = expression(delta^{15}*N)) +
  scale_fill_viridis_d(option = "viridis") +
```

```r
  theme_minimal() +
  theme(
    legend.position = "none",
    axis.text.x = element_text(angle = 45, hjust = 1)
  )
```

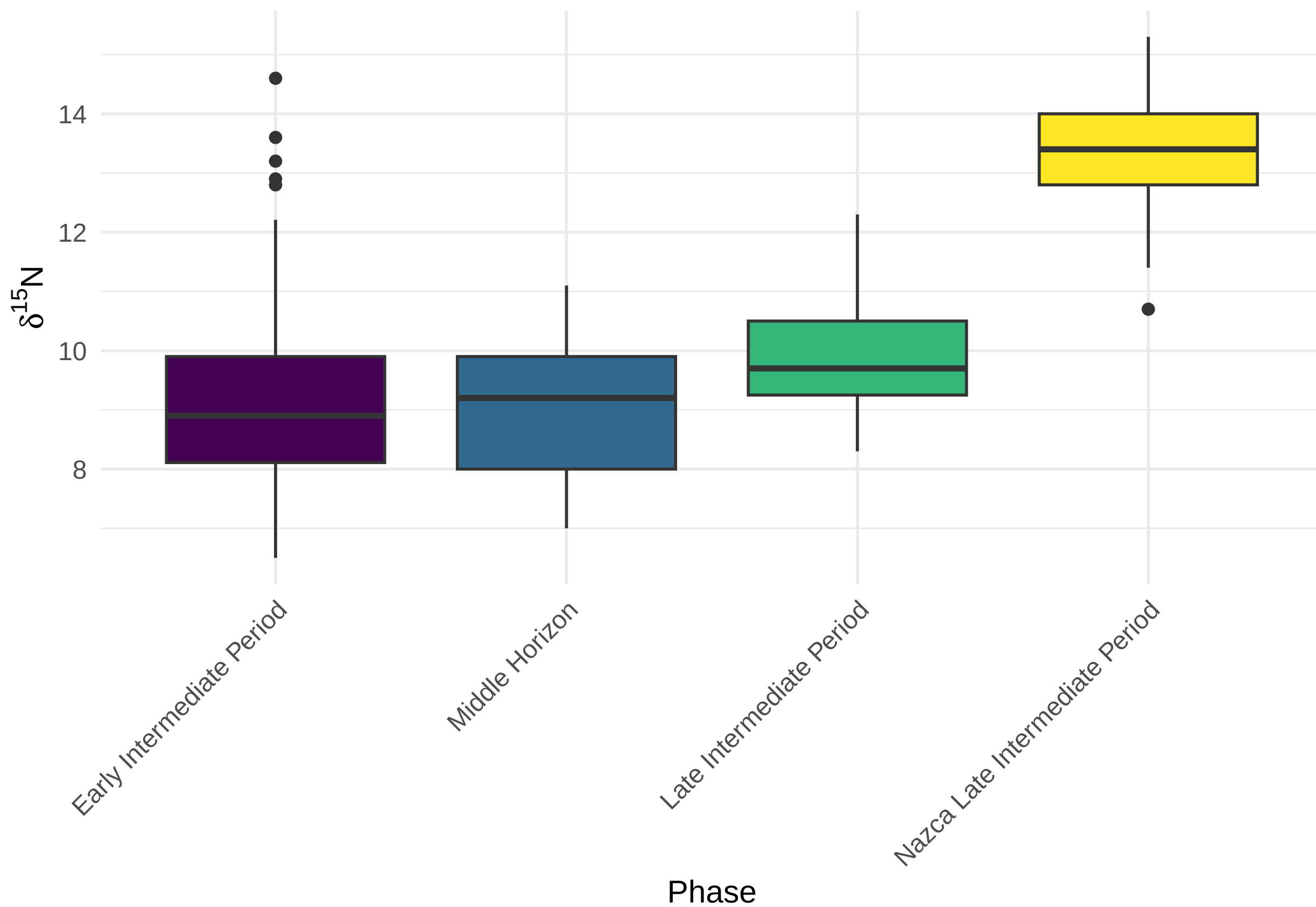

Fit the linear model on the adjusted dataset

```r
lm_model_SH <- lm(d15N ~ Phase, data = SouthHighlands)
summary(lm_model_SH)
```

```
##
## Call:
## lm(formula = d15N ~ Phase, data = SouthHighlands)
##
## Residuals:
##     Min      1Q  Median      3Q     Max
## -2.6907 -0.8707 -0.0617  0.7346  5.4093
##
## Coefficients:
##                                       Estimate Std. Error t value Pr(>|t|)
## (Intercept)                             9.1907     0.1112  82.623  < 2e-16 ***
## PhaseMiddle Horizon                    -0.1897     0.1619  -1.172  0.24208
## PhaseLate Intermediate Period           0.6747     0.2572   2.623  0.00907 **
## PhaseNazca Late Intermediate Period     4.1709     0.1513  27.571  < 2e-16 ***
## ---
```

```
## Signif. codes:  0 '***' 0.001 '**' 0.01 '*' 0.05 '.' 0.1 ' ' 1
##
## Residual standard error: 1.182 on 369 degrees of freedom
## Multiple R-squared:  0.7452, Adjusted R-squared:  0.7431
## F-statistic: 359.8 on 3 and 369 DF,  p-value: < 2.2e-16
```

```
# Calculate estimated marginal means for Phase
emm_SH_SH <- emmeans(lm_model_SH, ~ Phase)
print(emm_SH_SH)
```

```
##  Phase                          emmean    SE  df lower.CL upper.CL
##  Early Intermediate Period        9.19 0.111 369     8.97     9.41
##  Middle Horizon                   9.00 0.118 369     8.77     9.23
##  Late Intermediate Period         9.87 0.232 369     9.41    10.32
##  Nazca Late Intermediate Period  13.36 0.103 369    13.16    13.56
##
## Confidence level used: 0.95
```

```
# Perform pairwise comparisons with Tukey adjustment
pairwise_comparisons <- contrast(emm_SH_SH,
                                  method = "pairwise", adjust = "tukey")
summary(pairwise_comparisons)
```

```
##  contrast                                                 estimate    SE  df
##  Early Intermediate Period - Middle Horizon                  0.190 0.162 369
##  Early Intermediate Period - Late Intermediate Period       -0.675 0.257 369
##  Early Intermediate Period - Nazca Late Intermediate Period -4.171 0.151 369
##  Middle Horizon - Late Intermediate Period                  -0.864 0.260 369
##  Middle Horizon - Nazca Late Intermediate Period            -4.361 0.156 369
##  Late Intermediate Period - Nazca Late Intermediate Period  -3.496 0.254 369
##  t.ratio p.value
##    1.172  0.6451
##   -2.623  0.0447
##  -27.571  <.0001
##   -3.324  0.0054
##  -27.941  <.0001
##  -13.789  <.0001
##
## P value adjustment: tukey method for comparing a family of 4 estimates
```

0.4 Section 2.1: 15N and 13C

This analysis explores whether the elevated 15N values in the Nazca Highlanders during the Late Intermediate Period (LIP) could result from consuming fertilized crops

```
glm_model.carbon.cc <- lm(d15N ~ d13Cc, data = Nazca.data)
summary(glm_model.carbon.cc)
```

```
##
## Call:
## lm(formula = d15N ~ d13Cc, data = Nazca.data)
```

```
##
## Residuals:
##     Min      1Q  Median      3Q     Max
## -3.7142 -0.4915  0.0553  0.6352  2.0355
##
## Coefficients:
##             Estimate Std. Error t value Pr(>|t|)
## (Intercept) 13.43812    0.45466  29.556   <2e-16 ***
## d13Cc        0.01523    0.03531   0.431    0.667
## ---
## Signif. codes:  0 '***' 0.001 '**' 0.01 '*' 0.05 '.' 0.1 ' ' 1
##
## Residual standard error: 0.949 on 140 degrees of freedom
##   (8 observations deleted due to missingness)
## Multiple R-squared:  0.001327,   Adjusted R-squared:  -0.005807
## F-statistic: 0.186 on 1 and 140 DF,  p-value: 0.6669
```

```
#Visualize the effect of 13C (collagen) on 15N
custom_color <- "#2C4279"
visreg(glm_model.carbon.cc, "d13Cc", scale = "response", partial = FALSE,
       line = list(col = c("black")),
       xlab = expression(delta^13 * "Cc"), ylab = expression(delta^15 * "N"),
       cex.axis = 1.1, cex.lab = 1.2, fill = list(col = custom_color))
```

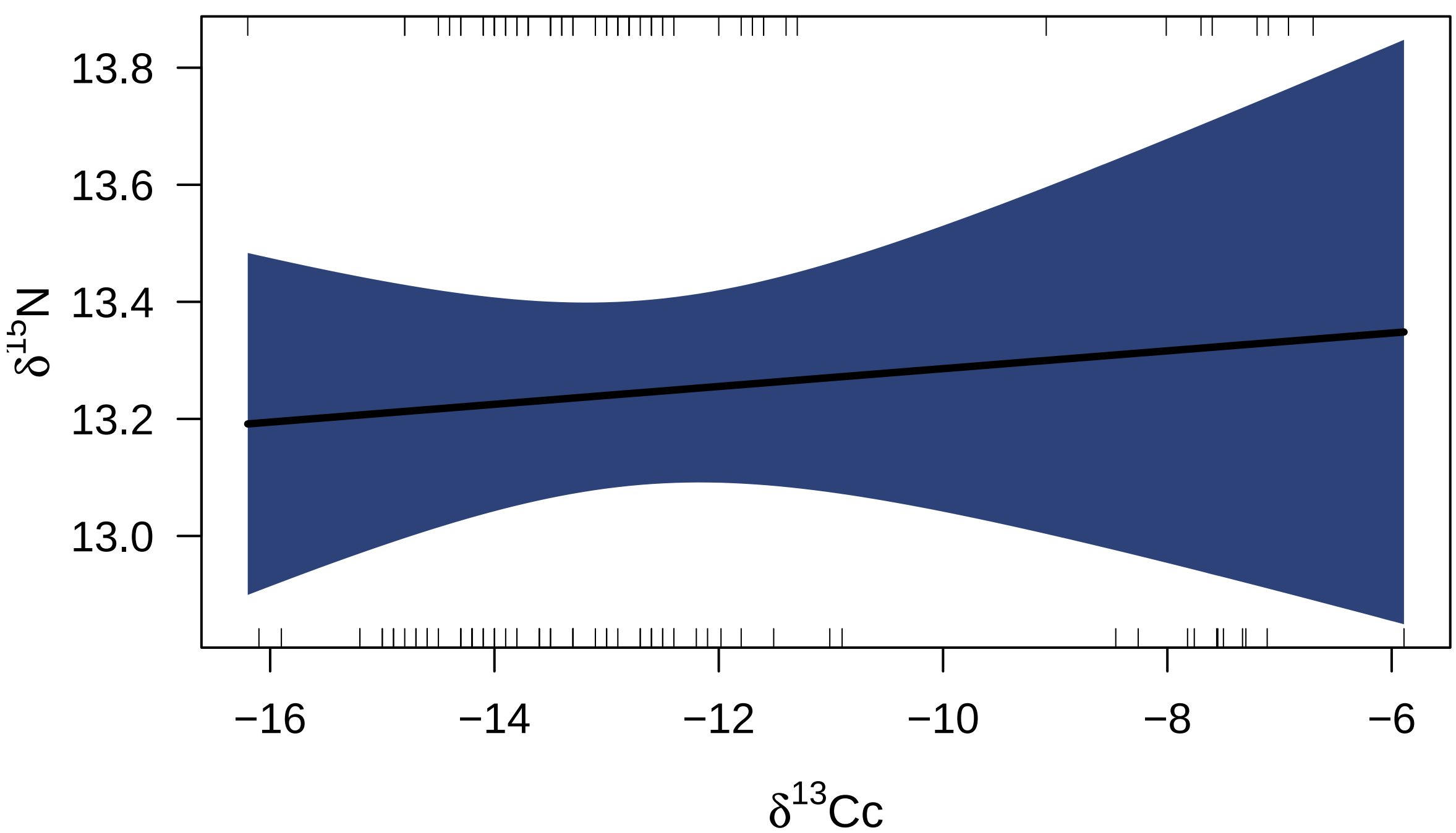

```r
glm_model.carbon.ca <- lm(d15N ~ d13Ca, data = Nazca.data)
summary(glm_model.carbon.ca)
```

```
##
## Call:
## lm(formula = d15N ~ d13Ca, data = Nazca.data)
##
## Residuals:
##      Min       1Q   Median       3Q      Max
## -2.87278 -0.43172  0.05027  0.58874  1.97185
##
## Coefficients:
##             Estimate Std. Error t value Pr(>|t|)
## (Intercept) 13.64827    0.35191  38.783   <2e-16 ***
## d13Ca        0.05274    0.03824   1.379     0.17
## ---
## Signif. codes:  0 '***' 0.001 '**' 0.01 '*' 0.05 '.' 0.1 ' ' 1
##
## Residual standard error: 0.9167 on 124 degrees of freedom
##   (24 observations deleted due to missingness)
## Multiple R-squared:  0.01511,    Adjusted R-squared:  0.007164
## F-statistic: 1.902 on 1 and 124 DF,  p-value: 0.1703
```

```r
custom_color <- "#2C4279"
visreg(glm_model.carbon.ca, "d13Ca", scale = "response", partial = FALSE,
       line = list(col = c("black")),
       xlab = expression(delta^13 * "Ca"), ylab = expression(delta^15 * "N"),
       cex.axis = 1.1, cex.lab = 1.2, fill = list(col = custom_color))
```

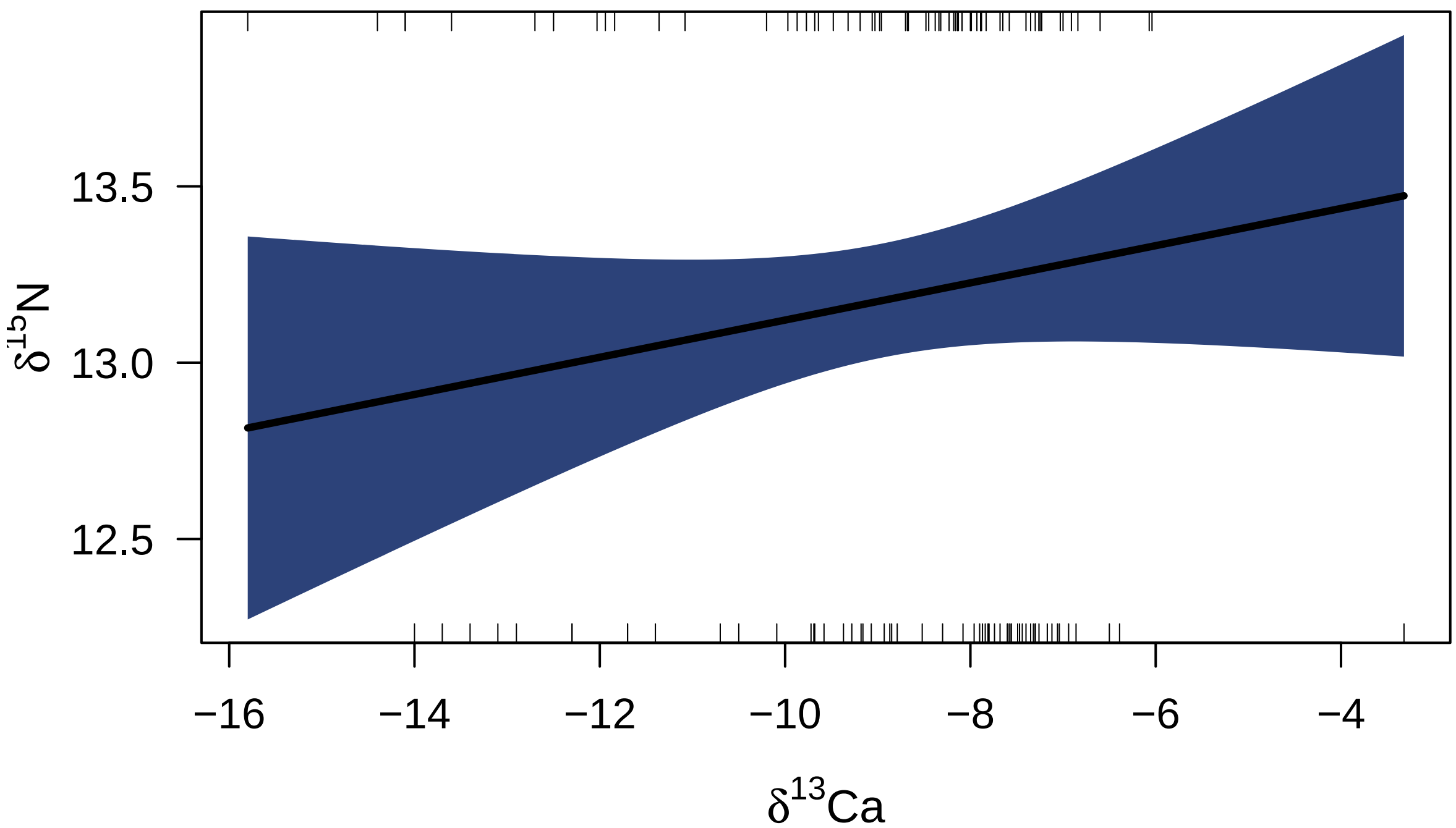

To focus on adult dietary practices and avoid confounding factors related to children's diets, the dataset is subset to exclude individuals categorized as "Child"

```
Nazca.data.adult <- subset(Nazca.data, age.categ != "Child")
```

0.5 Section 2.2: 15N v 34S

```
Nazca.LIP.34S <- subset(Nazca.data, !is.na(d34S))
```

A linear model is fitted to assess whether sulfur isotope values (34S) significantly predict nitrogen isotope values (15N).

```
glm_model.sulfur <- lm(d15N ~ d34S, data = Nazca.LIP.34S)
summary(glm_model.sulfur)
```

```
##
## Call:
## lm(formula = d15N ~ d34S, data = Nazca.LIP.34S)
##
## Residuals:
##      Min       1Q   Median       3Q      Max
## -2.42501 -0.31356  0.01475  0.47095  1.11199
##
## Coefficients:
```

```
##               Estimate Std. Error t value Pr(>|t|)
## (Intercept)  13.6401     0.5132  26.576   <2e-16 ***
## d34S         -0.1105     0.1216  -0.909    0.371
## ---
## Signif. codes:  0 '***' 0.001 '**' 0.01 '*' 0.05 '.' 0.1 ' ' 1
##
## Residual standard error: 0.7372 on 28 degrees of freedom
## Multiple R-squared:  0.02864,    Adjusted R-squared:  -0.006047
## F-statistic: 0.8257 on 1 and 28 DF,  p-value: 0.3713
```

```r
par(mfrow = c(2, 2))
plot(glm_model.sulfur)
```

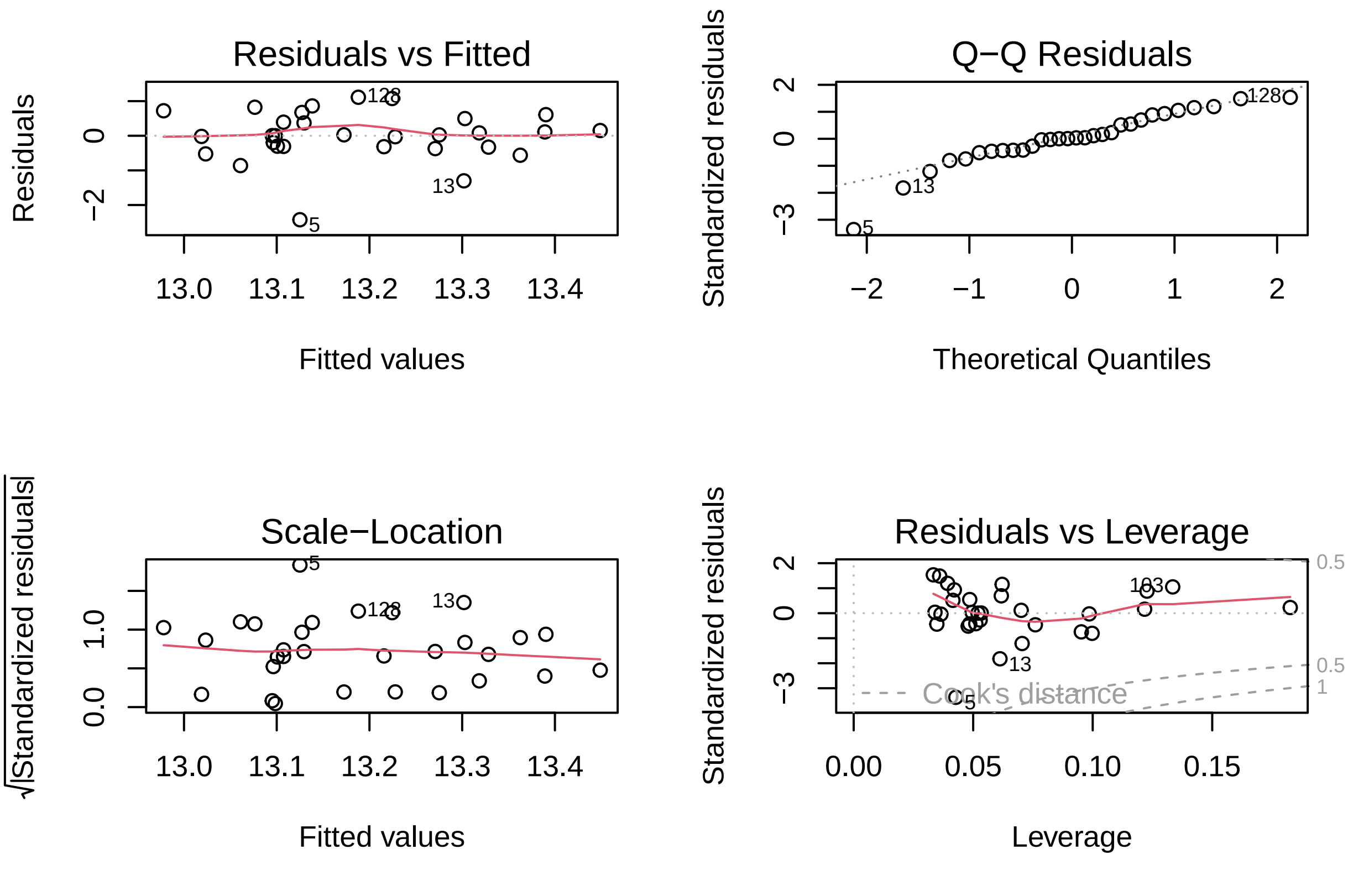


```r
custom_color <- "#2C4279"
visreg(glm_model.sulfur, "d34S", scale = "response", partial = FALSE,
       line = list(col = c("black")),
       xlab = expression(delta^35 * "S"), ylab = expression(delta^15 * "N"),
       cex.axis = 1.1, cex.lab = 1.2, fill = list(col = custom_color))
```

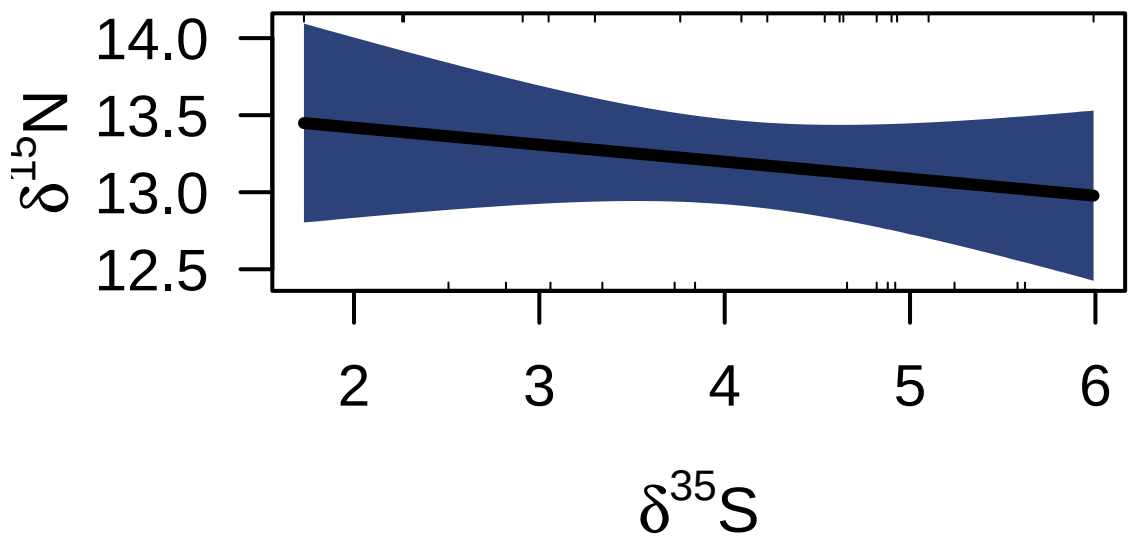

0.6 Section 2.3: 15N v Food resources

This analysis assesses 15N values in Nazca Highlanders during the Late Intermediate Period (LIP) against isotopic values compiled for food resources.

Combine Human and Food Resource datasets

```
Feeding.Baseline.local.d15N <- bind_rows(
  Feeding.Baseline %>%
    filter(local == "Local", !is.na(d15N)) %>%
    mutate(location = "Baseline") %>%
    select(d15N, type, location),

  Nazca.data %>%
    filter(grepl("^LIP", Phase), !is.na(d15N)) %>%
    mutate(
      type = "Nazca Human",
      location = "Nazca"
    ) %>%
    select(d15N, type, location)
) %>%
  mutate(type = factor(type)) %>%
  droplevels()
```

A Kruskal-Wallis test is used to compare 15N values between different local food resources

```r
kruskal_test_d15N_local <- kruskal.test(
  d15N ~ type,
  data = Feeding.Baseline.local.d15N
)

print(kruskal_test_d15N_local)
```

```
##
##  Kruskal-Wallis rank sum test
##
## data:  d15N by type
## Kruskal-Wallis chi-squared = 259.55, df = 6, p-value < 2.2e-16
```

```r
if (kruskal_test_d15N_local$p.value < 0.05) {
  pairwise.wilcox.test(
    Feeding.Baseline.local.d15N$d15N,
    Feeding.Baseline.local.d15N$type,
    p.adjust.method = "bonferroni",
    exact = FALSE
  )
}
```

```
##
##  Pairwise comparisons using Wilcoxon rank sum test with continuity correction
##
## data:  Feeding.Baseline.local.d15N$d15N and Feeding.Baseline.local.d15N$type
##
##               C3 cultivated C3 wild C4 cultivated large mammal medium mammal
## C3 wild       1.00000       -       -             -            -
## C4 cultivated 0.02117       0.99164 -             -            -
## large mammal  1.3e-06       0.27693 0.37955       -            -
## medium mammal 0.04608       0.78406 1.00000       0.54777      -
## Nazca Human   3.8e-13       0.01421 0.02315       < 2e-16      0.00364
## small mammal  0.00011       0.21498 1.00000       1.00000      1.00000
##               Nazca Human
## C3 wild       -
## C4 cultivated -
## large mammal  -
## medium mammal -
## Nazca Human   -
## small mammal  3.2e-14
##
## P value adjustment method: bonferroni
```

```r
Feeding.Baseline.local.d15N$type <- factor(
  Feeding.Baseline.local.d15N$type,
  levels = c(
    "C3 cultivated",
    "C3 wild",
    "C4 cultivated",
    "CAM",
    "small mammal",
```

```
    "medium mammal",
    "large mammal",
    "Nazca Human"
  )
)

ggplot(Feeding.Baseline.local.d15N, aes(x = type, y = d15N, fill = type)) +
  geom_boxplot(position = position_dodge(0.9)) +
  labs(
    title = expression(delta^{15}*N),
    x = "",
    y = expression(delta^{15}*N)
  ) +
  scale_fill_viridis_d(option = "viridis") +
  theme_minimal() +
  theme(
    legend.position = "none",
    axis.text.x = element_text(angle = 45, hjust = 1, vjust = 1)
  )
```

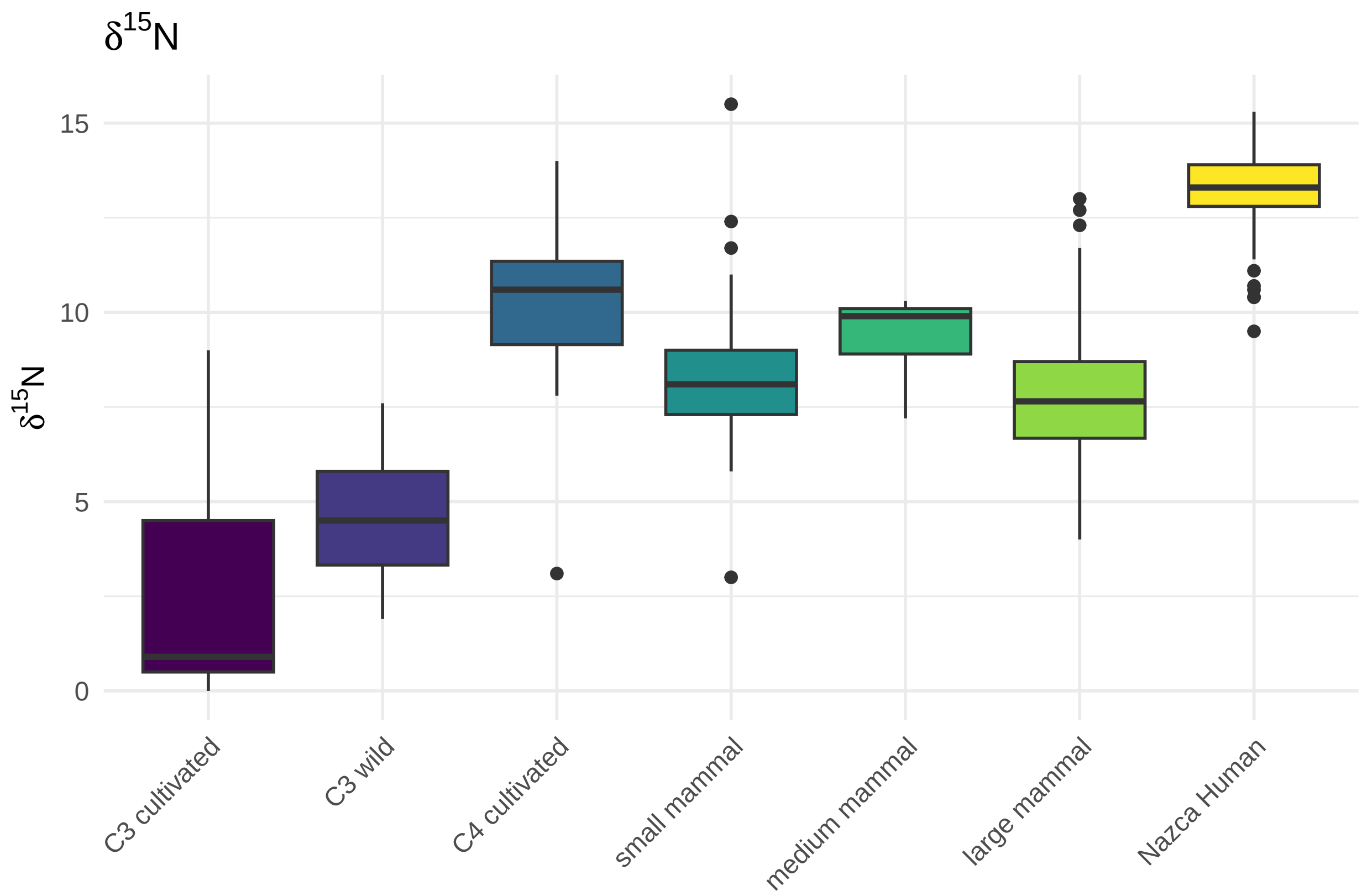

Add in non-local aquatic (marine and freshwater) food resources

```
Feeding.Baseline.local.d15N2 <- bind_rows(
  Feeding.Baseline %>%
    filter(
      !is.na(d15N),
```

```
      !is.na(type),
      local == "Local" | type %in% c("freshwater fish", "marine fish")
    ) %>%
    mutate(location = "Baseline"),

  Nazca.data %>%
    filter(Phase2 == "LIP", !is.na(d15N)) %>%
    mutate(
      type = "Nazca Human",
      location = "Nazca"
    )
) %>%
  select(d15N, type, location) %>%
  distinct() %>%
  droplevels()
```

A Kruskal-Wallis test is used to compare 15N values between different food resources

```
kruskal_test_d15N_local2 <- kruskal.test(
  d15N ~ type,
  data = Feeding.Baseline.local.d15N2
)

print(kruskal_test_d15N_local2)
```

```
##
##  Kruskal-Wallis rank sum test
##
## data:  d15N by type
## Kruskal-Wallis chi-squared = 128.26, df = 8, p-value < 2.2e-16
```

```
if (kruskal_test_d15N_local2$p.value < 0.05) {
  pairwise_test_local2 <- suppressWarnings(
    pairwise.wilcox.test(
      Feeding.Baseline.local.d15N2$d15N,
      Feeding.Baseline.local.d15N2$type,
      p.adjust.method = "bonferroni",
      exact = FALSE
    )
  )
  print(pairwise_test_local2)
}
```

```
##
##  Pairwise comparisons using Wilcoxon rank sum test with continuity correction
##
## data:  Feeding.Baseline.local.d15N2$d15N and Feeding.Baseline.local.d15N2$type
##
##                 C3 cultivated C3 wild C4 cultivated freshwater fish
## C3 wild         1.0000        -       -             -
## C4 cultivated   0.1021        1.0000  -             -
## freshwater fish 1.0000        1.0000  1.0000        -
```

```
## large mammal    0.0050       0.8375  1.0000       1.0000
## marine fish     3.5e-07      0.0520  0.2531       0.5689
## medium mammal   0.1726       1.0000  1.0000       1.0000
## Nazca Human     1.1e-07      0.0422  0.1708       0.3146
## small mammal    0.0098       0.4084  1.0000       1.0000
##                 large mammal marine fish medium mammal Nazca Human
## C3 wild         -            -           -             -
## C4 cultivated   -            -           -             -
## freshwater fish -            -           -             -
## large mammal    -            -           -             -
## marine fish     8.4e-12      -           -             -
## medium mammal   1.0000       0.0939      -             -
## Nazca Human     8.9e-14      1.0000      0.0177        -
## small mammal    1.0000       1.1e-06     1.0000        9.6e-08
##
## P value adjustment method: bonferroni
```

```
Feeding.Baseline.local.d15N2$type <- factor(
  Feeding.Baseline.local.d15N2$type,
  levels = c(
    "C3 cultivated",
    "C3 wild",
    "C4 cultivated",
    "CAM",
    "freshwater fish",
    "marine fish",
    "small mammal",
    "medium mammal",
    "large mammal",
    "Nazca Human"
  )
)

ggplot(Feeding.Baseline.local.d15N2, aes(x = type, y = d15N, fill = type)) +
  geom_boxplot(position = position_dodge(0.9)) +
  labs(
    title = expression(delta^{15}*N),
    x = "",
    y = expression(delta^{15}*N)
  ) +
  scale_fill_viridis_d(option = "viridis") +
  theme_minimal() +
  theme(
    legend.position = "none",
    axis.text.x = element_text(angle = 45, hjust = 1, vjust = 1)
  )
```

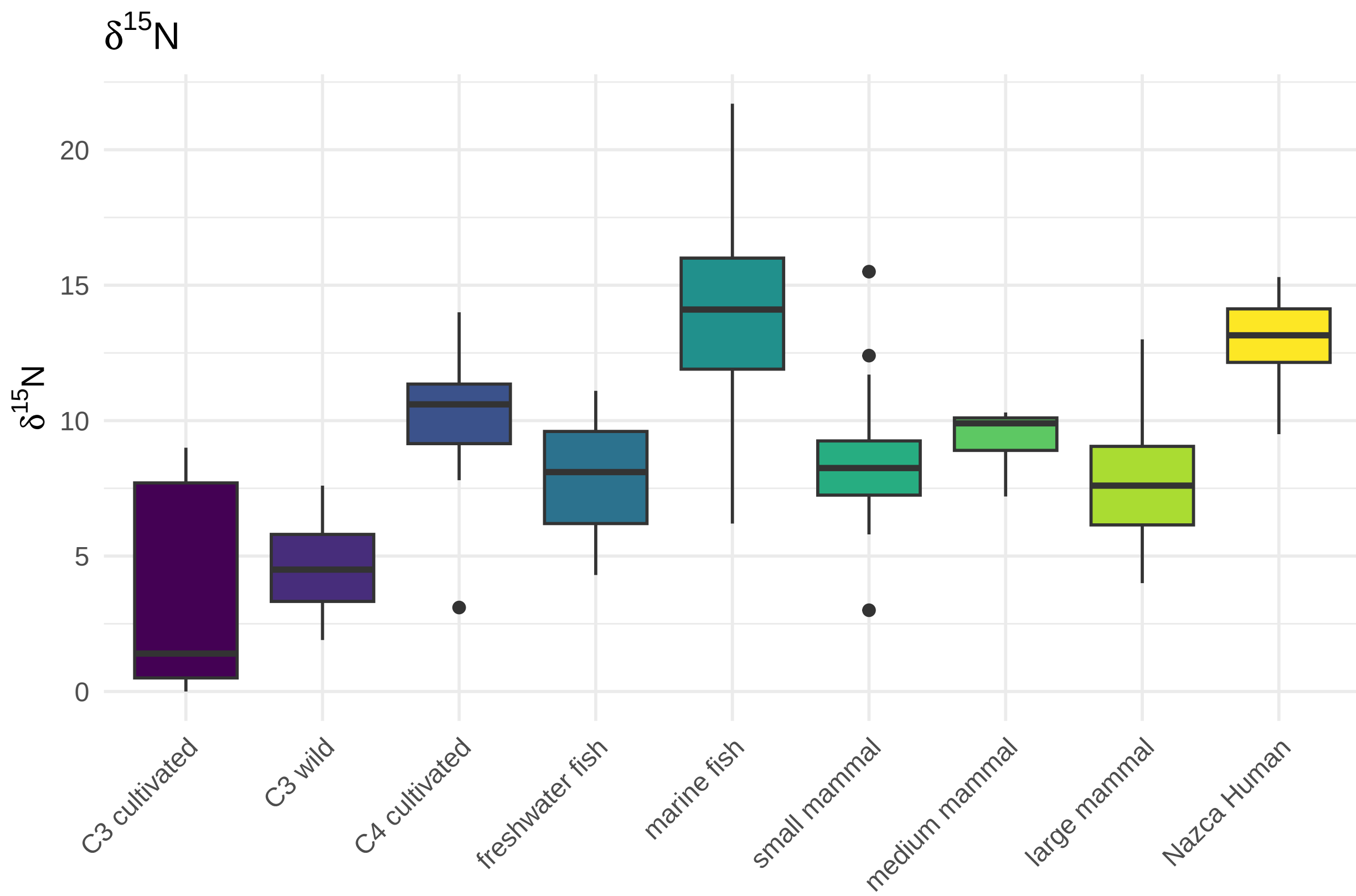

Add in non-local aquatic (marine and freshwater) food resources

```
Feeding.Baseline.local.d15N2 <- bind_rows(
  Feeding.Baseline %>%
    filter(
      !is.na(d15N),
      !is.na(type),
      local == "Local" | type %in% c("freshwater fish", "marine fish")
    ) %>%
    mutate(location = "Baseline"),

  Nazca.data %>%
    filter(Phase2 == "LIP", !is.na(d15N)) %>%
    mutate(
      type = "Nazca Human",
      location = "Nazca"
    )
) %>%
  select(d15N, type, location) %>%
  distinct() %>%
  droplevels()
```

A Kruskal-Wallis test is used to compare 34S values between different food resources

```
Feeding.Baseline.d34S <- bind_rows(
  Feeding.Baseline %>%
```

```r
    filter(!is.na(d34S)) %>%
    mutate(location = "Baseline") %>%
    select(d34S, type, location),

  Nazca.data %>%
    filter(Phase2 == "LIP", !is.na(d34S)) %>%
    mutate(
      type = "Nazca Human",
      location = "Nazca"
    ) %>%
    select(d34S, type, location)
) %>%
  distinct() %>%
  droplevels()


kruskal_test_d34S <- kruskal.test(
  d34S ~ type,
  data = Feeding.Baseline.d34S
)

print(kruskal_test_d34S)
```

```
##
##  Kruskal-Wallis rank sum test
##
## data:  d34S by type
## Kruskal-Wallis chi-squared = 53.372, df = 4, p-value = 7.123e-11
```

```r
if (kruskal_test_d34S$p.value < 0.05) {
  pairwise_test_d34S <- suppressWarnings(
    pairwise.wilcox.test(
      Feeding.Baseline.d34S$d34S,
      Feeding.Baseline.d34S$type,
      p.adjust.method = "bonferroni",
      exact = FALSE
    )
  )
  print(pairwise_test_d34S)
}
```

```
##
##  Pairwise comparisons using Wilcoxon rank sum test with continuity correction
##
## data:  Feeding.Baseline.d34S$d34S and Feeding.Baseline.d34S$type
##
##              C4 cultivated large mammal marine fish Nazca Human
## large mammal 0.0739        -            -           -
## marine fish  0.0774        2.2e-06      -           -
## Nazca Human  0.0803        0.0040       1.2e-07     -
## small mammal 0.2265        1.0000       0.0016      0.1396
##
## P value adjustment method: bonferroni
```

```r
Feeding.Baseline.d34S$type <- factor(
  Feeding.Baseline.d34S$type,
  levels = c(
    "C4 cultivated",
    "marine fish",
    "small mammal",
    "large mammal",
    "Nazca Human"
  )
)

ggplot(Feeding.Baseline.d34S, aes(x = type, y = d34S, fill = type)) +
  geom_boxplot(position = position_dodge(0.9)) +
  labs(
    title = expression(delta^{34}*S),
    x = "",
    y = expression(delta^{34}*S)
  ) +
  scale_fill_viridis_d(option = "viridis") +
  theme_minimal() +
  theme(
    legend.position = "none",
    axis.text.x = element_text(angle = 45, hjust = 1, vjust = 1)
  )
```

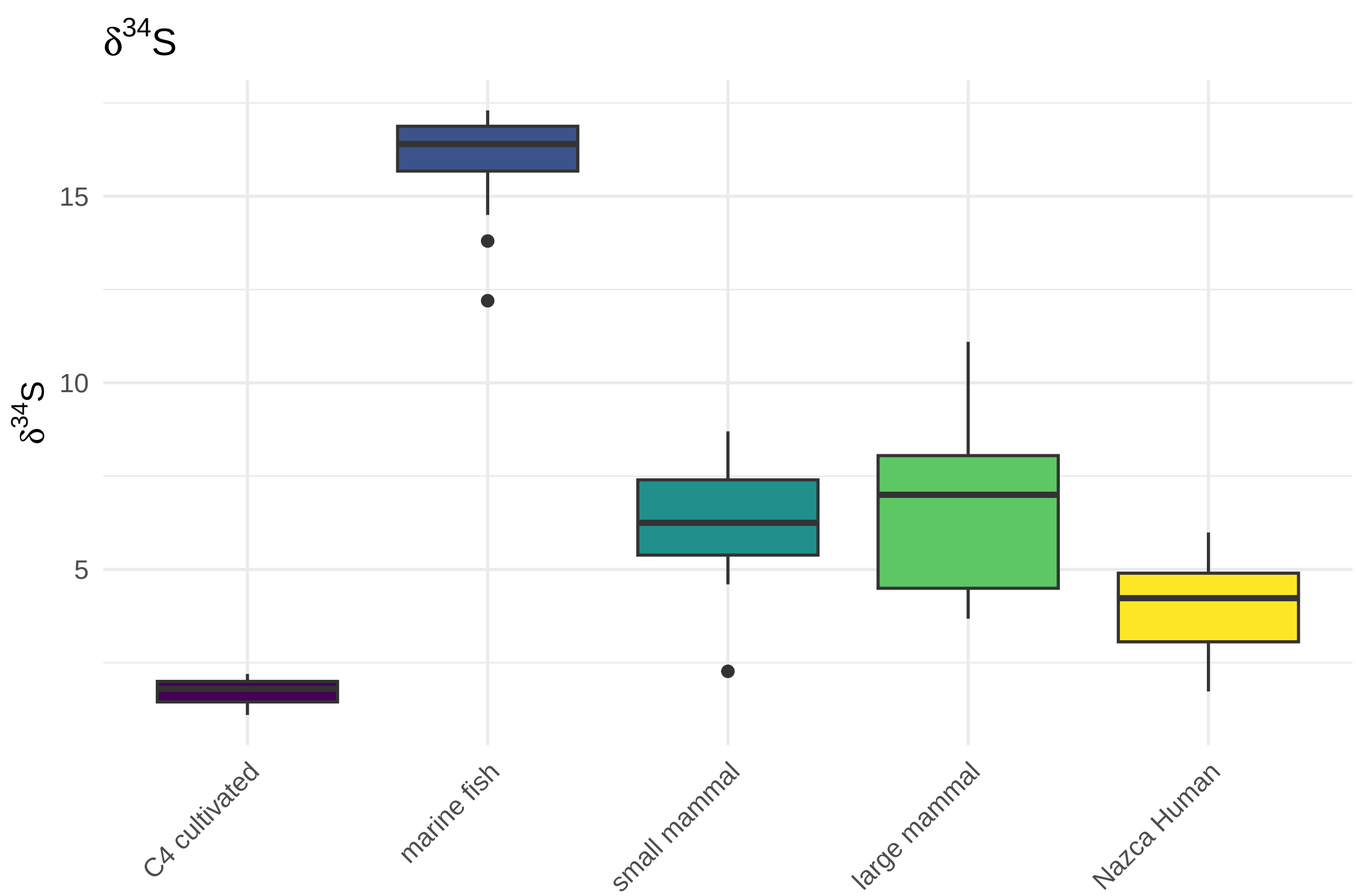

0.7 Section 4: Influence of climate

0.8 Section 4.1: d15N-quelccaya_d18O Full Andes Analysis

This analysis examines whether climate change, specifically aridity during the Late Intermediate Period (LIP), was responsible for elevated 15N values in the Nazca Highlands.

Simulating Random Years for 15N Data: To account for uncertainty in the dating of human remains, a function is used to simulate random years within the provided BP (Before Present) ranges for each individual.Finding the Nearest Lagged 18O Value, Monte Carlo Simulation for Lagged 18O and 15N Correlation

```
simulate_one_round <- function(start_bp, end_bp) {
  if (!is.na(start_bp) && !is.na(end_bp)) {
    return(round(runif(1, min = end_bp, max = start_bp)))
  } else {
    return(NA)
  }
}

find_nearest_lagged_d18O <- function(year, climate.d18O) {
  nearest_year_index <- which.min(abs(climate.d18O$year.bp - year))
  return(climate.d18O$lagged_dO18[nearest_year_index])
}

simulate_correlation_lagged <- function(data, climate.d18O, iterations = 10000) {

  correlation_results <- numeric(iterations)

  for (i in 1:iterations) {

    data$SimulatedYearBP <- mapply(
      simulate_one_round,
      data$Start.BP,
      data$End.BP
    )

    data$Nearest_lagged_d18O <- sapply(
      data$SimulatedYearBP,
      find_nearest_lagged_d18O,
      climate.d18O = climate.d18O
    )

    data$Nearest_lagged_d18O <- as.numeric(data$Nearest_lagged_d18O)

    valid_indices <- complete.cases(data$d15N, data$Nearest_lagged_d18O)

    if (sum(valid_indices) > 2) {
      correlation_results[i] <- cor(
        data$d15N[valid_indices],
        data$Nearest_lagged_d18O[valid_indices],
        method = "pearson"
      )
    } else {
      correlation_results[i] <- NA
```

```
    }
  }

  correlation_results <- correlation_results[!is.na(correlation_results)]
  return(correlation_results)
}
```

Sorting Climate Data and Calculating a 10-Year Moving Average, Conduct Monte Carlo simulation for lagged 18O for Human LIP data

```
climate.d18O <- climate.d18O[order(climate.d18O$year.bp),]
climate.d18O$ma10yr <- rollmean(climate.d18O$dO18, 10, fill = NA,
                                align = 'center')
# Shift  18O values by 10 years to create a lagged column
climate.d18O$lagged_dO18 <- c(rep(NA, 10),
                              climate.d18O$dO18[1:(nrow(climate.d18O) - 10)]
  )

correlation_results_lagged <- simulate_correlation_lagged(Human.LIP, climate.d18O)
summary_statistics_lagged <- summary(correlation_results_lagged)
hist(correlation_results_lagged, breaks = 50,
     main = "Distribution of Lagged  18O Correlation Coefficients",
     xlab = "Correlation Coefficient")
```

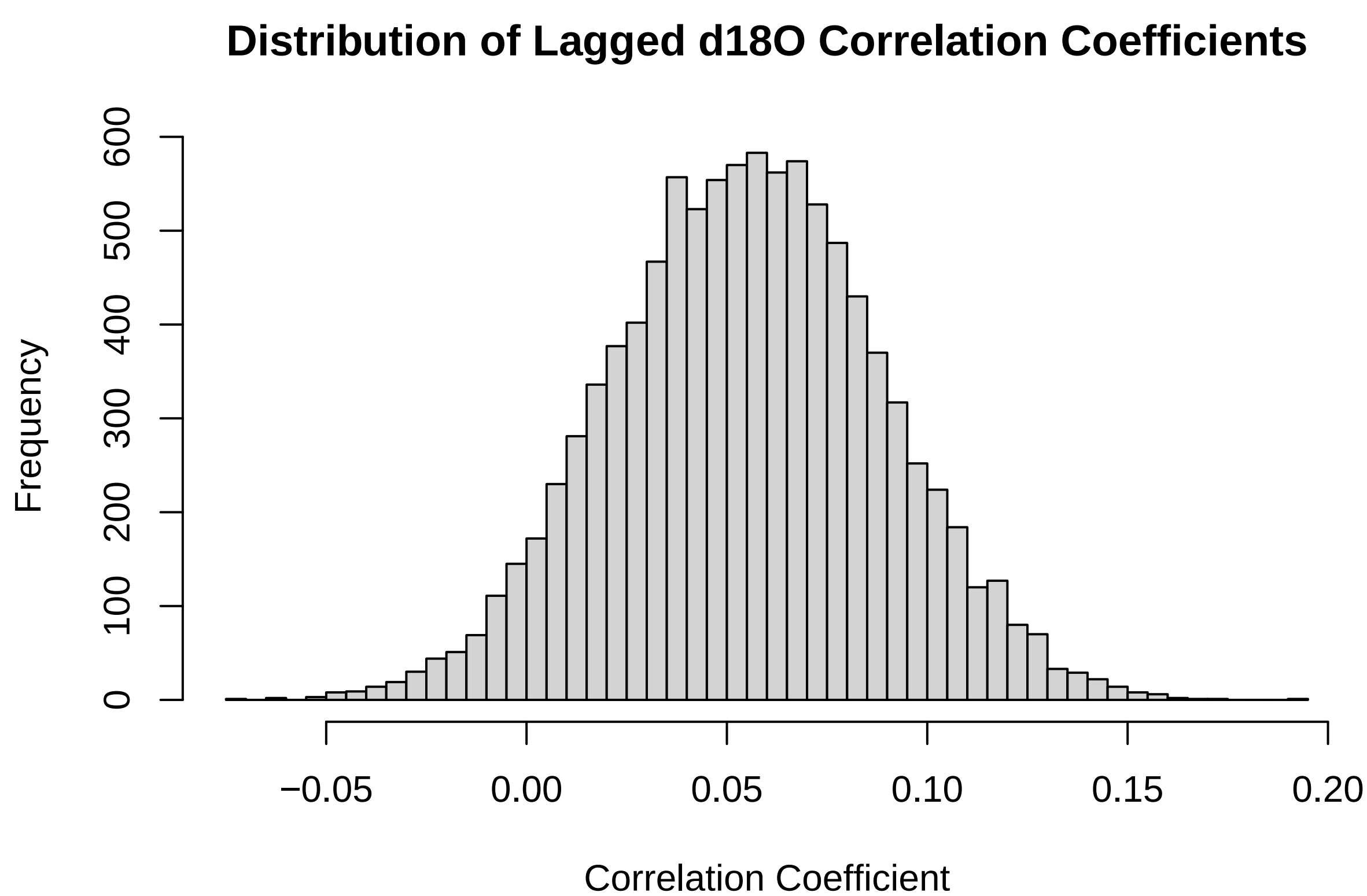

```r
percentage_positive_correlation_lagged <-
  sum(correlation_results_lagged > 0) / length(correlation_results_lagged) * 100
percentage_negative_correlation_lagged <-
  sum(correlation_results_lagged < 0) / length(correlation_results_lagged) * 100
print(summary_statistics_lagged)
```

```
##     Min.  1st Qu.   Median     Mean  3rd Qu.     Max.
## -0.07064  0.03221  0.05534  0.05506  0.07776  0.19154
```

```r
print(paste("Percentage of positive correlations:",
            percentage_positive_correlation_lagged))
```

```
## [1] "Percentage of positive correlations: 94.94"
```

```r
print(paste("Percentage of negative correlations:",
            percentage_negative_correlation_lagged))
```

```
## [1] "Percentage of negative correlations: 5.06"
```

```r
plot(density(correlation_results_lagged),
     main = "Density of Lagged  18O Correlation Coefficients",
     xlab = "Correlation Coefficient", ylab = "Density")
```

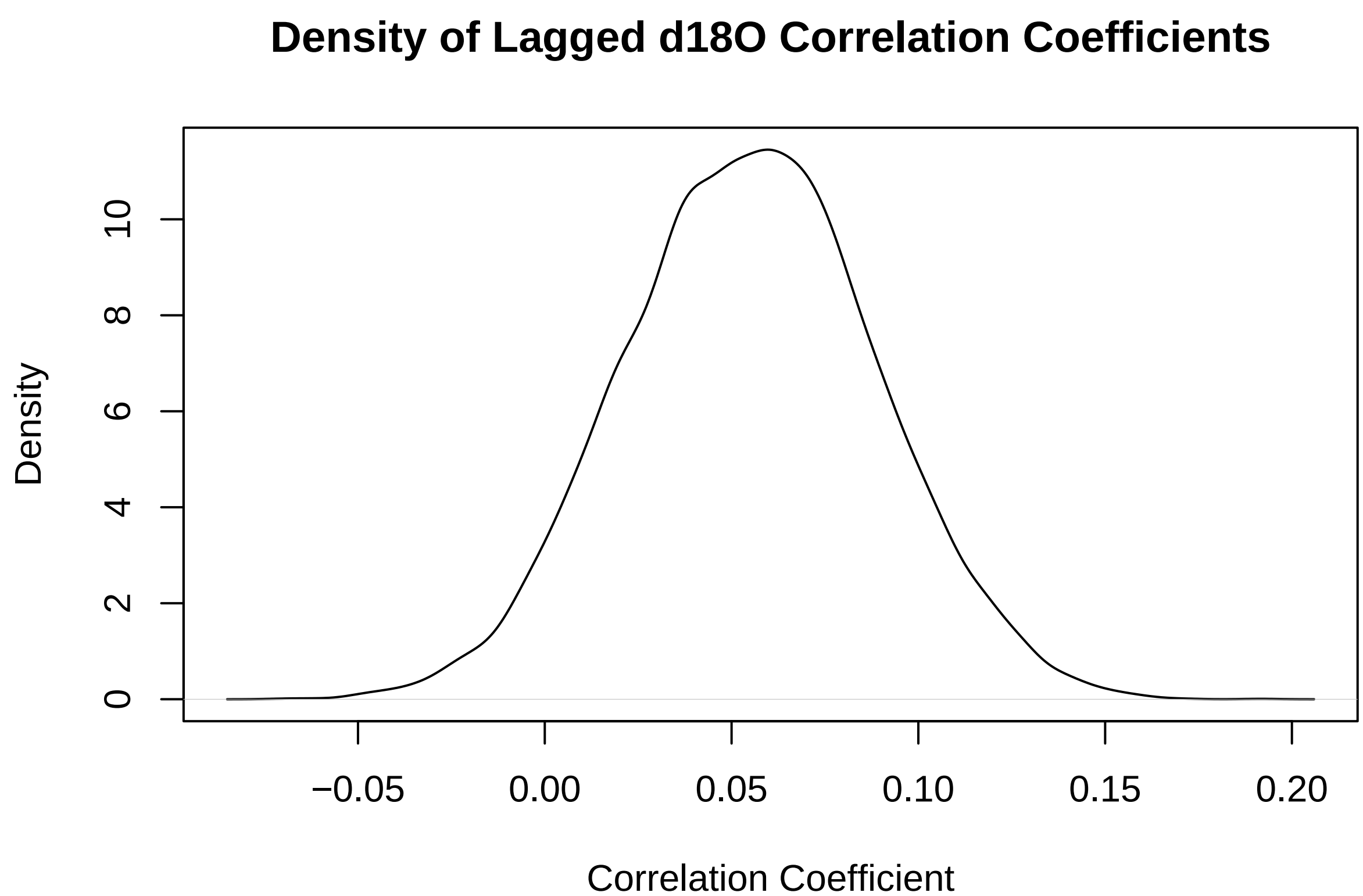

Statistical Testing of Correlation Results: A t-test is conducted to determine whether the correlation coefficients are significantly different from zero, and 95% confidence intervals are calculated

```r
t_test_results_lagged <- t.test(correlation_results_lagged, mu = 0)
print(t_test_results_lagged)
```

```
##
##  One Sample t-test
##
## data:  correlation_results_lagged
## t = 163.57, df = 9999, p-value < 2.2e-16
## alternative hypothesis: true mean is not equal to 0
## 95 percent confidence interval:
##  0.05439565 0.05571519
## sample estimates:
##  mean of x
## 0.05505542
```

Confidence intervals for the correlation coefficients are calculated using quantiles to provide a statistical range for the correlation results

```r
conf_int_lagged <- quantile(correlation_results_lagged, probs = c(0.025, 0.975))
print(paste("95% confidence interval for the correlation coefficient:",
            conf_int_lagged[1], "to", conf_int_lagged[2]))
```

```
## [1] "95% confidence interval for the correlation coefficient: -0.00996978888709378 to 0.121058733513
```

A scatter plot is generated to visually explore the relationship between 15N and lagged 18O.

```r
Human.LIP$SimulatedYearBP <-
  mapply(simulate_one_round, Human.LIP$Start.BP, Human.LIP$End.BP)
Human.LIP$Nearest_lagged_d18O <-
  sapply(Human.LIP$SimulatedYearBP, find_nearest_lagged_d18O,
         climate.d18O = climate.d18O)
Human.LIP$Nearest_lagged_d18O <-
  as.numeric(Human.LIP$Nearest_lagged_d18O)
filtered_data <- Human.LIP[!is.na(Human.LIP$d15N) &
                             !is.na(Human.LIP$Nearest_lagged_d18O), ]
model <- lm(d15N ~ Nearest_lagged_d18O, data = filtered_data)
filtered_data$residuals <- abs(resid(model))

ggplot(filtered_data, aes(x = Nearest_lagged_d18O, y = d15N,
                          color = residuals)) +
  geom_point(alpha = 0.7) +
  geom_smooth(method = 'lm', color = 'black', se = FALSE) +
  labs(title = "Scatter Plot of  15N vs Lagged  18O",
       x = expression(delta^18 * O ~ "lagged"),
       y = expression(delta^{15}*N), color = "Distance from Line") +
  scale_color_viridis(option = "viridis") +
  theme_minimal()
```

```
## `geom_smooth()` using formula = 'y ~ x'
```

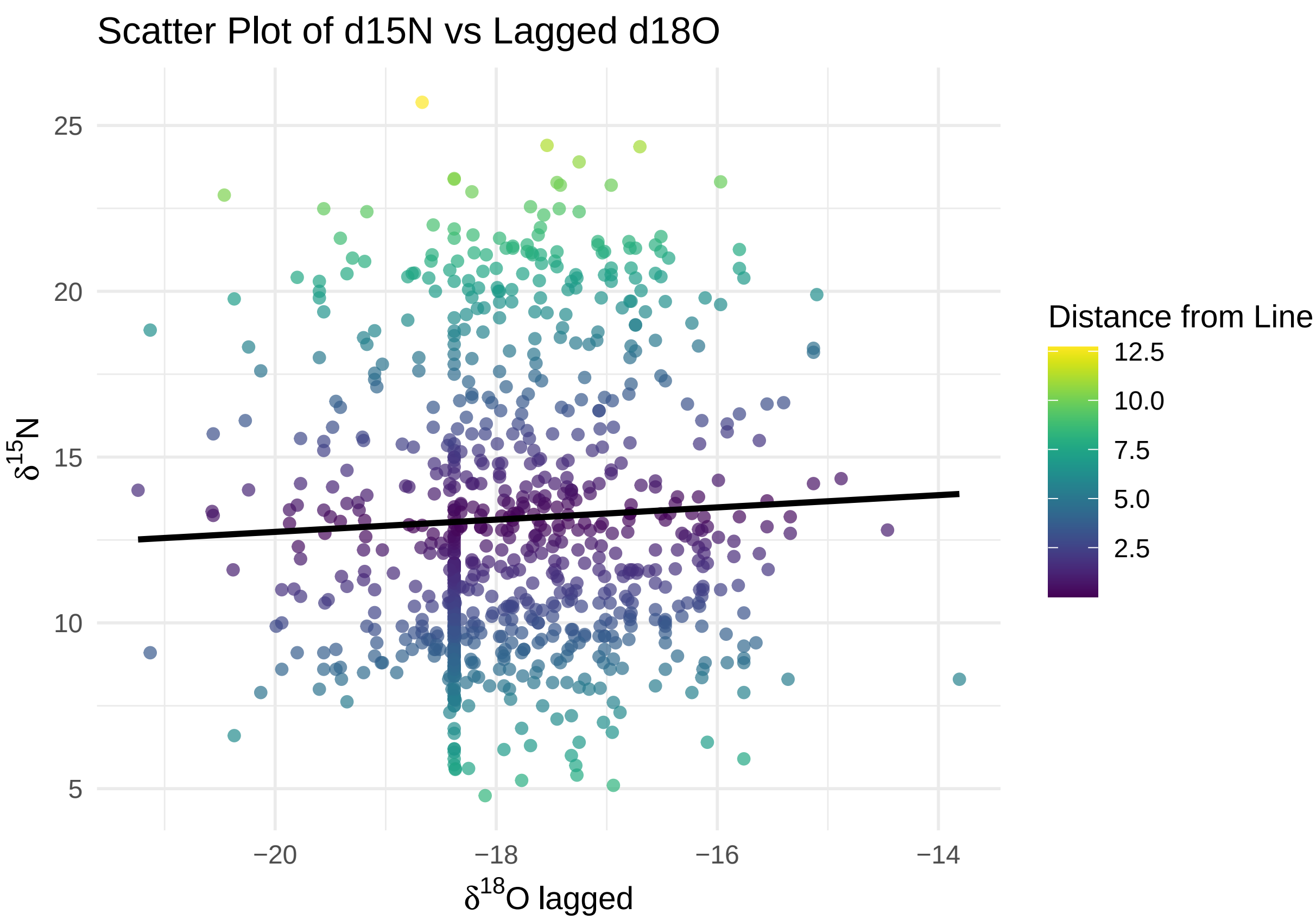

0.9 Section 4.2: d15N-quelccaya_d18O South Highlands Analysis

This analysis focuses on the South Highlands region of the Andes to assess the relationship between 15N values and climate changes, particularly using 18O as a climate proxy. Severe droughts during the Late Intermediate Period (LIP) are hypothesized to have influenced nitrogen isotope values by concentrating nitrogen in soils and water.

```
SouthHighlands <- subset(Human.LIP, Region == "South Highlands")
```

The Monte Carlo simulation from earlier is applied to the South Highlands dataset to compute correlations between 15N values and lagged 18O across 10,000 iterations.

```
correlation_results_lagged_SouthHighlands <-
  simulate_correlation_lagged(SouthHighlands, climate.d18O)

summary_statistics_lagged_SouthHighlands <-
  summary(correlation_results_lagged_SouthHighlands)
hist(correlation_results_lagged_SouthHighlands,
     breaks = 50,
     main = "Lagged  18O Correlation Coefficients (South Highlands)",
     xlab = "Correlation Coefficient")
```

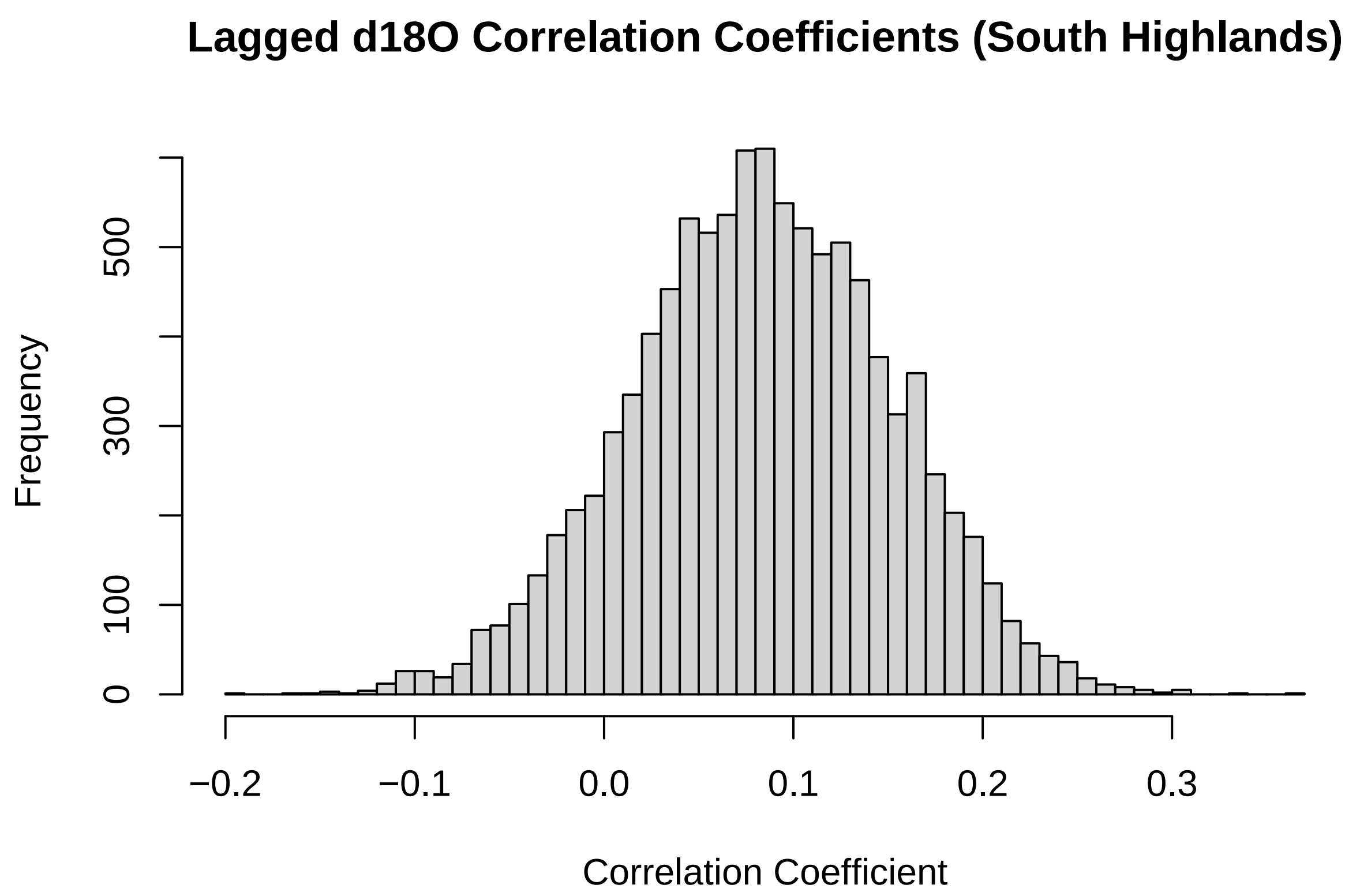


```r
percentage_positive_correlation_lagged_SouthHighlands <-
  sum(correlation_results_lagged_SouthHighlands > 0) /
  length(correlation_results_lagged_SouthHighlands) * 100
percentage_negative_correlation_lagged_SouthHighlands <-
  sum(correlation_results_lagged_SouthHighlands < 0) /
  length(correlation_results_lagged_SouthHighlands) * 100
print(summary_statistics_lagged_SouthHighlands)
```

```
##     Min.  1st Qu.   Median     Mean  3rd Qu.     Max.
## -0.19620  0.03802  0.08344  0.08333  0.13072  0.36149
```

```r
print(paste("Percentage of positive correlations:",
            percentage_positive_correlation_lagged_SouthHighlands))
```

```
## [1] "Percentage of positive correlations: 88.83"
```

```r
print(paste("Percentage of negative correlations:",
            percentage_negative_correlation_lagged_SouthHighlands))
```

```
## [1] "Percentage of negative correlations: 11.17"
```

To test the statistical significance of the correlation results, a t-test is conducted

```r
t_test_results_lagged_SouthHighlands <-
  t.test(correlation_results_lagged_SouthHighlands, mu = 0)
print(t_test_results_lagged_SouthHighlands)
```

```
##
##  One Sample t-test
##
## data:  correlation_results_lagged_SouthHighlands
## t = 121.99, df = 9999, p-value < 2.2e-16
## alternative hypothesis: true mean is not equal to 0
## 95 percent confidence interval:
##  0.08198739 0.08466525
## sample estimates:
##  mean of x
## 0.08332632
```

```r
conf_int_lagged_SouthHighlands <-
  quantile(correlation_results_lagged_SouthHighlands, probs = c(0.025, 0.975))
print(paste("95% confidence interval for  correlation coefficient (South Highlands):",
            conf_int_lagged_SouthHighlands[1], "to",
            conf_int_lagged_SouthHighlands[2]))
```

```
## [1] "95% confidence interval for  correlation coefficient (South Highlands): -0.0530568661521371 to
```

To incorporate uncertainty in the dating of human remains, the simulate_one_round function is used to generate random years for each individual within their known BP ranges

```r
SouthHighlands$SimulatedYearBP <-
  mapply(simulate_one_round, SouthHighlands$Start.BP, SouthHighlands$End.BP)
SouthHighlands$Nearest_lagged_d18O <-
  sapply(SouthHighlands$SimulatedYearBP, find_nearest_lagged_d18O,
         climate.d18O = climate.d18O)
SouthHighlands$Nearest_lagged_d18O <-
  as.numeric(SouthHighlands$Nearest_lagged_d18O)
filtered_data_SouthHighlands <-
  SouthHighlands[!is.na(SouthHighlands$d15N) &
                 !is.na(SouthHighlands$Nearest_lagged_d18O), ]
```

A linear model is then fitted to explore the relationship between 15N and the nearest lagged 18O values in the filtered dataset.

```r
model_SouthHighlands <- lm(d15N ~ Nearest_lagged_d18O,
                           data = filtered_data_SouthHighlands)
filtered_data_SouthHighlands$residuals <- abs(resid(model_SouthHighlands))
```

To visually explore the relationship between 15N and lagged 18O in the South Highlands, a scatter plot is generated

```r
ggplot(filtered_data_SouthHighlands, aes(x = Nearest_lagged_d18O,
                                         y = d15N, color = residuals)) +
  geom_point(alpha = 0.7) +
```

```
  geom_smooth(method = 'lm', color = 'black', se = FALSE) +
  labs(title = "Scatter Plot of  15N vs Lagged  18O (South Highlands)",
       x = expression(delta^18 * O ~ "lagged"),
       y = expression(delta^{15}*N), color = "Distance from Line") +
  scale_color_viridis(option = "viridis") +
  theme_minimal()
```

```
## `geom_smooth()` using formula = 'y ~ x'
```

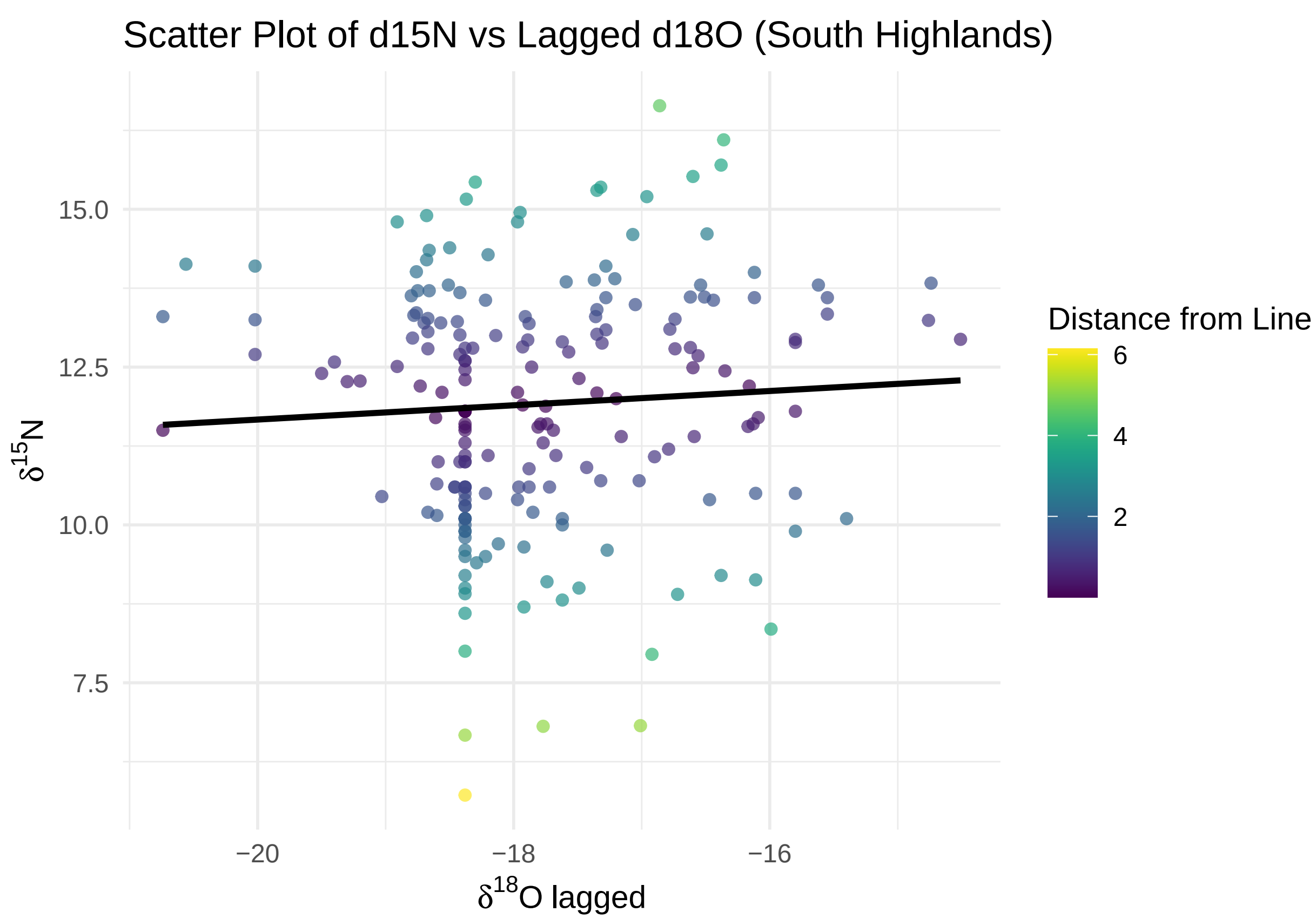

0.10 Section 4.3: d15N-quelccaya_d18O Nazca Dataset

This analysis explores the relationship between 15N values and the lagged 18O climate proxy in the Nazca Highlands region.

```
combined_data_lagged <- merge(Nazca.data, climate.d18O, by = "year.bp")
```

A Pearson correlation test is performed to determine if there is a significant linear relationship between 15N and lagged 18O.

```
correlation_test_lagged <- cor.test(combined_data_lagged$d15N,
                                    combined_data_lagged$lagged_d018)
print(correlation_test_lagged)
```

```
##
##  Pearson's product-moment correlation
##
## data:  combined_data_lagged$d15N and combined_data_lagged$lagged_dO18
## t = -0.24097, df = 143, p-value = 0.8099
## alternative hypothesis: true correlation is not equal to 0
## 95 percent confidence interval:
##  -0.1825562  0.1433335
## sample estimates:
##         cor
## -0.02014649
```

Next, a Generalized Additive Model (GAM) is applied to capture potential non-linear relationships between 15N and lagged 18O. GAMs are flexible models that can identify non-linear trends in the data by fitting smooth functions to the predictor variables. he smoothing function (s(lagged_dO18)) allows the model to capture non-linear patterns between 15N and 18O, providing insights into whether there are non-linear climate effects on nitrogen isotopes. The summary provides key information about the model fit, including the significance of the smooth term.

```r
gam_model_lagged_d18O <- gam(d15N ~ s(lagged_dO18), data = combined_data_lagged)
summary(gam_model_lagged_d18O)
```

```
##
## Family: gaussian
## Link function: identity
##
## Formula:
## d15N ~ s(lagged_dO18)
##
## Parametric coefficients:
##             Estimate Std. Error t value Pr(>|t|)
## (Intercept) 13.20966    0.08221   160.7   <2e-16 ***
## ---
## Signif. codes:  0 '***' 0.001 '**' 0.01 '*' 0.05 '.' 0.1 ' ' 1
##
## Approximate significance of smooth terms:
##                edf Ref.df     F p-value
## s(lagged_dO18)   1      1 0.058    0.81
##
## R-sq.(adj) =  -0.00658   Deviance explained = 0.0406%
## GCV = 0.99363  Scale est. = 0.97993   n = 145
```

To visualize the results of the GAM, the fitted values (predictions) and their associated standard errors are extracted from the model. A custom function is used to calculate the fitted values, along with the upper and lower confidence intervals (95% confidence interval), which are then used for plotting

```r
extract_gam_predictions <- function(gam_model) {
  pred_data <- data.frame(lagged_dO18 = gam_model$model$lagged_dO18)
  preds <- predict(gam_model, se.fit = TRUE)
  pred_data <- pred_data %>%
    mutate(fit = preds$fit,
           se.fit = preds$se.fit,
           lower = fit - 1.96 * se.fit,
```

```
           upper = fit + 1.96 * se.fit)
  return(pred_data)
}
pred_data <- extract_gam_predictions(gam_model_lagged_d18O)
```

The results of the GAM are visualized using ggplot2, with the fitted line representing the predicted relationship between 15N and lagged 18O, and the shaded ribbon showing the 95% confidence interval

```
ggplot() +
  geom_line(data = pred_data, aes(x = lagged_dO18, y = fit), color = "black",
            linewidth = 0.75) +
  geom_ribbon(data = pred_data, aes(x = lagged_dO18, ymin = lower,
                                    ymax = upper), fill = "#481668",
              alpha = 0.5) +
  theme_minimal() +
  labs(title = "GAM Fit for Lagged  18O and  15N",
       x = expression(delta^18 * O ~ "lagged"),
       y = expression(delta^{15}*N))
```

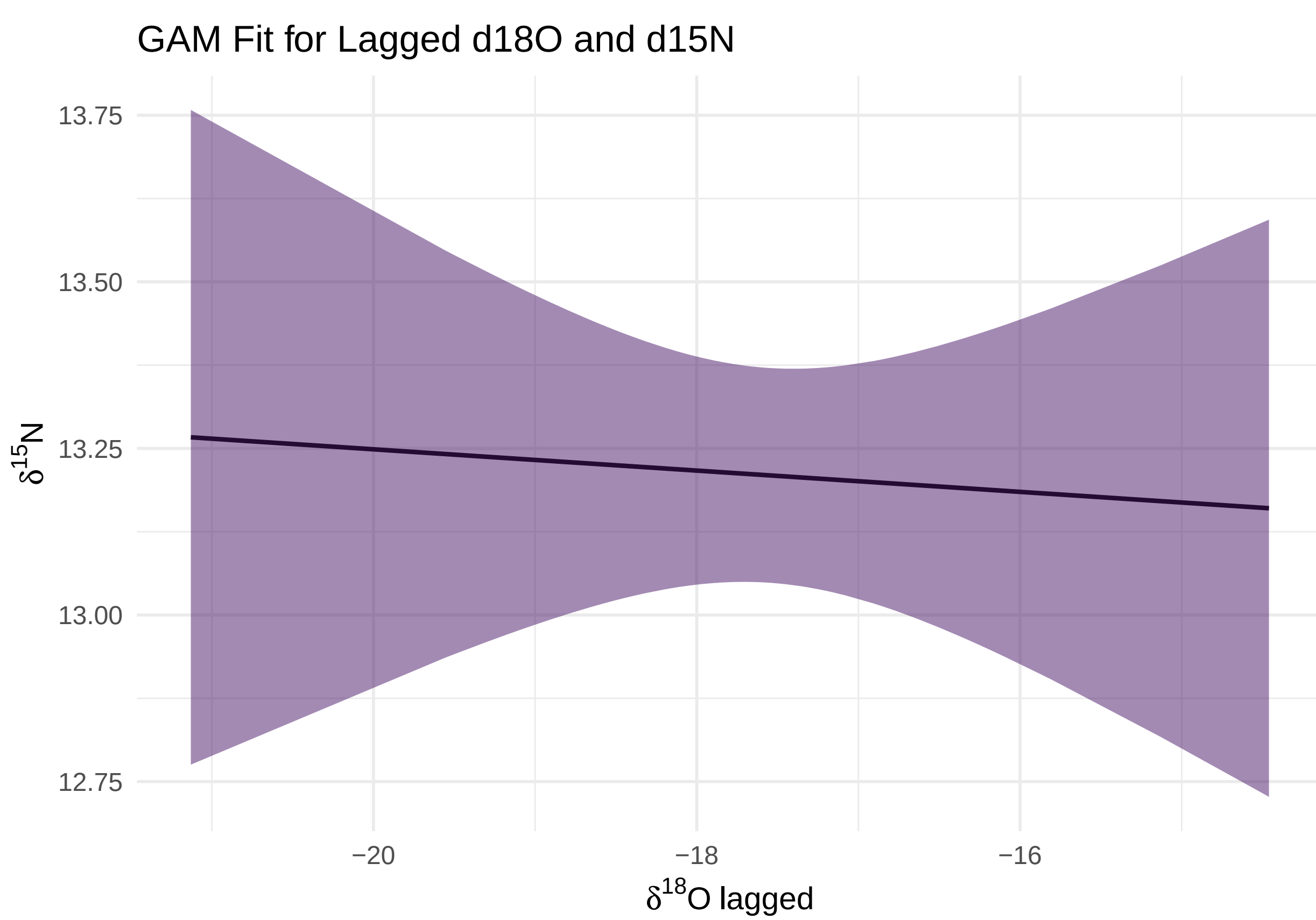

0.10.1 Driver 4: Influence of climate on d15N-Pollen

This analysis investigates the relationship between 15N values from human remains and pollen data in the Andes region which is used as a climate proxy, representing periods of high and low precipitation, which may have influenced nitrogen isotope levels in human remains through environmental impacts on soil and

plant isotopic compositions. This analysis uses a Monte Carlo simulation to explore potential correlations over multiple iterations, followed by a linear model to assess the relationship in more detail.

0.11 Section 4.4: Cerro Llamoca core Full Andes Analysis

Simulating Random Years for Human Data: To account for uncertainty in the dating of human remains, a random year is simulated for each individual within their known Start and End BP (Before Present) range. The simulate_one_round function is used to generate these simulated years

```
simulate_one_round <- function(start_bp, end_bp) {
  if (!is.na(start_bp) && !is.na(end_bp)) {
    return(round(runif(1, min = end_bp, max = start_bp)))
  } else {
    return(NA)
  }
}
```

The find_nearest_Mn_Fe_ratio function matches the simulated year for each human remain to the nearest Mn/Fe ratio in the climate pollen dataset, allowing for the comparison of 15N values to climate data

```
find_nearest_Mn_Fe_ratio <- function(year, climate.pollen) {
  nearest_year_index <- which.min(abs(climate.pollen$year.bp - year))
  return(climate.pollen$Mn_Fe_ratio[nearest_year_index])
}
```

A Monte Carlo simulation is performed to assess the correlation between 15N values and the Mn/Fe ratio over 10,000 iterations. For each iteration, new simulated years are generated, the nearest Mn/Fe ratio is identified, and the correlation between 15N and the Mn/Fe ratio is computed

```
simulate_correlation <- function(Human.LIP, climate.pollen, iterations = 10000) {
  correlation_results <- numeric(iterations)
  for (i in 1:iterations) {
    Human.LIP$SimulatedYearBP <- mapply(simulate_one_round,
                                        Human.LIP$Start.BP, Human.LIP$End.BP)
    Human.LIP$Nearest_Mn_Fe_ratio <-
      sapply(Human.LIP$SimulatedYearBP, find_nearest_Mn_Fe_ratio,
             climate.pollen = climate.pollen)
    valid_indices <-
      !is.na(Human.LIP$Nearest_Mn_Fe_ratio) & !is.na(Human.LIP$d15N)
    correlation_results[i] <- cor(Human.LIP$d15N[valid_indices],
                                  Human.LIP$Nearest_Mn_Fe_ratio[valid_indices])
  }
  return(correlation_results)
}
```

Before running the correlation analysis, the Mn/Fe ratio is calculated for the pollen dataset by dividing the counts per second (cps) values of Mn by those of Fe

```
climate.pollen$Mn_Fe_ratio <- climate.pollen$Mn_cps / climate.pollen$Fe_cps
```

After running the Monte Carlo simulation, the results are summarized and visualized. The distribution of correlation coefficients is plotted, and the percentages of positive and negative correlations are computed

```r
cat("Full Andes Region Analysis\n")
```

```
## Full Andes Region Analysis
```

```r
correlation_results <- simulate_correlation(Human.LIP, climate.pollen)
summary_statistics <- summary(correlation_results)
hist(correlation_results, breaks = 50,
     main = "Distribution of Mn/Fe Correlation Coefficients",
     xlab = "Correlation Coefficient")
```

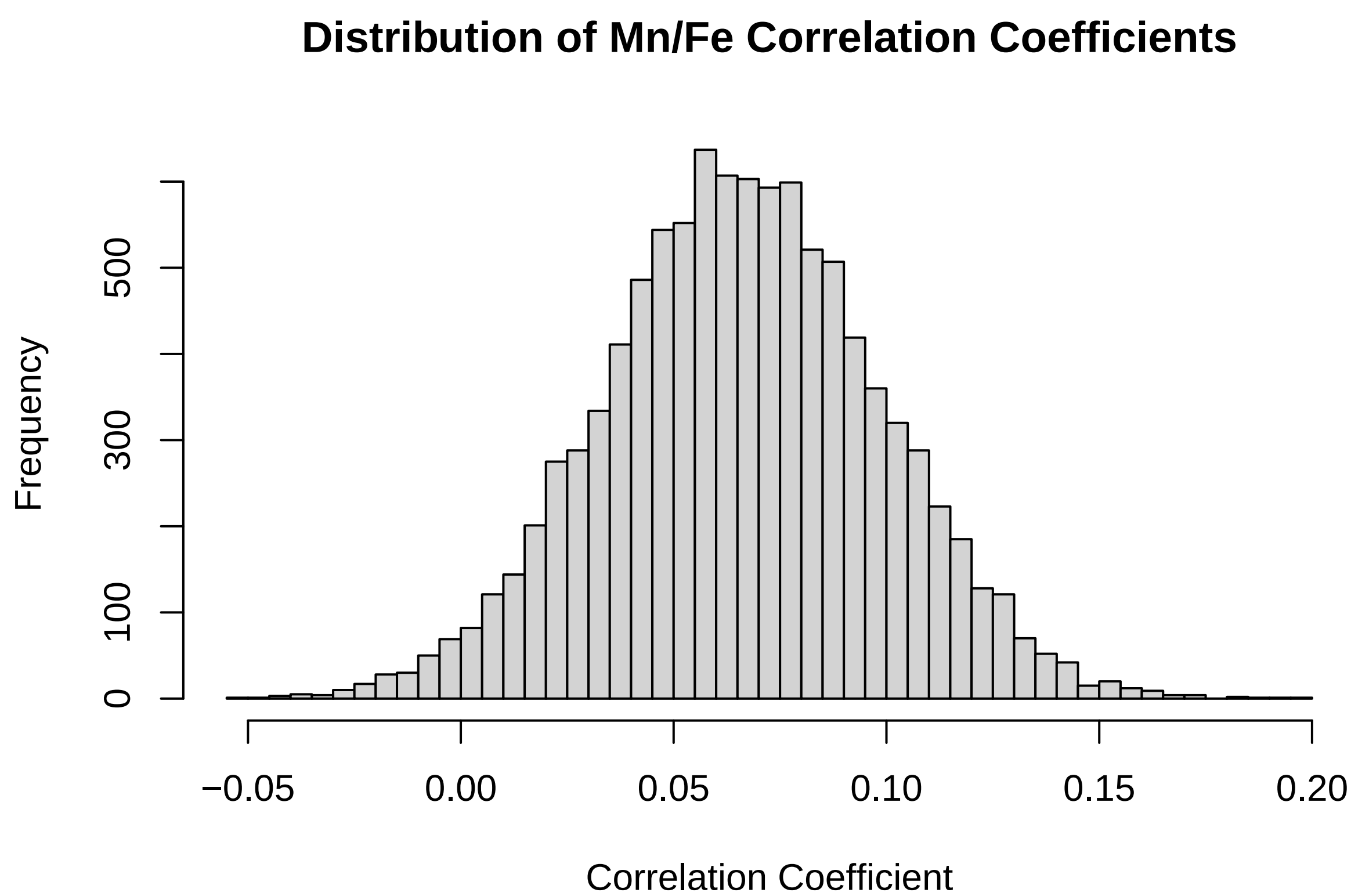


```r
percentage_positive_correlation <-
  sum(correlation_results > 0) / length(correlation_results) * 100
percentage_negative_correlation <-
  sum(correlation_results < 0) / length(correlation_results) * 100
print(summary_statistics)
```

```
##     Min.  1st Qu.   Median     Mean  3rd Qu.     Max.
## -0.05018  0.04446  0.06586  0.06605  0.08764  0.19617
```

```r
print(paste("Percentage of positive correlations:",
            percentage_positive_correlation))
```

```
## [1] "Percentage of positive correlations: 97.82"
```

```r
print(paste("Percentage of negative correlations:",
            percentage_negative_correlation))
```

```
## [1] "Percentage of negative correlations: 2.18"
```

```r
plot(density(correlation_results),
     main = "Density of Mn/Fe Correlation Coefficients",
     xlab = "Correlation Coefficient", ylab = "Density")
```

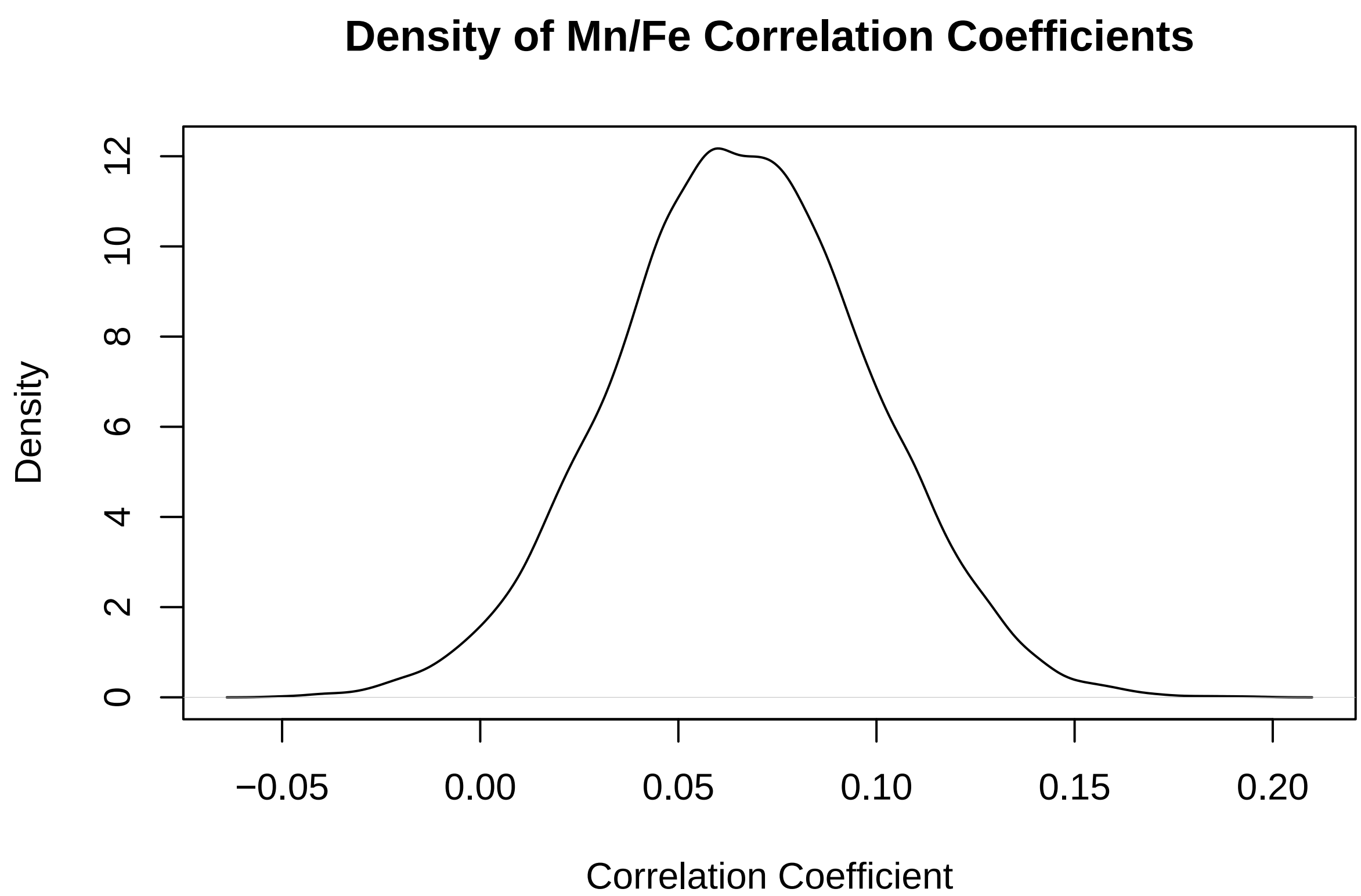

A one-sample t-test is conducted to determine if the mean correlation coefficient is significantly different from zero. Additionally, a 95% confidence interval for the correlation coefficients is calculated

```r
t_test_results <- t.test(correlation_results, mu = 0)
print(t_test_results)
```

```
##
##  One Sample t-test
##
## data:  correlation_results
## t = 203.56, df = 9999, p-value < 2.2e-16
## alternative hypothesis: true mean is not equal to 0
## 95 percent confidence interval:
##  0.06541156 0.06668357
## sample estimates:
##  mean of x
## 0.06604756
```

```
conf_int <- quantile(correlation_results, probs = c(0.025, 0.975))
print(paste("95% confidence interval for the Mn/Fe correlation coefficient:",
            conf_int[1], "to", conf_int[2]))
```

```
## [1] "95% confidence interval for the Mn/Fe correlation coefficient: 0.00223281045706396 to 0.1292481
```

A linear model is fitted to the filtered data, examining the relationship between 15N and the Mn/Fe ratio. Outliers with a Mn/Fe ratio greater than 1 are removed to avoid skewing the analysis. The residuals (absolute differences between observed and predicted values) are also calculated, and a scatter plot is generated to visualize the relationship between 15N and the Mn/Fe ratio.

```
Human.LIP$SimulatedYearBP <-
  mapply(simulate_one_round, Human.LIP$Start.BP, Human.LIP$End.BP)
Human.LIP$Nearest_Mn_Fe_ratio <- sapply(Human.LIP$SimulatedYearBP,
                                        find_nearest_Mn_Fe_ratio,
                                        climate.pollen = climate.pollen)
Human.LIP$Nearest_Mn_Fe_ratio <- as.numeric(Human.LIP$Nearest_Mn_Fe_ratio)
filtered_data <-
  Human.LIP[!is.na(Human.LIP$d15N) & !is.na(Human.LIP$Nearest_Mn_Fe_ratio), ]
model <- lm(d15N ~ Nearest_Mn_Fe_ratio, data = filtered_data)
filtered_data$residuals <- abs(resid(model))
summary(filtered_data$Nearest_Mn_Fe_ratio)
```

```
##     Min.  1st Qu.   Median     Mean  3rd Qu.     Max.
## 0.000000 0.001750 0.005504 0.087554 0.011153 1.000000
```

```
filtered_data_no_outliers <-
  filtered_data[filtered_data$Nearest_Mn_Fe_ratio < 1, ]
model_no_outliers <-
  lm(d15N ~ Nearest_Mn_Fe_ratio, data = filtered_data_no_outliers)
filtered_data_no_outliers$residuals <- abs(resid(model_no_outliers))
```

A scatter plot is created to visualize the linear relationship between 15N and the Mn/Fe ratio, with residuals (distance from the regression line) represented by color

```
ggplot(filtered_data_no_outliers, aes(x = Nearest_Mn_Fe_ratio, y = d15N,
                                      color = residuals)) +
  geom_point(alpha = 0.7) +
  geom_smooth(method = 'lm', color = 'black', se = FALSE) +
  labs(title = "Scatter Plot of  15N vs % Poaceae (No Outliers)",
       x = "% Poaceae",
       y = expression(delta^{15}*N),
       color = "Distance from Line") +
  scale_color_viridis(option = "viridis") +
  theme_minimal()
```

```
## `geom_smooth()` using formula = 'y ~ x'
```

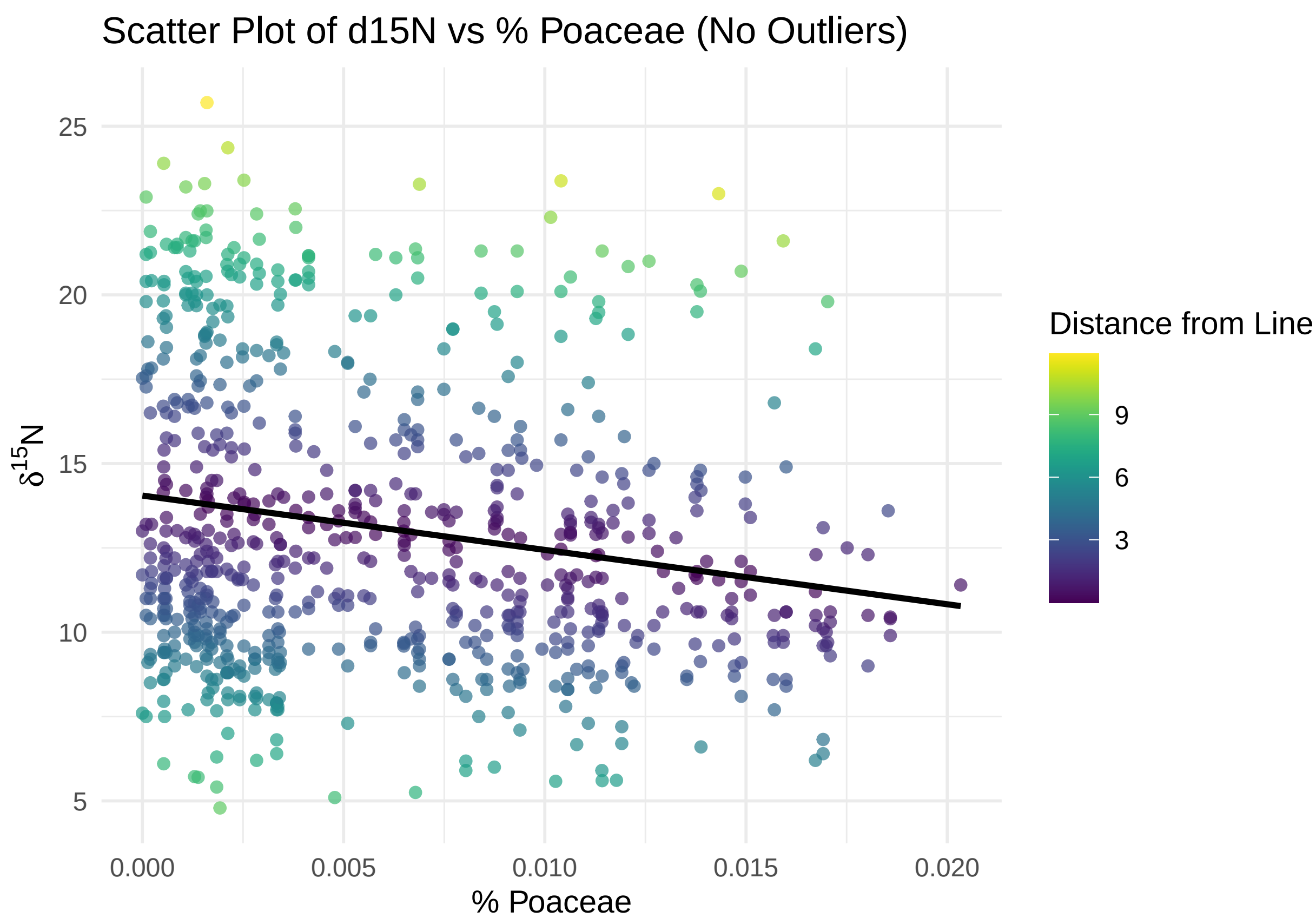

0.12 Section 4.5: Cerro Llamoca coreSouth Highlands Subset Analysis

This analysis focuses on the South Highlands region to explore the relationship between 15N values from human remains and the Mn/Fe ratio derived from pollen data. The Mn/Fe ratio is used as a proxy for climate conditions (such as precipitation) and is examined here to assess how climate may have influenced nitrogen isotope levels in the South Highlands. The Monte Carlo simulation generates a distribution of correlation coefficients, followed by a linear model and visualization to further explore the relationship.

```
cat("\nSouth Highlands Subset Analysis\n")
```

```
##
## South Highlands Subset Analysis
```

```
SouthernHighlands <- subset(Human.LIP, Region == "South Highlands")
```

A Monte Carlo simulation is performed to assess the correlation between 15N values and the Mn/Fe ratio in the South Highlands region. The function simulate_correlation runs 10,000 iterations, simulating random years for the human remains, finding the corresponding Mn/Fe ratio from the pollen data, and calculating the correlation between 15N and Mn/Fe ratio. This generates a distribution of correlation coefficients, which provides a robust measure of how 15N and the Mn/Fe ratio correlate in the South Highlands over multiple simulations.

```r
correlation_results_SouthHighlands <- simulate_correlation(SouthernHighlands,
                                                           climate.pollen)
```

After running the Monte Carlo simulation, summary statistics are computed, and a histogram is generated to visualize the distribution of correlation coefficients. The percentages of positive and negative correlations are also calculated to assess the overall trend

```r
summary_statistics_SouthHighlands <- summary(correlation_results_SouthHighlands)
#The results are visualized using a histogram to show the distribution of correlation coefficients
hist(correlation_results_SouthHighlands, breaks = 50,
     main = "Distribution of Mn/Fe Correlation Coefficients (South Highlands)",
     xlab = "Correlation Coefficient")
```

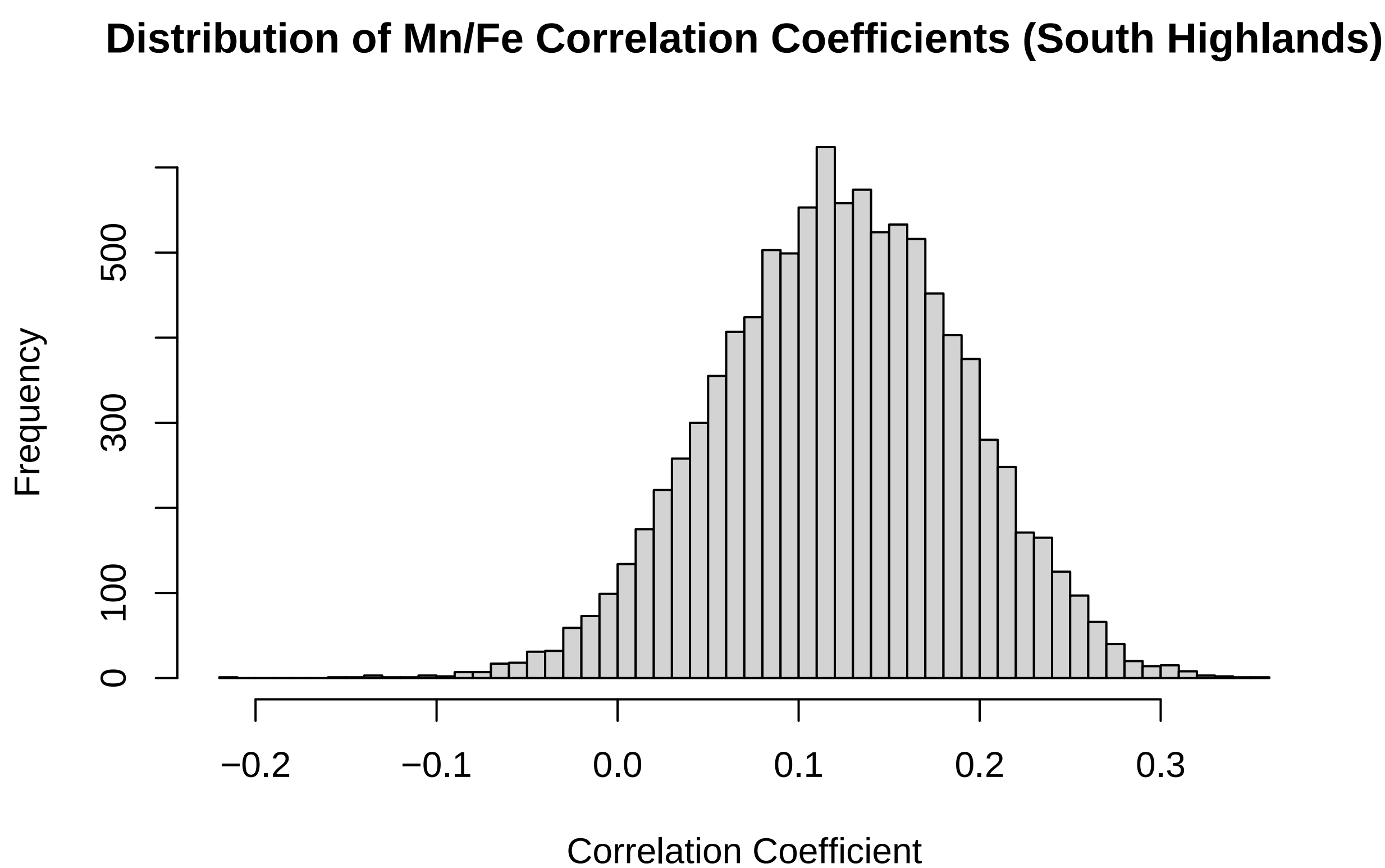


```r
percentage_positive_correlation_SouthHighlands <-
  sum(correlation_results_SouthHighlands > 0) /
  length(correlation_results_SouthHighlands) * 100
percentage_negative_correlation_SouthHighlands <-
  sum(correlation_results_SouthHighlands < 0) /
  length(correlation_results_SouthHighlands) * 100
print(summary_statistics_SouthHighlands)
```

```
##     Min.  1st Qu.   Median     Mean  3rd Qu.     Max.
## -0.21220  0.07711  0.12375  0.12273  0.16968  0.35740
```

```r
print(paste("Percentage of positive correlations:",
            percentage_positive_correlation_SouthHighlands))
```

```
## [1] "Percentage of positive correlations: 96.44"
```

```r
print(paste("Percentage of negative correlations:",
            percentage_negative_correlation_SouthHighlands))
```

```
## [1] "Percentage of negative correlations: 3.56"
```

```r
plot(density(correlation_results_SouthHighlands),
     main = "Density of Mn/Fe Correlation Coefficients (South Highlands)",
     xlab = "Correlation Coefficient", ylab = "Density")
```

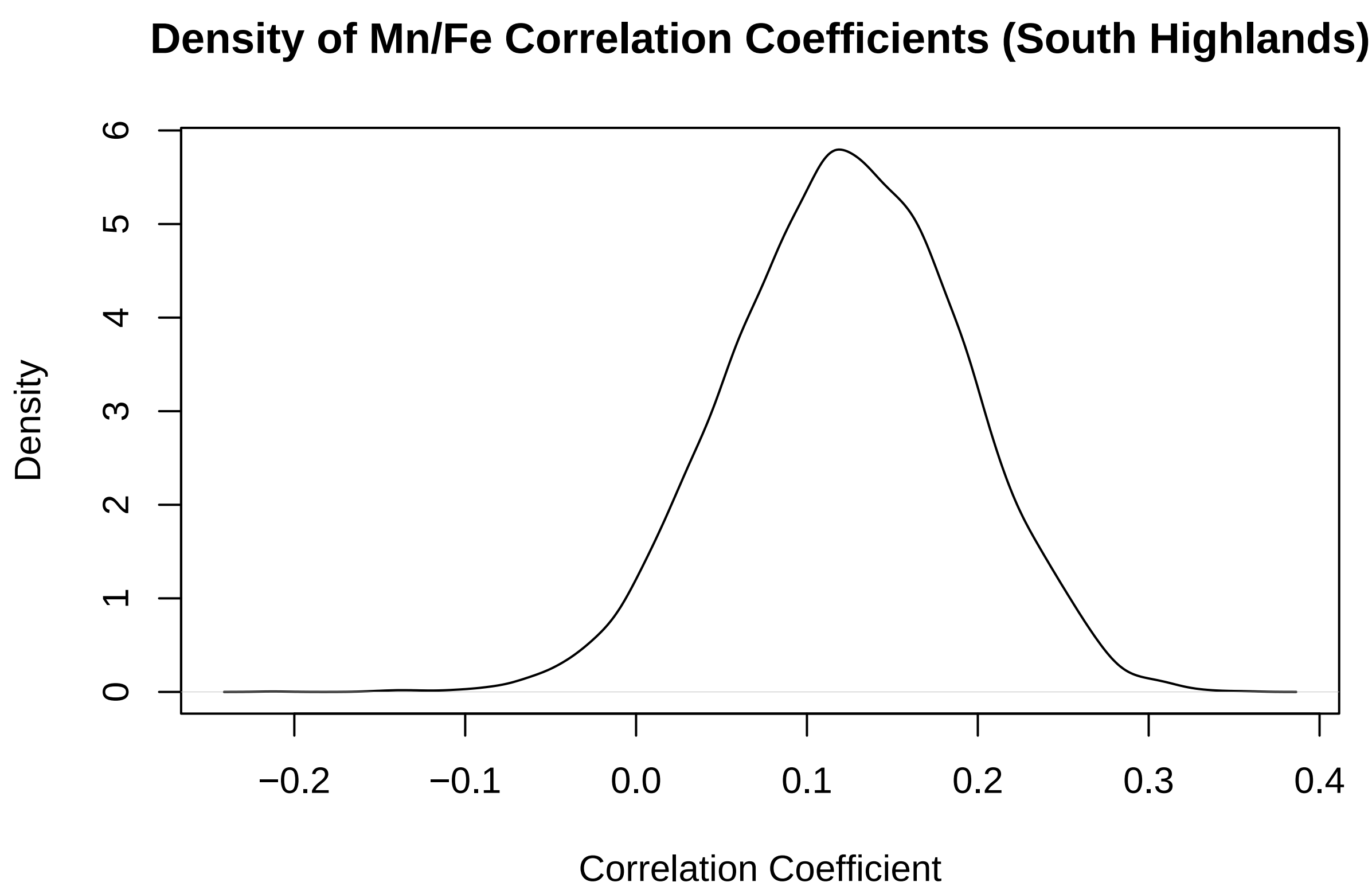

A one-sample t-test is performed to assess whether the mean correlation coefficient is significantly different from zero. Additionally, a 95% confidence interval is calculated to provide a statistical range for the correlation coefficients. This statistical testing provides evidence on whether there is a significant relationship between 15N and the Mn/Fe ratio in the South Highlands.

```r
t_test_results_SouthHighlands <- t.test(correlation_results_SouthHighlands,
                                        mu = 0)
print(t_test_results_SouthHighlands)
```

```
##
```

```
##  One Sample t-test
##
## data:  correlation_results_SouthHighlands
## t = 181.31, df = 9999, p-value < 2.2e-16
## alternative hypothesis: true mean is not equal to 0
## 95 percent confidence interval:
##  0.1214030 0.1240567
## sample estimates:
## mean of x
## 0.1227298
```

```
conf_int_SouthHighlands <- quantile(correlation_results_SouthHighlands,
                                    probs = c(0.025, 0.975))
print(paste("95% confidence interval for correlation coefficient (South Highlands):",
            conf_int_SouthHighlands[1], "to", conf_int_SouthHighlands[2]))
```

```
## [1] "95% confidence interval for correlation coefficient (South Highlands): -0.0112976161012464 to 0
```

A linear model is fitted to explore the relationship between 15N and the Mn/Fe ratio in the South Highlands subset. To ensure accurate modeling, outliers with Mn/Fe ratios greater than 1 are removed from the dataset before fitting the model

```
SouthernHighlands$SimulatedYearBP <-
  mapply(simulate_one_round, SouthernHighlands$Start.BP, SouthernHighlands$End.BP)
SouthernHighlands$Nearest_Mn_Fe_ratio <-
  sapply(SouthernHighlands$SimulatedYearBP, find_nearest_Mn_Fe_ratio,
         climate.pollen = climate.pollen)
SouthernHighlands$Nearest_Mn_Fe_ratio <-
  as.numeric(SouthernHighlands$Nearest_Mn_Fe_ratio)
filtered_data_south <-
  SouthernHighlands[!is.na(SouthernHighlands$d15N) &
                    !is.na(SouthernHighlands$Nearest_Mn_Fe_ratio), ]
model_south <- lm(d15N ~ Nearest_Mn_Fe_ratio, data = filtered_data_south)
filtered_data_south$residuals <- abs(resid(model_south))
summary(filtered_data_south$Nearest_Mn_Fe_ratio)
```

```
##      Min.   1st Qu.    Median      Mean   3rd Qu.      Max.
## 0.0000918 0.0033599 0.0087469 0.1251895 0.0126121 1.0000000
```

```
filtered_data_south_no_outliers <-
  filtered_data_south[filtered_data_south$Nearest_Mn_Fe_ratio < 1, ]
model_south_no_outliers <-
  lm(d15N ~ Nearest_Mn_Fe_ratio, data = filtered_data_south_no_outliers)
filtered_data_south_no_outliers$residuals <- abs(resid(model_south_no_outliers))
```

A scatter plot is generated to visualize the relationship between 15N and the Mn/Fe ratio in the South Highlands, with the linear model fit overlaid as a black line. Residuals are represented by color, indicating the distance of each data point from the fitted line

```
ggplot(filtered_data_south_no_outliers, aes(x = Nearest_Mn_Fe_ratio, y = d15N,
                                            color = residuals)) +
  geom_point(alpha = 0.7) +
```

```
  geom_smooth(method = 'lm', color = 'black', se = FALSE) +
  labs(title = "Scatter Plot of  15N vs % Poaceae (South Highlands)",
       x = "% Poaceae",
       y = expression(delta^{15}*N),
       color = "Distance from Line") +
  scale_color_viridis(option = "viridis") +
  xlim(0, 0.02) +  # Set x-axis limits
  ylim(5.0, 25.0) +  # Set y-axis limits
  theme_minimal()
```

```
## `geom_smooth()` using formula = 'y ~ x'
```

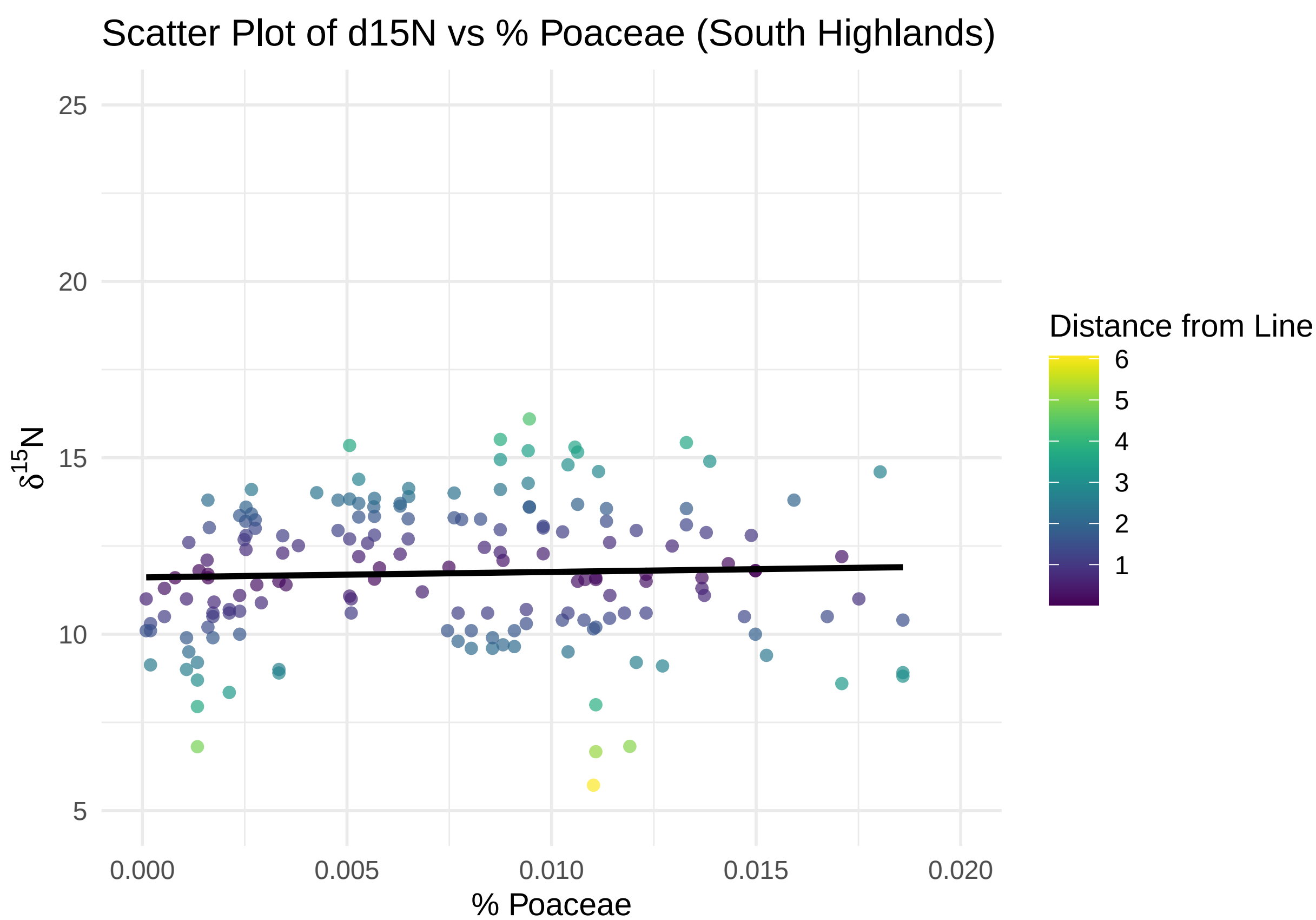

0.13 Section 4.6: Cerro Llamoca core Nazca Analysis

Merging the Nazca Dataset with Climate Pollen Data: The human isotopic dataset (Nazca.data) is merged with the climate pollen dataset (climate.pollen) based on the year.bp variable, which represents the years Before Present (BP). This step allows the analysis to align 15N values from human remains with the corresponding Mn/Fe ratio from the climate data. After merging, missing values are removed to ensure complete cases for analysis

```
cat("\nNazca Region Analysis\n")
```

```
##
## Nazca Region Analysis
```

```r
combined_data <- merge(Nazca.data, climate.pollen, by = "year.bp")
#combined_data <- na.omit(combined_data)
```

The Mn/Fe ratio is calculated by dividing the counts per second (cps) values of manganese (Mn) by those of iron (Fe) in the pollen dataset. This ratio serves as a proxy for precipitation and other climate factors in the region

```r
combined_data$Mn_Fe_ratio <- combined_data$Mn_cps / combined_data$Fe_cps
```

A Pearson correlation test is performed to assess the strength and direction of the linear relationship between 15N values and the Mn/Fe ratio. This test provides a correlation coefficient and a p-value to determine whether the relationship is statistically significant

```r
correlation_test <- cor.test(combined_data$d15N, combined_data$Mn_Fe_ratio)
print(correlation_test)
```

```
##
##  Pearson's product-moment correlation
##
## data:  combined_data$d15N and combined_data$Mn_Fe_ratio
## t = -1.0423, df = 23, p-value = 0.3081
## alternative hypothesis: true correlation is not equal to 0
## 95 percent confidence interval:
##  -0.5604699  0.1995016
## sample estimates:
##        cor
## -0.2123703
```

To explore potential nonlinear relationships between 15N and the Mn/Fe ratio, a Generalized Additive Model (GAM) is fitted. GAMs allow for the detection of non-linear trends by fitting a smooth curve to the data. Here, the smoothing function (s) is applied to the Mn/Fe ratio with a parameter (k = 4) that controls the flexibility of the smooth. The GAM is used to assess whether there is a non-linear relationship between 15N and the Mn/Fe ratio. The summary of the model provides details on the significance of the smooth term and how well the model fits the data.

```r
gam_model_Mn_Fe_ratio <- gam(d15N ~ s(Mn_Fe_ratio, k = 4), data = combined_data)
summary(gam_model_Mn_Fe_ratio)
```

```
##
## Family: gaussian
## Link function: identity
##
## Formula:
## d15N ~ s(Mn_Fe_ratio, k = 4)
##
## Parametric coefficients:
##             Estimate Std. Error t value Pr(>|t|)
## (Intercept)   13.320      0.189   70.49   <2e-16 ***
## ---
## Signif. codes:  0 '***' 0.001 '**' 0.01 '*' 0.05 '.' 0.1 ' ' 1
##
```

```
## Approximate significance of smooth terms:
##                  edf Ref.df     F p-value
## s(Mn_Fe_ratio)   1      1 1.086   0.308
##
## R-sq.(adj) =  0.00358   Deviance explained = 4.51%
## GCV = 0.97024  Scale est. = 0.89262   n = 25
```

To visualize the results of the GAM, the predicted 15N values and their corresponding 95% confidence intervals are extracted from the model. A custom function is used to calculate the fitted values along with their standard errors, which are then used to construct the confidence intervals

```r
extract_gam_predictions <- function(gam_model) {
  pred_data <- data.frame(Mn_Fe_ratio = gam_model$model$Mn_Fe_ratio)
  preds <- predict(gam_model, se.fit = TRUE)
  pred_data <- pred_data %>%
    mutate(fit = preds$fit,
           se.fit = preds$se.fit,
           lower = fit - 1.96 * se.fit,
           upper = fit + 1.96 * se.fit)
  return(pred_data)
}
pred_data <- extract_gam_predictions(gam_model_Mn_Fe_ratio)
```

A plot is generated using ggplot2 to visualize the GAM results. The fitted line shows the predicted relationship between the Mn/Fe ratio and 15N, while the shaded ribbon represents the 95% confidence interval around the predictions

```r
ggplot(pred_data, aes(x = Mn_Fe_ratio)) +
  geom_line(aes(y = fit), color = "black", linewidth = 0.75) +
  geom_ribbon(aes(ymin = lower, ymax = upper), fill = "#481668", alpha = 0.5) + #
  theme_minimal() +
  labs(title = "GAM Fit for % Poaceae and  15N",
       x = "% Poaceae",
       y = expression(delta^{15}*N)) +
  ylim(min(pred_data$lower), max(pred_data$upper))
```

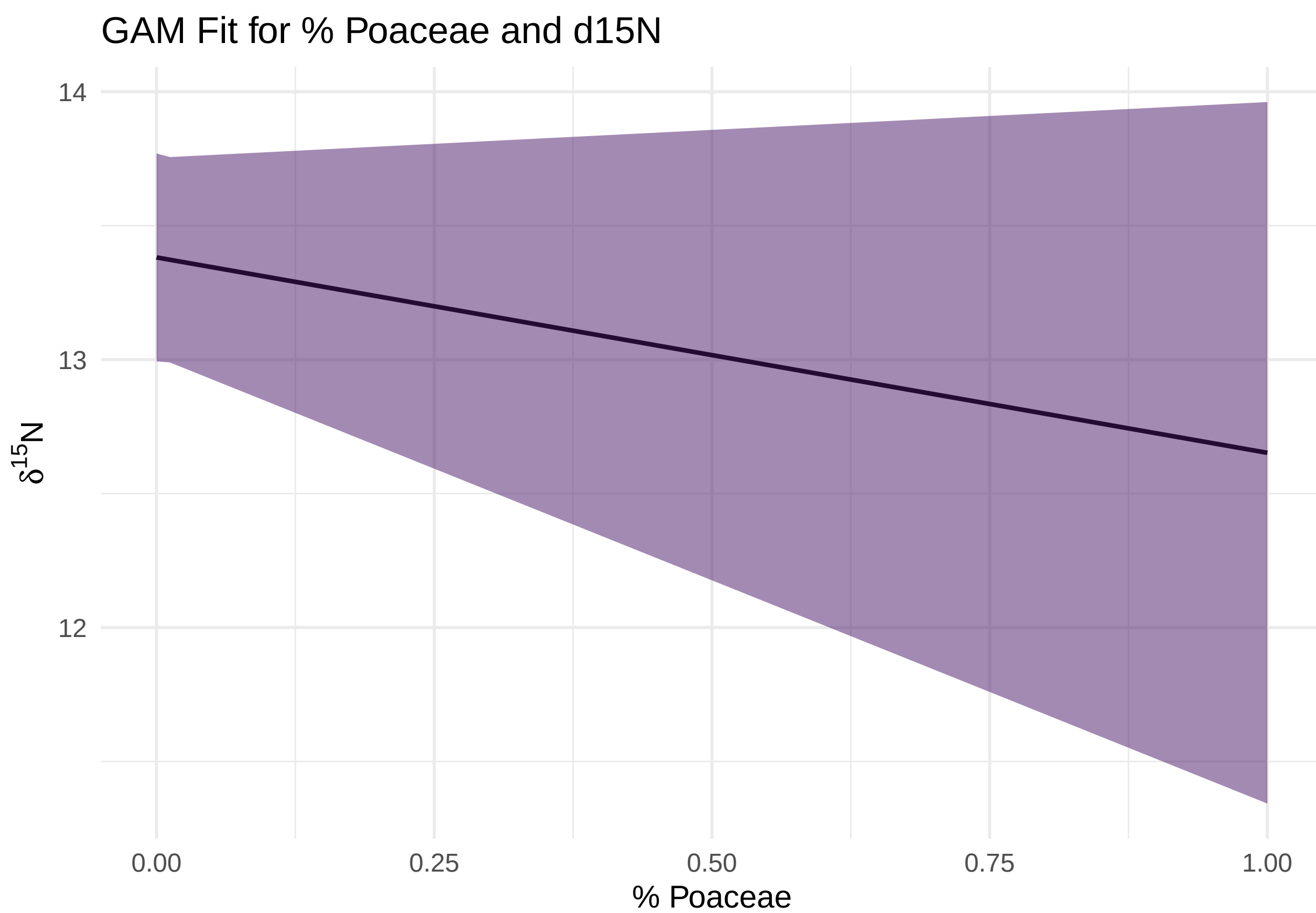

0.14 Section 5: Change in dN15 through time

This analysis examines how 15N values vary across different Late Intermediate Period (LIP) phases (LIP1, LIP2, LIP3), age categories (Adults vs. Children), and sex in the Peruvian dataset. The statistical models and visualizations help determine whether 15N values show significant differences across these variables, providing insights into dietary patterns, age-specific differences, and potential sex-based variations.

First are tables with the summarized demographic data

```
sex_site_table <- Nazca.data %>%
  group_by(site, sex) %>%
  summarise(count = n(), .groups = "drop") %>%
  pivot_wider(names_from = sex, values_from = count, values_fill = list(count = 0))
print(sex_site_table)
```

```
## # A tibble: 3 x 4
##   site          Female   Ind  Male
##   <chr>          <int> <int> <int>
## 1 Ayaorcco          21     8    25
## 2 Ayapata           32    11    24
## 3 C. Tribolpata     16     5     8
```

```
sex_age_site_table <- Nazca.data %>%
    group_by(site, sex, age.categ) %>%
```

```
    summarise(count = n(), .groups = "drop") %>%
    pivot_wider(names_from = age.categ, values_from = count, values_fill = list(count = 0))
sex_age_site_table <- sex_age_site_table %>%
    mutate(Total = rowSums(across(where(is.numeric))))
column_totals <- sex_age_site_table %>%
    summarise(across(where(is.numeric), sum)) %>%
    mutate(site = "Total", sex = "Total")
final_table <- bind_rows(sex_age_site_table, column_totals)
print(final_table)
```

```
## # A tibble: 10 x 8
##    site    sex   Adolescent `Middle Adult` `Young Adult` Child `Old Adult` Total
##    <chr>   <chr>      <int>          <int>         <int> <int>       <int> <dbl>
##  1 Ayaorc~ Fema~          3              4            14     0           0    21
##  2 Ayaorc~ Ind            1              5             0     1           1     8
##  3 Ayaorc~ Male           5              8             5     0           7    25
##  4 Ayapata Fema~         11             12             9     0           0    32
##  5 Ayapata Ind            3              0             0     7           1    11
##  6 Ayapata Male           0             10            10     0           4    24
##  7 C. Tri~ Fema~          4              5             7     0           0    16
##  8 C. Tri~ Ind            0              3             0     2           0     5
##  9 C. Tri~ Male           0              4             0     0           4     8
## 10 Total   Total         27             51            45    10          17   150
```

The first part of the analysis examines the relationship between 15N values and the different LIP phases (LIP1, LIP2, LIP3). A linear model (lm) is fitted to assess whether 15N values differ significantly across these phases, which represent temporal divisions within the Late Intermediate Period.The results indicated no statistically significant differences between the phases, with the model showing a p-value of 0.128.

```
Nazca.LIP$Phase <- factor(Nazca.LIP$Phase, levels = c("LIP1", "LIP2", "LIP3"))
lm_model_phase <- lm(d15N ~ Phase, data = Nazca.LIP)
summary(lm_model_phase)
```

```
##
## Call:
## lm(formula = d15N ~ Phase, data = Nazca.LIP)
##
## Residuals:
##     Min      1Q  Median      3Q     Max
## -3.5657 -0.4192  0.0640  0.5808  1.8640
##
## Coefficients:
##             Estimate Std. Error t value Pr(>|t|)
## (Intercept)  13.4360     0.1387  96.863   <2e-16 ***
## PhaseLIP2    -0.2168     0.1943  -1.116    0.267
## PhaseLIP3    -0.3703     0.2162  -1.713    0.089 .
## ---
## Signif. codes:  0 '***' 0.001 '**' 0.01 '*' 0.05 '.' 0.1 ' ' 1
##
## Residual standard error: 0.9808 on 134 degrees of freedom
##   (3 observations deleted due to missingness)
## Multiple R-squared:  0.02242,    Adjusted R-squared:  0.007827
## F-statistic: 1.536 on 2 and 134 DF,  p-value: 0.2189
```

Estimated marginal means (EMMs) are calculated to explore the average 15N values for each LIP phase. This result suggests no strong temporal change in ^{1}N values across the phases. The estimated marginal means (EMMs) confirmed similar average ^{1}N values for LIP1 (13.4‰), LIP2 (13.4‰), and LIP3 (13.1‰).

```
emm_phase <- emmeans(lm_model_phase, ~ Phase)
print(emm_phase)
```

```
##  Phase emmean    SE  df lower.CL upper.CL
##  LIP1    13.4 0.139 134     13.2     13.7
##  LIP2    13.2 0.136 134     13.0     13.5
##  LIP3    13.1 0.166 134     12.7     13.4
##
## Confidence level used: 0.95
```

Since 15N may not follow a normal distribution, a Kruskal-Wallis test is performed to assess whether there are significant differences in 15N values across LIP phases. If the test is significant, pairwise comparisons are conducted using the Wilcoxon rank-sum test, adjusted with the Bonferroni correction.

```
kruskal_result <- kruskal.test(d15N ~ Phase, data = Nazca.LIP)
print(kruskal_result)
```

```
##
##  Kruskal-Wallis rank sum test
##
## data:  d15N by Phase
## Kruskal-Wallis chi-squared = 3.3556, df = 2, p-value = 0.1868
```

```
if (kruskal_result$p.value < 0.05) {
  pairwise_wilcox_result <- pairwise.wilcox.test(Peru$d15N, Peru$Phase,
                                                  p.adjust.method = "bonferroni")
  print(pairwise_wilcox_result)
}
```

Two plots are created to visualize 15N distributions over time and across phase, boxplot of 15N values by LIP phase.

```
ggplot(Nazca.LIP, aes(x = Phase, y = d15N, fill = Phase)) +
  geom_boxplot(position = position_dodge(0.9)) +
  labs(x = "Phase", y = expression(delta^{15}*N ~ "\u2030"))+
  scale_fill_viridis_d(option = "viridis") +
  theme_minimal() +
  theme(legend.position = "none")
```

```
## Warning: Removed 3 rows containing non-finite outside the scale range
## (`stat_boxplot()`).
```

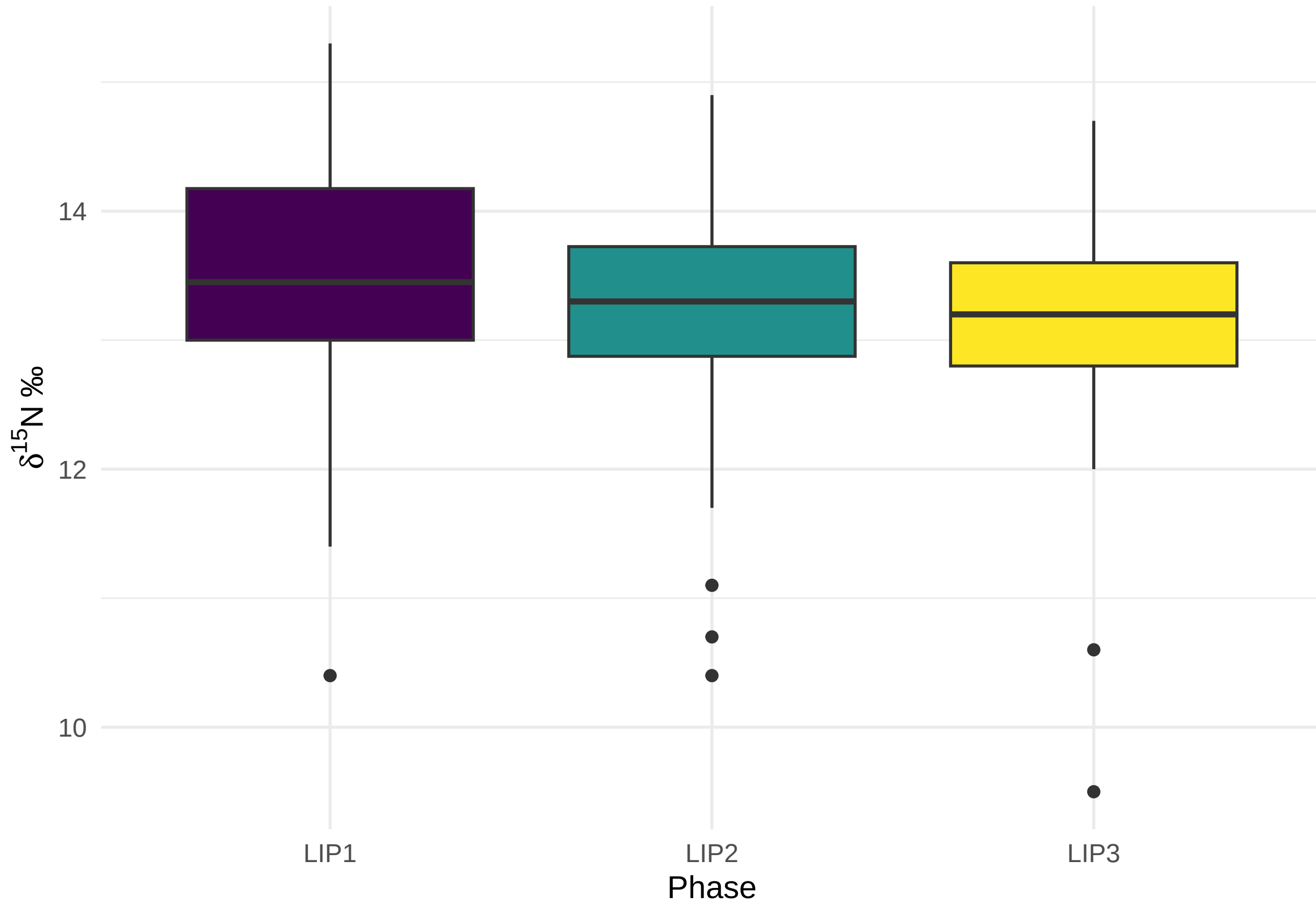

To visualize the overall trend of 15N values through time, a scatter plot is created, with 15N values plotted against the year in CE (Common Era). The color gradient reflects the 15N values

```
ggplot(data = Nazca.LIP, aes(x = year.CE, y = d15N, color = d15N)) +
  geom_point() +
  labs(x = "Year (CE)", y = expression(delta^{15}*N ~ "\u2030"))+
  theme_minimal() +
  scale_color_gradient(low = "#313375", high = "#8FB2EF") +
  theme(legend.position = "none")
```

```
## Warning: Removed 3 rows containing missing values or values outside the scale range
## (`geom_point()`).
```

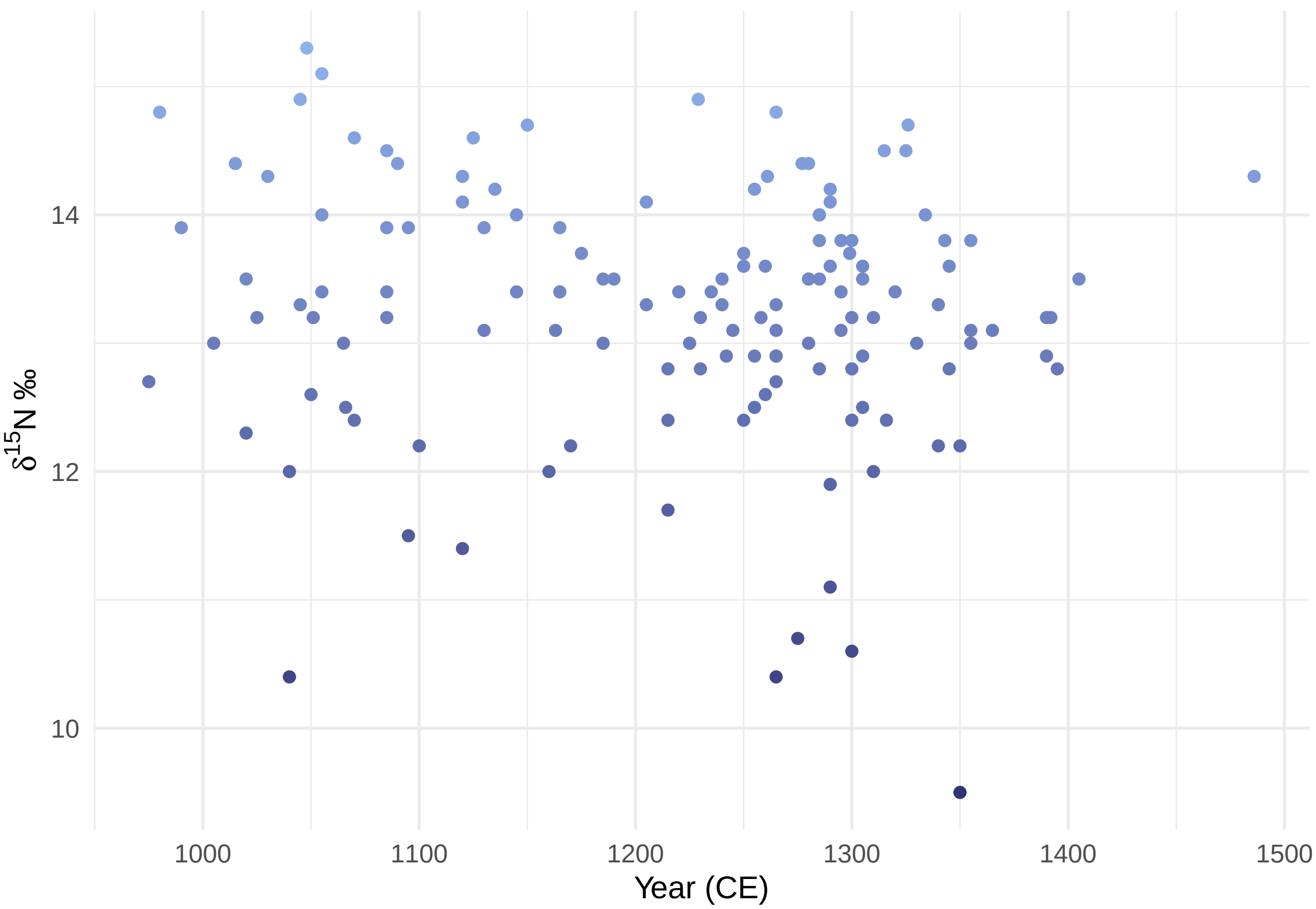

The next section of the analysis investigates whether 15N values differ between adults and children. A linear model is fitted, and EMMs are calculated for each age category

```
Nazca.LIP$age.categ2 <- factor(Nazca.LIP$age.categ2,
                               levels = c("Adult", "Child"))
```

The linear model indicated no significant difference in ^{1}N values between these two age categories, with a p-value of 0.31. The EMMs for adults showed an average ^{1}N of 13.4‰, while children had a slightly lower average of 13.0‰, though this difference was not statistically significant.

```
lm_model_age.categ2 <- lm(d15N ~ age.categ2, data = Nazca.LIP)
summary(lm_model_age.categ2)
```

```
##
## Call:
## lm(formula = d15N ~ age.categ2, data = Nazca.LIP)
##
## Residuals:
##     Min      1Q  Median      3Q     Max
## -3.7977 -0.3977  0.1023  0.6023  2.0023
##
## Coefficients:
##                 Estimate Std. Error t value Pr(>|t|)
## (Intercept)     13.29766    0.08639  153.92   <2e-16 ***
## age.categ2Child -0.58655    0.33707   -1.74   0.0841 .
```

```
## ---
## Signif. codes:  0 '***' 0.001 '**' 0.01 '*' 0.05 '.' 0.1 ' ' 1
##
## Residual standard error: 0.9774 on 135 degrees of freedom
##   (3 observations deleted due to missingness)
## Multiple R-squared:  0.02194,    Adjusted R-squared:  0.01469
## F-statistic: 3.028 on 1 and 135 DF,  p-value: 0.08412
```

EMMs: These provide adjusted mean estimates for 15N by age category.

```r
emm_age.categ2 <- emmeans(lm_model_age.categ2, ~ age.categ2)
print(emm_age.categ2)
```

```
##  age.categ2 emmean     SE  df lower.CL upper.CL
##  Adult        13.3 0.0864 135     13.1     13.5
##  Child        12.7 0.3258 135     12.1     13.4
##
## Confidence level used: 0.95
```

A summary of the average 15N values for each age group is also calculated

```r
avg_d15N <- Nazca.LIP %>%
  group_by(age.categ2) %>%
  summarize(avg_d15N = mean(d15N, na.rm = TRUE))
print(avg_d15N)
```

```
## # A tibble: 2 x 2
##   age.categ2 avg_d15N
##   <fct>         <dbl>
## 1 Adult          13.3
## 2 Child          12.7
```

A boxplot is created to visualize 15N values by age category (Adults vs. Children):

```r
ggplot(Nazca.LIP, aes(x = age.categ2, y = d15N, fill = age.categ2)) +
  geom_boxplot() +
  labs(title = "Boxplot of d15N Values by Age Category",
       x = "Age Category",
       y = expression(delta^{15}*N)) +
  theme_minimal() +
  scale_fill_manual(values = c("Child" = "#FFC857", "Adult" = "#6A4C93"))
```

```
## Warning: Removed 3 rows containing non-finite outside the scale range
## (`stat_boxplot()`).
```

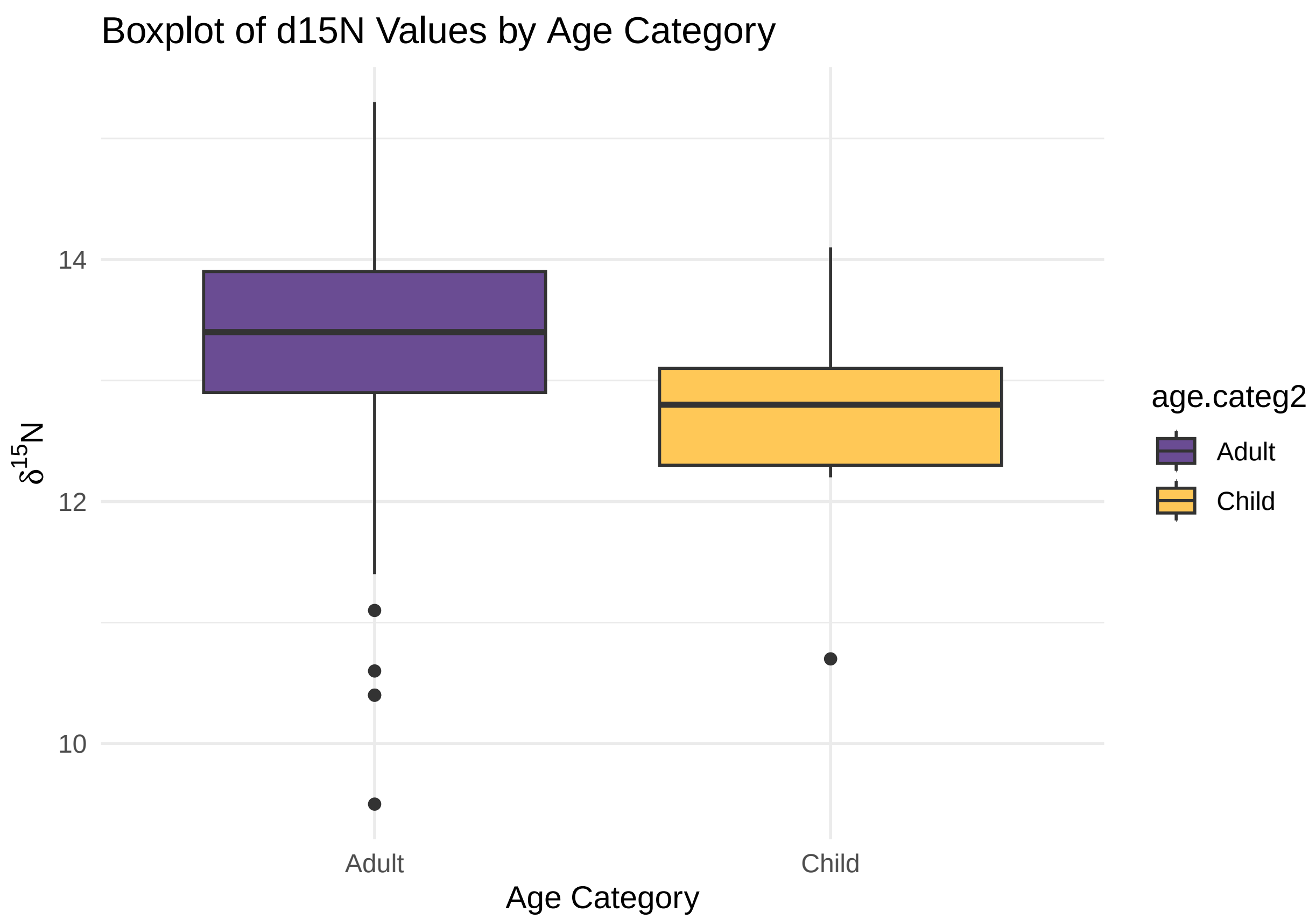

0.15 Section 6: Violence and Physical Stress through time

The Late Intermediate Period (LIP) in the Andean highlands was characterized by intense interpersonal violence and widespread physiological stress linked to environmental and social challenges. The present analysis focuses specifically on individuals with available ^{1}N values, using trauma and skeletal stress markers as contextual evidence for interpreting patterns of dietary and physiological stress over time. The full bioarchaeological dataset, including the broader analyses of cranial trauma, cribra orbitalia, and porotic hyperostosis, is reported elsewhere (McCool et al. 2021, 2023).

Among individuals with nitrogen isotope values, cranial trauma provides important context for elevated ^{1}N values during the LIP. Previous analyses of the broader Nazca highland population documented high trauma prevalence, with cranial fractures interpreted as evidence of direct interpersonal violence and injury during flight or conflict. Trauma occurred across social groups, indicating that violence was widespread rather than restricted to a single subset of the population.

Skeletal stress markers, including porotic hyperostosis (PH) and cribra orbitalia (CO), provide additional context for evaluating nutritional and physiological stress among individuals with ^{1}N data. Although the isotope subset does not independently reproduce the full statistical pattern reported in the broader bioarchaeological dataset, elevated ^{1}N values alongside documented trauma and stress indicators support an interpretation of long-term dietary and environmental challenges during the LIP, particularly in its final phase (1300–1450 CE).

```
Nazca.LIP$cc_trauma <- factor(Nazca.LIP$cc_trauma, levels = c("N", "Y"))
ggplot(data = Nazca.LIP, aes(x = year.CE, y = d15N, color = cc_trauma,
                             shape = cc_trauma)) +
  geom_point(size = 3) +
```

```
  scale_shape_manual(values = c(16, 17),
                     name = "Violence",
                     labels = c("No Trauma", "Trauma")) +
  scale_color_manual(values = c("grey15", "violetred3"),
                     name = "Violence",
                     labels = c("No Trauma", "Trauma")) +
  labs(x = "Year CE", y = expression(delta^{15}*N),
       title = "Scatterplot of d15N with Evidence of Trauma") +
  theme_minimal() +
  theme(legend.position = "bottom") +
  ylim(10, 16)
```

```
## Warning: Removed 8 rows containing missing values or values outside the scale range
## (`geom_point()`).
```

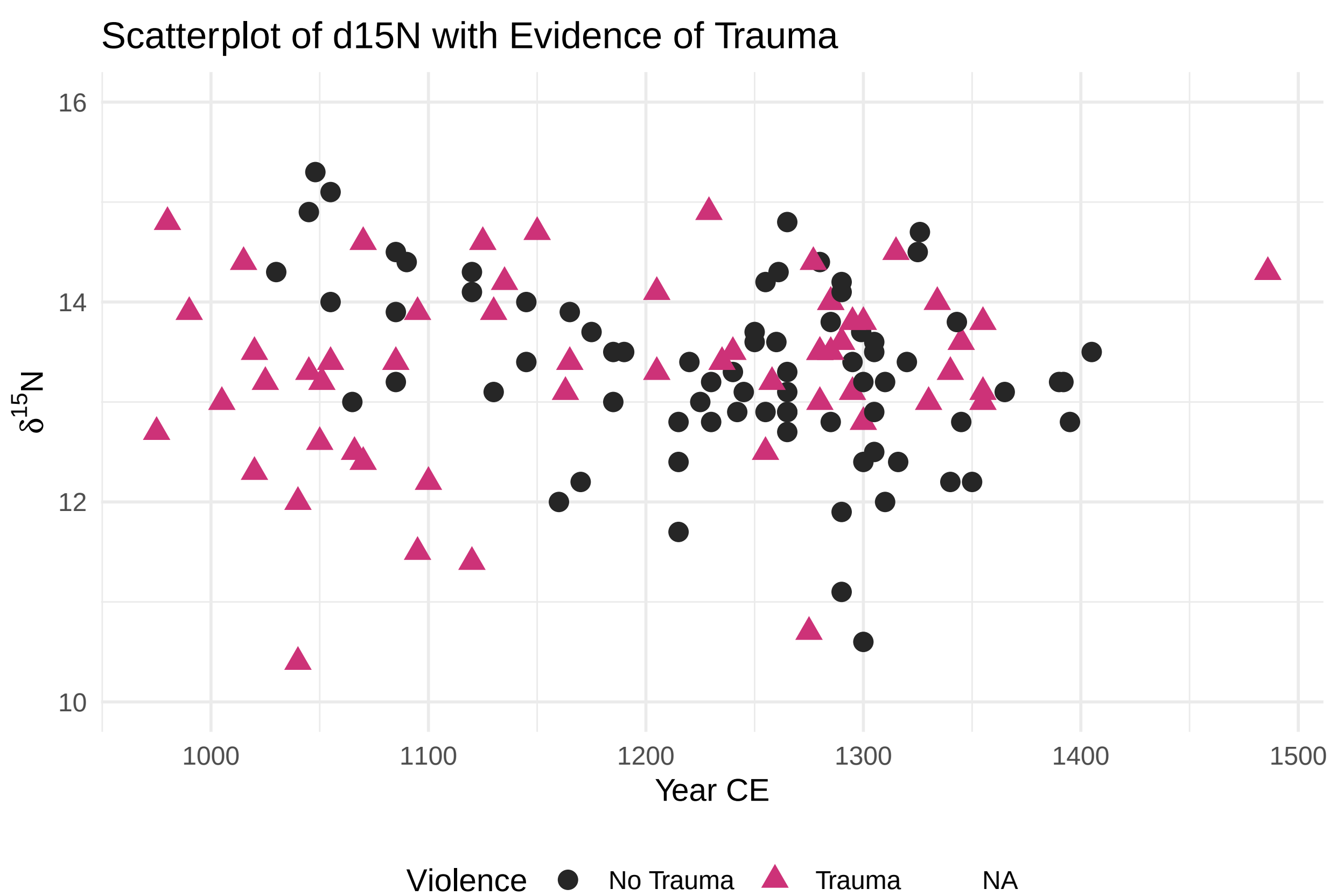


```
Nazca.LIP$shape <- ifelse(Nazca.LIP$porotic == 1, "PH",
                          ifelse(Nazca.LIP$cribra == 1, "CO", "None"))
shape_values <- c("CO" = 17,
                  "PH" = 24,
                  "None" = 16)
color_values <- c("CO" = "violetred4",
                  "PH" = "violetred3",
                  "None" = "black")
ggplot(Nazca.LIP, aes(x = year.CE, y = d15N)) +
```

```
geom_point(aes(shape = shape, color = shape, fill = shape), size = 3) +
scale_shape_manual(values = shape_values,
                   name = "Stress") +
scale_color_manual(values = color_values,
                   name = "Stress") +
scale_fill_manual(values = color_values,
                  name = "Stress") +
labs(x = "Year CE", y = expression(delta^{15}*N),
     title = "Scatterplot of d15N with Stress Indicators") +
theme_minimal() +
theme(legend.position = "bottom") +
ylim(10, 16)
```

```
## Warning: Removed 12 rows containing missing values or values outside the scale range
## (`geom_point()`).
```

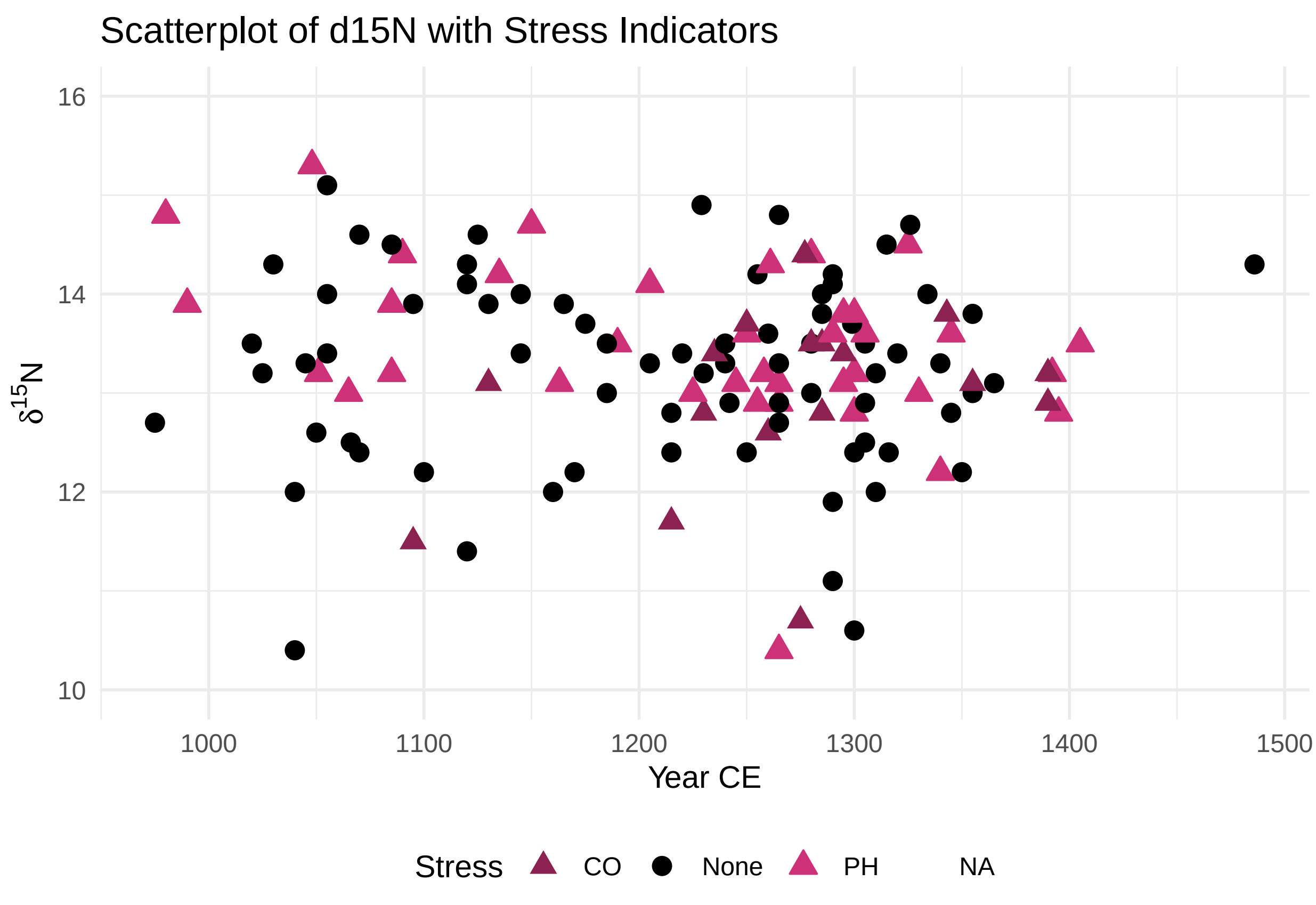

0.16 Section 7: Starvation Overview

In analyzing bone collagen, stable isotope analysis provides valuable insights into past diets and environmental conditions, focusing particularly on nitrogen isotopes ($\delta^{15}N$) as indicators of dietary protein intake. Typically, $\delta^{15}N$ values exhibit a 3-4‰ enrichment per trophic level [1,2]. However, $\delta^{15}N$ values are influenced by non-dietary factors, including the consumption of marine versus terrestrial proteins, the use of manured crops, and physiological conditions such as pregnancy and growth. Additionally, aridity contributes to elevated $\delta^{15}N$ values as water-stressed plants in dry environments pass on higher 15N values to consumers

within those ecosystems. $\delta^{15}N$ enrichment can also result from nutritional stress, such as during starvation when tissue catabolism occurs, making $\delta^{15}N$ a dual indicator of diet and physiological or environmental stress.

Recent applications of stable isotope analysis in the investigation of paleopathologies and nutritional stress have identified nitrogen enrichment as a potential marker of "starvation signatures" [3-7]. Detecting starvation in archaeological remains is challenging as isotopic shifts require time for bone remodeling before death. Notable examples include the Irish Potato Famine and Medieval London, where isotopic evidence aligns with historical records [8,9], although historical documentation often plays a more conclusive role than isotopic data alone. Forensic and animal studies have also documented nitrogen enrichment during starvation, with studies showing a typical 1-1.5% increase in 15N, though no definitive baseline exists for starvation-associated enrichment [4,6].

To ensure precise comparisons of isotopic data from different materials and periods, specific adjustments are necessary. In Neuberger [4], where hair keratin was analyzed instead of the more commonly used bone collagen, a correction of +4.9‰ was applied to standardize $\delta^{15}N$ values according to Mora's guidelines [10]. Furthermore, to account for changes in atmospheric carbon due to industrial activities, a Suess correction of +1.5‰ was applied to modern samples, ensuring that isotopic measurements from various epochs and materials are directly comparable.

References 1. Ambrose, Stanley H. Effects of diet, climate and physiology on nitrogen isotope abundances in terrestrial foodwebs. J. Archaeol. Sci. 18, 293–317 (1991). –> 2. Hedges, Robert E. M., Reynard, Lisa M. Nitrogen isotopes and the trophic level of humans in archaeology. J. Archaeol. Sci. 34, 1240–1251 (2007). –> 3. Eerkens, Jelmer W., Hull, Bryna, Goodman, Jena, Evoy, Angela, Kapp, Joshua D., Hussain, Sidra, Green, Richard E. Stable C and N isotope analysis of hair suggest undernourishment as a factor in the death of a mummified girl from late 19th century San Francisco, CA. PLoS One 12, e0184921 (2017). –> 4. Neuberger, Ferdinand M., Jopp, Eilin, Graw, Matthias, Püschel, Klaus, Grupe, Gisela. Signs of malnutrition and starvation—Reconstruction of nutritional life histories by serial isotopic analyses of hair. Forensic Sci. Int. 226, 22–32 (2013). –> 5. Robertson, Kimberly L., Yarrow, Joshua F., Rowland, Neil E., Krigbaum, John. A rodent model of caloric restriction using bone mass, microarchitecture, and stable isotope ratios: implications for revealing chronic food insu iciency in archaeological populations. STAR: Sci. Technol. Archaeol. Res. 3, 100–111 (2017). DOI:10.1080/20548923.2018.1431863. –> 6. Doi, Hideyuki, Akamatsu, Fumikazu, González, Angélica L. Starvation effects on nitrogen and carbon stable isotopes of animals: an insight from meta-analysis of fasting experiments. Royal Society Open Science 4(8), 170633 (2017). DOI:10.1098/rsos.170633. –> 7. Walter, Brittany S., DeWitte, Sharon N., Dupras, Tosha, Beaumont, Julia. Assessment of nutritional stress in famine burials using stable isotope analysis. Am. J. Phys. Anthropol. 172, 214–226 (2020). DOI:10.1002/ajpa.24054. –> 8. Beaumont, Julia, Montgomery, Janet. The Great Irish Famine: Identifying starvation in the tissues of victims using stable isotope analysis of bone and incremental dentine collagen. PLoS One 11, e0160065 (2016). –> 9. Mora, A. Stable carbon and nitrogen isotope analysis of archaeological human hair: Reconstructing diet and health of ancient individuals. J. Archaeol. Sci.: Rep. 43, 103439 (2022).

```r
#box plots of other contexts plotted with Nazca
ggplot(data = Compiledstarvation, aes(y = delta15N, x = Context, fill = Context)) +
  geom_boxplot() +
  theme_minimal() +
  labs(y = expression(delta^{15}*N)) +
  theme(axis.text.x = element_text(angle = 90, vjust = 0.5)) +
  geom_jitter(shape = 16, position = position_jitter(0.1)) +
  scale_fill_viridis_d(option = "D")
```

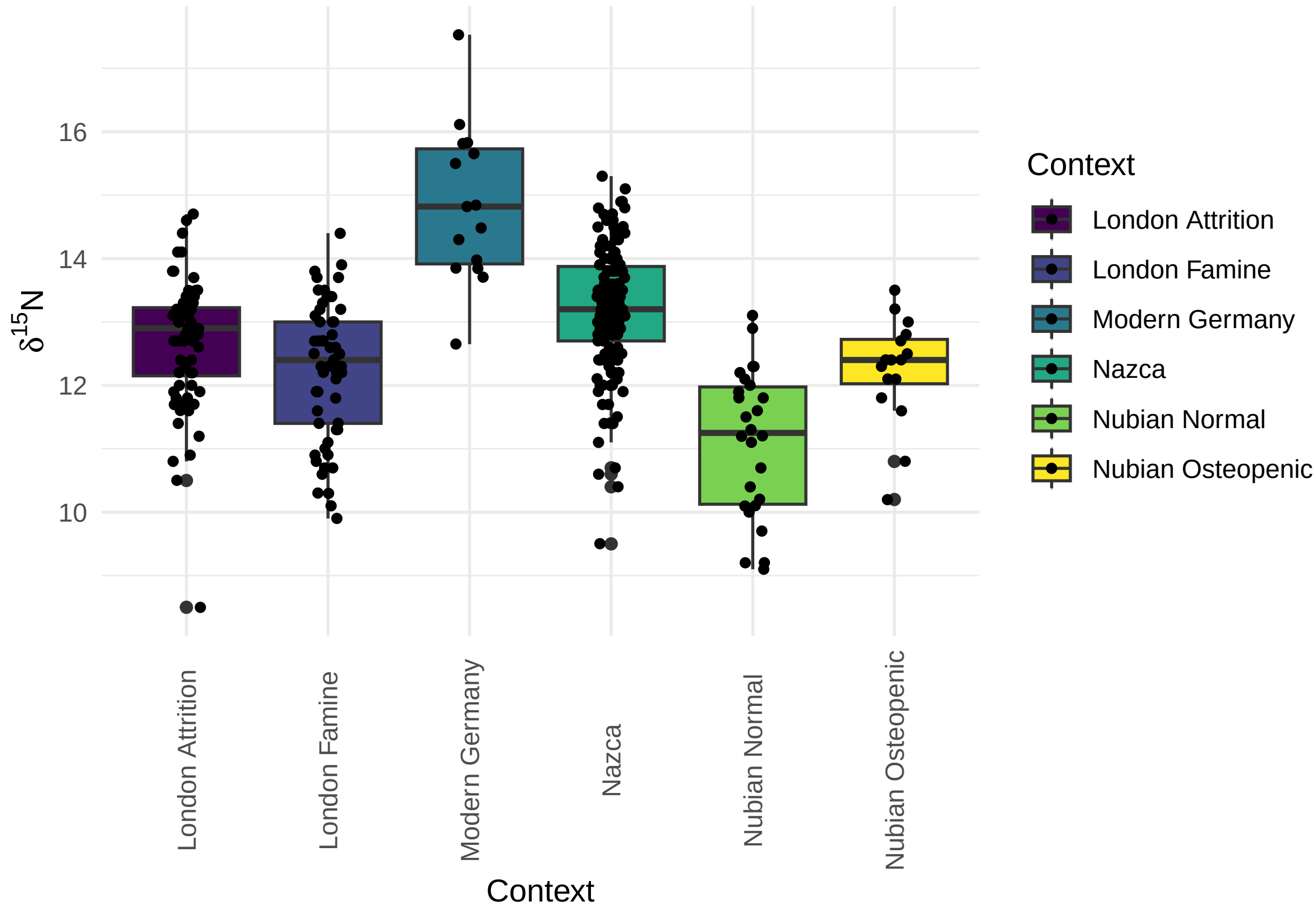
16
14
12
10
$\delta^{15}N$
London Attrition
London Famine
Modern Germany
Nazca
Nubian Normal
Nubian Osteopenic
Context
Context
London Attrition
London Famine
Modern Germany
Nazca
Nubian Normal
Nubian Osteopenic
